# Oxone®-mediated Dakin-like reaction to synthesize hydroxyarenes: an approach using pyrazolo[1,5-*a*]pyrimidines†

**DOI:** 10.1039/d5ra02812d

**Published:** 2025-07-07

**Authors:** Carlos Cifuentes, Marianna Cubides, Jaime Portilla

**Affiliations:** a Bioorganic Compounds Research Group, Department of Chemistry, Universidad de Los Andes Carrera 1 No. 18A-10 Bogotá 111711 Colombia jportill@uniandes.edu.co

## Abstract

A simple and efficient methodology was developed for converting formyl(hetero)arenes into the corresponding hydroxylated derivatives in high yields (95–99%) using Oxone® as the oxidant. This Dakin-like reaction proceeded *via* C–C bond cleavage with the insertion of an oxygen atom into the formyl group, forming the corresponding formyl esters (up to 99%), which then underwent basic hydrolysis to yield the desired alcohols. Although the substrate scope mainly included 3-hydroxypyrazolo[1,5-*a*]pyrimidines, other substrates featuring typical fluorophores (*e.g.*, triphenylamine, anthracene, pyrene, fluorene, and coumarin) were also tested. Moreover, we demonstrated the functionalization of the representative 3-hydroxypyrazolo[1,5-*a*]pyrimidine derivatives obtained using our developed methodology involving alkylation, acylation, and sulfonylation reactions.

## Abstract 2

An Oxone®-mediated reaction of formyl(hetero)arenes to synthesize hydroxylated (hetero)arenes was presented, along with the synthetic applications of some selected products. Products bear typical fluorophores and the pyrazolo[1,5-*a*]pyrimidine core.

## Graphical abstract

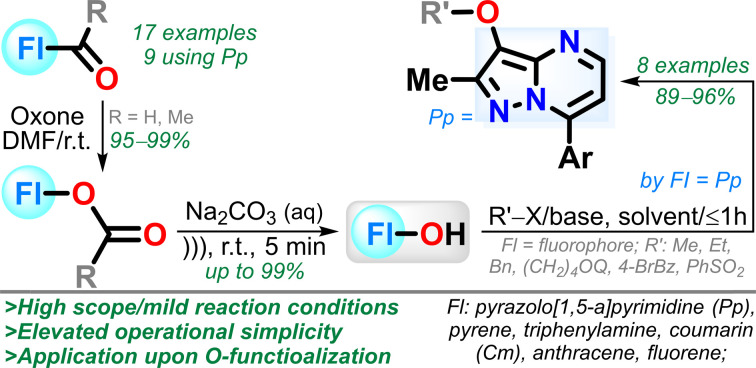

## Introduction

**Fig. 1:**
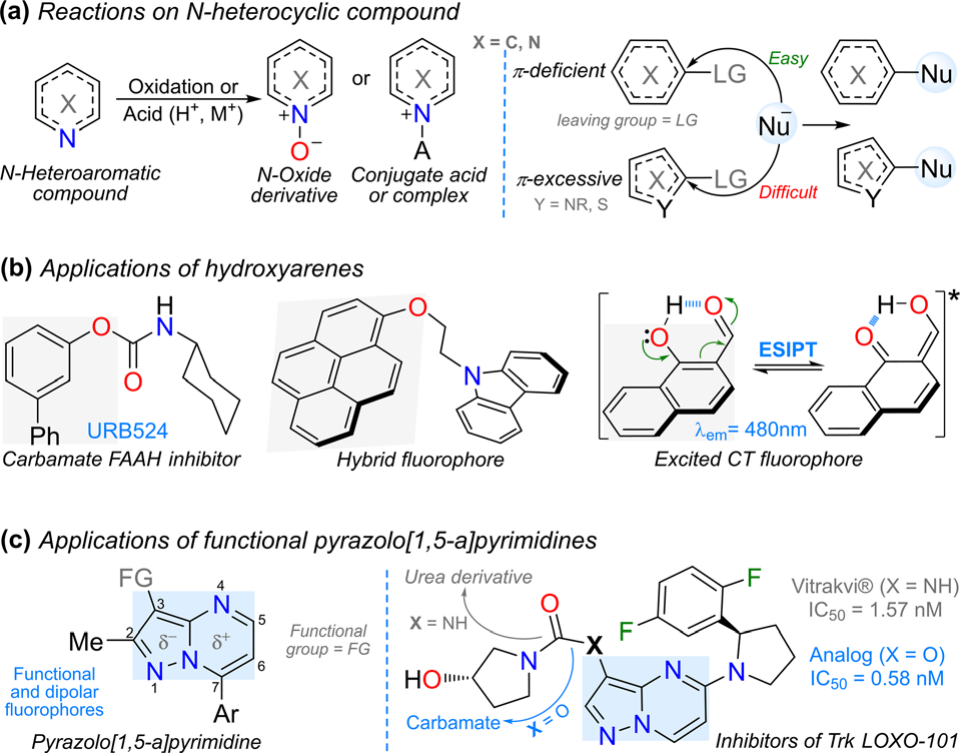
(a) Reactions on (N-hetero) arenes including NAS. (b) Hydroxylated arenes and (c) pyrazolo[1,5-*a*]pyrimidines with relevant applications.

The functionalization reactions of N-heteroaromatic compounds face significant challenges in their organic synthesis as heteroatoms confer unique electronic properties that govern and possibly limit their performance. The reactivity of substrates under acidic, oxidizing, or hydrolytic conditions limits their utility owing to the potential formation of chelates, salts, N-oxides, or ring-opening products.^1–4^ Moreover, the presence of diverse π-excessive or π-deficient rings hinders the incorporation of the desired functional group. For example, a hydroxyl group (OH) is frequently introduced in π-deficient rings using nucleophilic aromatic substitution (NAS) reactions, although this reaction does not usually occur on π-excessive rings;^4–6^ thus, obtaining hydroxylated (N-hetero) arenes is a great challenge (Fig. 1a).^7–9^ Nevertheless, the presence of OH groups in diverse rings is crucial as this functional group allows access to different and relevant chemicals in biological (*e.g.*, carbamates)^10,11^ and photophysical (hybrid dyes)^12,13^ fields. The photophysical field requires the use of fluorophoric reagents, which are highly conjugated or π-excessive rings^12–14^ in which the OH insertion is complex, but its presence facilitates relevant charge transfer (CT) or excited state intramolecular proton transfer (ESIPT) phenomena (Fig. 1b).^15–17^ It is important to note that the synthesis of functional fluorophores is a recurrent objective of our research group,^18,19^ mainly for chemodetection applications,^20^ with relevant results achieved using the pyrazolo[1,5-*a*]pyrimidine (Pp) scaffold;^21^ a dipolar 5 : 6 fused ring system containing three nitrogen atoms essential to synthesize various biologically active compounds (Fig. 1c, left).^4,22–26^

**Scheme 1:**
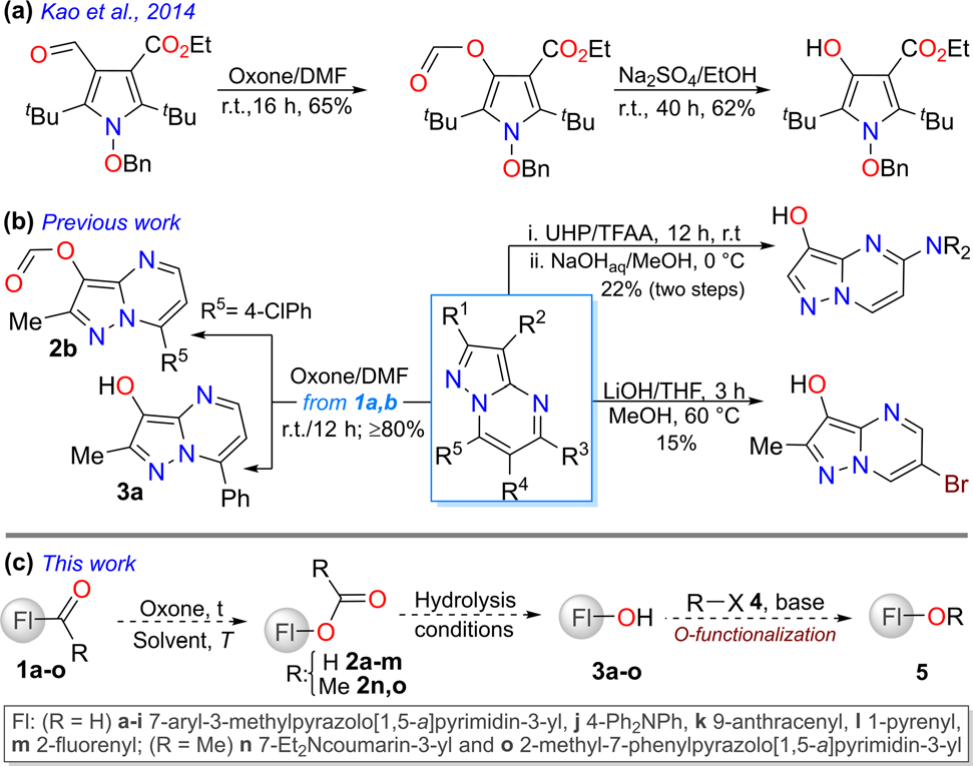
Synthesis of hydroxylated N-heterocyclic compounds (a) by Kao *et al.* and (b) in previous work. (c) Research proposal.

Alternatively, the Baeyer–Villiger (BV) reaction,^27^ using (hetero)arylaldehydes with π-excessive rings as substrates, is a Dakin-like reaction that offers an alternative protocol to access hydroxylated fluorophores. Specifically, a Dakin reaction involves converting hydroxy- or alkoxy-benzaldehydes into the respective phenols.^28^ This transformation is based on the oxidative cleavage of carbon–carbon bonds adjacent to a carbonyl group, with the insertion of an oxygen atom to convert aldehydes or ketones into esters. Peracids (*e.g.*, *m*-CPBA), basic hydrogen peroxide (H_2_O_2_), or acidic urea–hydrogen peroxide (UHP) conditions are generally unsuitable for all substrates as they often lead to overoxidation.^27–29^ Oxone® (a triphasic monopersulfate-based salt: KHSO_5_·½KHSO_4_·½K_2_SO_4_) is a cost-effective, easier-to-handle, efficient, and sustainable oxidizing agent owing to its high solubility in water and decomposition into environmentally friendly waste (K_2_SO_4_) during reactions.^27–30^ Although this reagent has been used in the oxidation of aldehydes to carboxylic acids (*via* the BV reaction) or formyl esters (*via* the Dakin reaction), the latter transformation has been mainly studied for obtaining phenol derivatives from π-excessive benzaldehydes. Remarkably, only two articles were reported in this respect, where Oxone® was preliminarily used on N-heteroaldehydes; in the first one, a 3-formylpyrrole derivative was oxidized to the respective ester to access the desired alcohol (Scheme 1a),^31^ and in the second one, 3-formylpyrazolo[1,5-*a*]pyrimidines 1a–b offered a formyl ester (2b) and an alcohol (3a) (Scheme 1b, left).^32^ The second report was a correction^32^ to an article we published in 2018, in which the reaction was only explored as a preliminary test.^19^

In addition to the Oxone®-mediated Dakin reaction of 1a–b, only one patent was found using the same heterocyclic core, in which the oxidation was performed on a 3-acetylpyrazolopyrimidine using hydrogen peroxide–urea (UHP), but in poor yield; then, the acetyl ester intermediate underwent basic hydrolysis to form the desired alcohol (Scheme 1b, top right).^33^ A similar compound was reported in another patent but *via* a different route, in which a substitution reaction of bromine with a hydroxyl group was carried out in poor yield using lithium hydroxide (Scheme 1b, bottom right).^34^ The first patent reported a biologically valuable alcohol (Fig. 1c, right), as evidenced by the high inhibitory activity of its carbamate derivative (IC_50_ = 0.58 nM) towards Trk (LOXO-101), which proved to be more active than the commercial drug urea derivative Vitrakvi (IC_50_ = 1.57 nM).^33^ Therefore, the Oxone®-mediated Dakin reaction of 1a–b is a promising reaction, despite its result being very preliminary and unexpected.^32^ Indeed, in 2018, we reported the preparation and synthetic utility of 3-formylpyrazolo[1,5-*a*]pyrimidines, in which this reaction was studied to obtain the respective carboxylic acid^19^ under conditions reported by Travis *et al.*^35^ Nevertheless, last year, we attempted to replicate the reaction and irregularities were observed because the ester 2b and alcohol 3a were isolated as oxidation products instead of the desired carboxylic acids (Scheme 1b, left).

Considering this unexpected and fascinating results of the Oxone®-mediated reaction of 1a–b^32^ (substrates bearing an emergent fluorophore^4,21^), we aimed to optimize the reaction conditions that lead to a new series of 3-hydroxypyrazolo[1,5-*a*]pyrimidines 3a–i. We used these alcohols in alkylation, acylation, and sulfonylation reactions (to obtain 5a–h) to explore their synthetic versatility. We also extended the reaction scope to some typical fluorophore-based substrates (*i.e.*, triphenylamine, anthracene, pyrene, fluorene, and coumarin) to yield alcohols 3j–n (Scheme 1c), which are usually difficult to access (due to multistep synthesis and the high cost of commercial compounds)^36–39^ but could facilitate innovative research.^15,16,36–39^

## Results and discussion

Our study to obtain alcohols 3a–i, starting from heteroaldehydes 1a–i, began with the optimization of reaction conditions (solvent, equiv., temperature, and time) using the model substrate 1a under the preliminary conditions of Oxone®/1 equiv. in dry DMF at room temperature (∼20 °C) for 12 h followed by HCl aq workup,^32^ and initially only the reaction time was varied. Although the results were reproducible, the intermediate ester 2a was also isolated under these conditions. Remarkably, the time control facilitated the formation of ester 2a as a single product, and application of microwave irradiation was unfavorable for this reaction (Table 1, entries 1–5). Subsequently, we observed that increasing the temperature and the degree of moisture affected the reaction yield; thus, the reaction was carried out in a closed vessel at ∼20 °C in anhydrous DMF under a nitrogen atmosphere to afford quantitative results. In addition, reducing the equivalents of Oxone® decreased the reaction yield (Table 1, entries 6–8 *vs.* 3), making entry 3 the optimal reaction condition. When we attempted to purify 2a using flash chromatography, the amount of alcohol 3a increased,^32^ obscuring the isolation of 2a; however, the crude ester (a solid residue) was successfully purified by adding cold pentane to remove residual DMF. We also explored changing the solvent to ease the purification process (which was already easy using DMF); however, the reaction only proceeded in MeOH, yielding decomposed products (entry 9 *vs.* 10). Finally, to demonstrate the importance of Oxone® in this transformation, other oxidizing agents (1 equiv. of KOH/H_2_O_2_, *m*-CPBA, and UHP) were used; however, no changes were observed with any of them after 5 hours of reaction at ∼20 °C in dry DMF.

**Table 1:** Optimization of reaction conditions for the Dakin reaction of 1a^a^

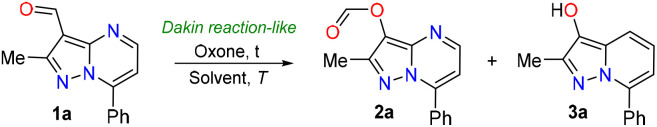					
Entry	Oxone®	Solvent	*t* (h)	*T* (°C)	2a/3a, yield (%)
1	1 equiv.	Dry DMF	12	r.t.	10/85^b^
2			4		80/15^b^
3			2		99/—
4			1		57/—
5			0.5^c^		40/25^b^^,^^d^
6			2	50	73/15^b^^,^^d^
7		DMF	2	r.t.	58/—
8	0.5 equiv.	Dry DMF	5	r.t.	43/—
9	1 equiv.	MeOH	2	r.t.	—/75^d^
10	1 equiv.	DCM, DMSO, or MeCN	5	100	—/—

a Reaction conditions: 50 mg of 1a (0.21 mmol) and Oxone® (32 mg, 1 equiv.) in 1 mL of solvent at r.t.

b Determined by ^1^H NMR using 1,3,5-(MeO)_3_C_6_H_3_ as a standard.

c Under MW in a 10 mL sealed tube.

d Decomposition products were observed.

**Scheme 2:**
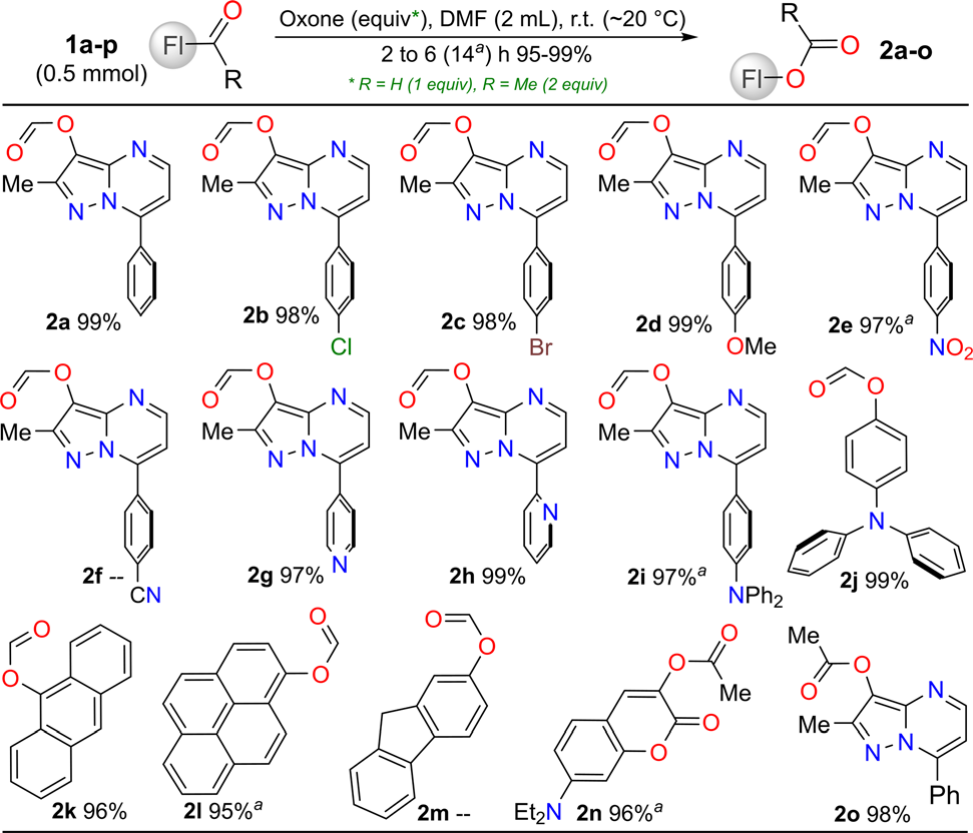
Substrate scope for the synthesis of esters 2a–o.

Subsequently, we studied the reaction scope using substrates 1b–i, which were substituted with a ring of diverse electronic natures at position 7, and notably, only those bearing electron-withdrawing groups (EWGs) affected the reaction time. The reaction was also efficient starting from typical fluorescent aldehydes 1j–m, and even from ketones 1n,o, which required 2 equivalents of Oxone® (Scheme 2). Rings bearing EWGs were expected to affect the migratory capacity of the pyrazolo[1,5-*a*]pyrimidine core during the reaction, generating the desired carboxylic acids;^19^ however, this outcome was not observed, and the respective esters were formed in high yields, indicating that the strong donor effect at position 3 of the heterocyclic core^4,40^ dominated in this oxidation reaction. However, some esters (*i.e.*, 2f and 2m) were not isolated, as they were formed together with traces of alcohol, and these esters were hydrolyzed to the corresponding alcohol during the separation of the mixture using flash chromatography (Scheme 2).^19^

**Scheme 3:**
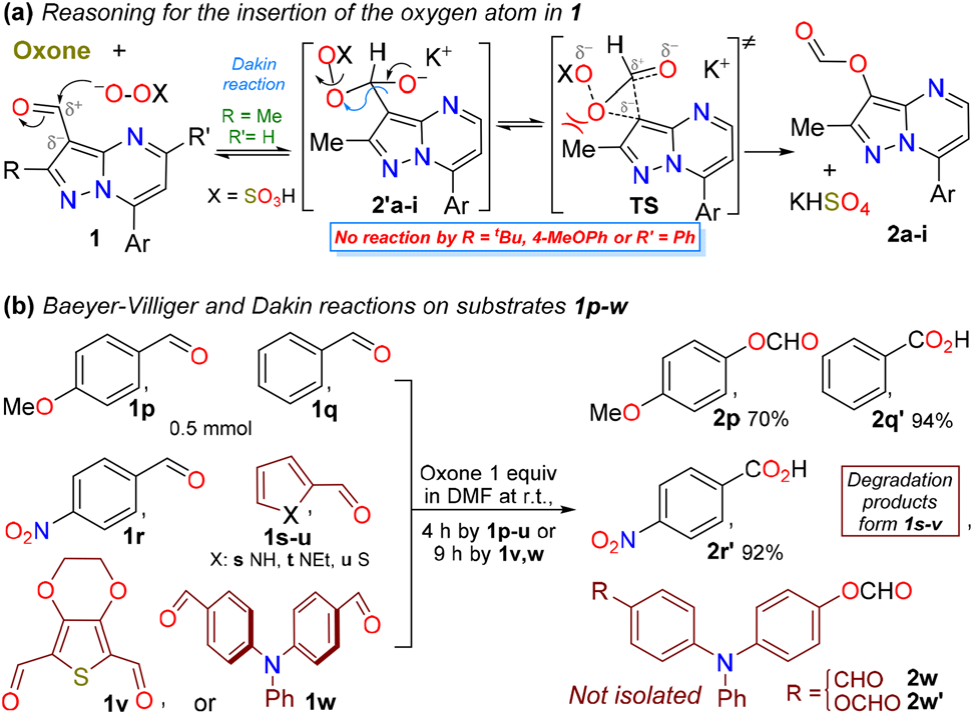
(a) Oxone®-mediated operation on 3-formylpyrazolo[1,5-*a*]pyrimidines 1 and (b) other products obtained from the Oxone®-mediated oxidation reaction.

We also investigated the scope of the reaction by varying the substituents on the heterocyclic core at positions 2 (Me *vs.*^*t*^Bu or 4-MeOPh) and 5 (H *vs.* Ph); however, the reaction did not advanced owing to strong steric effects at the reactive center (the HSO_5_^−^ anion was large) or perhaps owing to the high stability conferred by the substituents (aryl *vs.* Me/H),^4^ which limited the ability to reach the transition state (TS) required during the migration (Scheme 3a). By using other aldehydes available in our laboratory, bearing phenyl 1q, π-excessive (4-MeOPh 1p and pyrrole 1s,t or thiophene 1u) or π-deficient (4-O_2_NPh 1r) rings or two formyl groups 1v,w (Scheme 3b), the Dakin reaction was observed only from 1p,w, while the Baeyer–Villiger oxidation occurred from arylaldehydes 1q,r. Notably, the decomposed products were observed from the reactions of heteroaldehydes 1s–v, possibly owing to their high reactivity towards oxidizing agents (pyrrole and thiophene are π-excessive rings, and 1t,v bear EDGs), and less stable monocyclic structures;^1,5,7^*e.g.*, the Oxone®-mediated reaction reported by Kao *et al.*^31^ from a 3-formylpyrrole derivative yielded the desired ester (Scheme 1a above), likely because the substrate was stabilized by EGWs, such as CO_2_Et and BnO, on the nitrogen atom. Ultimately, the dicarbaldehydes 1v,w were used as substrates to test the selectivity of this transformation; however, only the triphenylamine (TPA) derivative 1w was converted into the corresponding monoester 2w and diester 2w′, since the substrate 1v offered the decomposed products (Scheme 3b). Although intermediate esters 2w and 2w′ were not isolated, they must be hydrolyzed later for discussion of their respective properties.

**Scheme 4:**
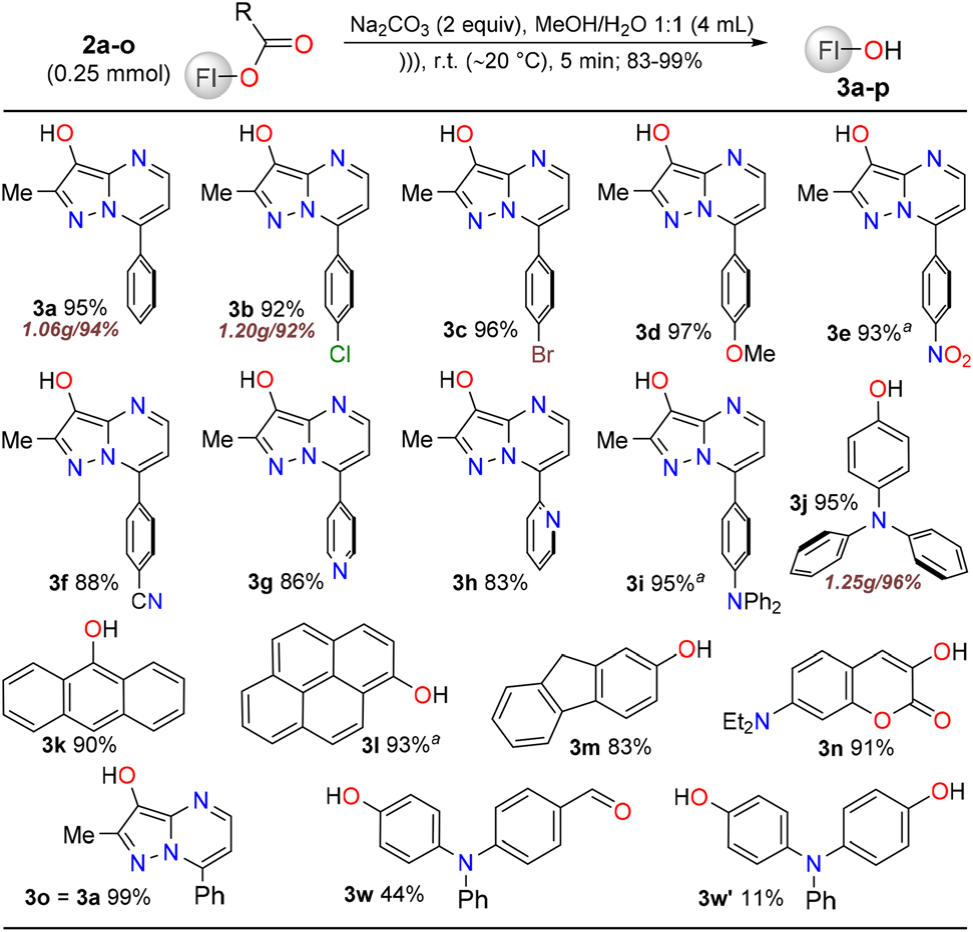
Substrate scope for the synthesis of alcohols 3a–o.

With the formyl 2a–m,w or acetyl 2n,o esters in hand, we then tested their hydrolysis reaction, which was efficiently performed owing to their high reactivity and the presence of a good leaving group (heteroaryloxy). The reaction was carried out in a methanol/water mixture (1 : 3) under basic conditions (2 equiv. of Na_2_CO_3_) and ultrasound (US) irradiation (5 min at ∼20 °C); this hydrolysis reaction occurred efficiently using an ultrasound probe (0.2 MHz, 750 W), as the cavitation effect significantly enhanced the reagent solubility.^41^ Gratifyingly, the products were obtained in excellent yields (up to 99%), and 3a and 3c did not require any further purification beyond extraction (Scheme 4); however, alcohols 3f–h presented challenges during extraction owing to their high solubility in water, resulting in lower yields (83–86%). Finally, the hydroxyaldehyde 3w and the diol 3w′ were obtained when the hydrolysis residue was purified using flash chromatography on silica gel (eluent: (i) DCM and (ii) DCM/MeOH 4 : 1 v/v). The first fraction eluted contained 3w (44%), while the second fraction contained traces of 3w′ (11%); despite the substrate being consumed during oxidation, the moderate isolated yields of these alcohols were owing to their retention on the silica gel during purification. Therefore, the selectivity of the Dakin reaction was established towards the alcohol 3w, possibly due to the use of 1 equivalent of Oxone®. Notably, the TPA derivatives 3w, 3w′, and 3j are highly valuable compounds in materials chemistry^36^ (with 3j costing approximately $116 per gram^42^), anthracen-9-ol (3k) rapidly isomerizes to anthracen-9(10*H*)-one (90%); fluoren-2-ol (3m) has a high commercial cost ($125 per gram (ref. 43)), and the coumarin-3-ol (3n) is a novel dye. Three of these alcohols were obtained on a gram scale (from 5.0 mmol) in 95% (1.07 g/3a), 93% (1.22 g/3b), and 96% (1.25 g/3j) yields.

**Scheme 5:**
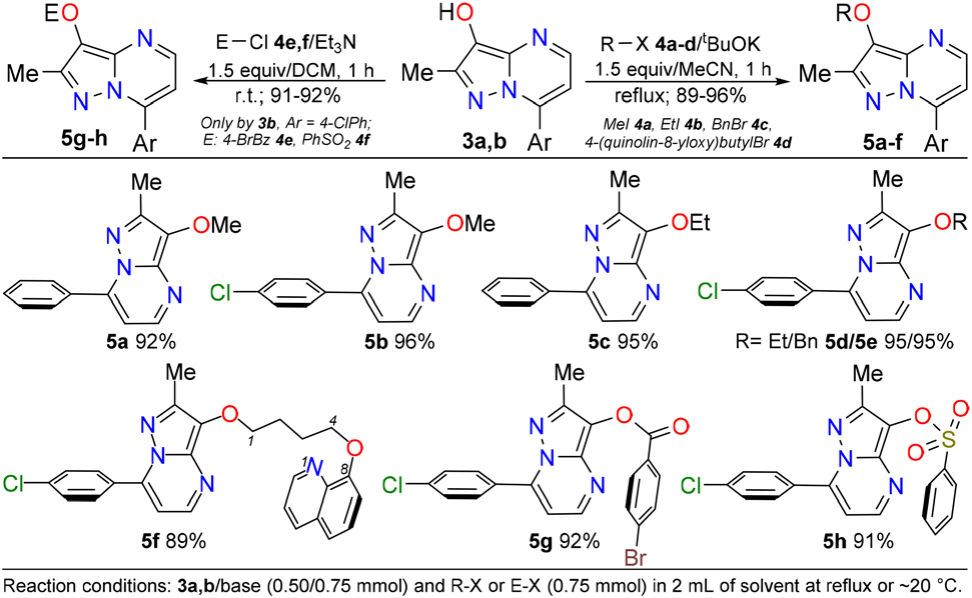
Synthetic applications of 3-hydroxypyrazolo[1,5-*a*]pyrimidine 3a,b.

Finally, we investigated the synthetic application of the obtained alcohols using 3a and 3b in O-functionalizations such as alkylation, acylation, and sulfonylation reactions. The synthesis of ethers 5a–f*via* the reaction of 3a,b with 1.5 equiv. of ^*t*^BuOK/haloalkane (*i.e.*, MeI 4a, EtI 4b, BnBr 4c, or 8-quinolinyl-O(CH_2_)_4_Br 4d) at reflux in MeCN for 1 hour efficiently resulted in high yields (Scheme 5, right); likewise, the acylation and sulfonylation reactions of 3b were successfully realized using Et_3_N/4-BrBzCl 4e or Et_3_N/PhSO_3_H 4f but at ∼20 °C in DCM (Scheme 5, left). These results suggested that the chemistry of 3-hydroxypyrazolo[1,5-*a*]pyrimidines is similar to those of other hydroxylated (hetero)arenes,^35–38^ considering the high nucleophilicity of their OH group.^23^

**Fig. 2:**
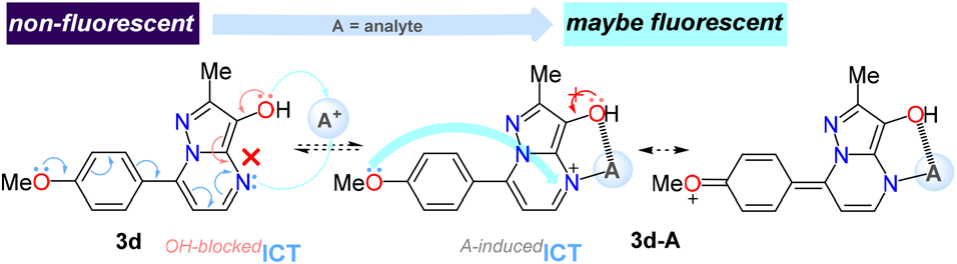
Possible fluorescence process implied in ICT probe 3d.

Notably, unlike the aldehydes 1a–i^19^ and their non-functionalized precursors 6a–i^44^ (see structures in ESI†), none of the hydroxylated pyrazolo[1,5-*a*]pyrimidines 3a–i were found to be fluorescent compounds; this might be possibly owing to the intramolecular charge transfer (ICT) process of the fluorophore family being blocked by the presence of the electron-donating hydroxyl group. Consequently, we aim to explore future applications of this type of alcohols, particularly in chemodetection, through a turn-on fluorescence upon interaction of the hydroxyl group with an analyte of interest that would restore the ICT phenomenon (*e.g.,* using the compound 3d, Fig. 2).

## Conclusions

In summary, the Oxone®-mediated Dakin-type oxidation of 3-formylpyrazolo[1,5-*a*]pyrimidines to 3-hydroxy derivatives was successfully carried out in high yields under mild conditions and using cost-effective reagents. Notably, this synthetic methodology was conveniently extended to other (hetero)arenes bearing typical fluorophores, enabling the preparation of commercially valuable derivatives that are highly expensive but highly promising for photophysical studies. Furthermore, the reaction proved to be selective for the formation of a mono-hydroxylated derivative from a dialdehyde upon controlling the Oxone® equivalents. In addition, the synthetic applications of two 3-hydroxypyrazolo[1,5-*a*]pyrimidines were evaluated using simple methods (alkylation, acylation, and sulfonylation) and readily available reagents. Therefore, the alcohol derivatives obtained herein serve as spearheads for directing novel and varied applications of pyrazolopyrimidines owing to the “almost unprecedented” acquired functionality and the high synthetic and photophysical versatility of this compound family.

## Experimental section

**Comment**. Structures of some novel precursors (*i.e.*, 1e,f), other relevant precursors (*i.e.*, 1n,o and alkylating agent 4d), esters 2a–o, alcohols 3a–w, and O-functionalization products 5a–h were determined using NMR measurements and HRMS analysis (see Fig. S1–S72 in ESI†). Detailed experimental procedures and characterization data for all the synthesised compounds, including their general information, are provided in the ESI.† General experimental procedures for the synthesis of compounds 2a–o, 3a–o, and 5a–h are described below.

### General procedures

#### General procedure for the Dakin-like reaction from (hetero)aryl aldehydes 1a–w

A mixture of aldehyde or ketone 1a–w (0.5 mmol) and Oxone® (76 mg/0.5 mmol for aldehydes or 152 mg/1 mmol for ketones 1n,o) was added into a ball of 5.0 mL and subjected to vacuum for 2 min. Next, the mixture was placed under an N_2_ atmosphere, and anhydrous DMF (2 mL) was added, and the mixture was stirred at ∼20 °C for 2 h. Subsequently, distilled water (5 mL) was added, and the resulting mixture was extracted with DCM (3 × 7 mL). The organic phase was dried over anhydrous MgSO_4_, filtered, and the resulting solution was concentrated under reduced pressure. Finally, 4 mL of cold pentane was added to the residue to remove traces of grease and residual DMF (recrystallisation), affording various pure products. Notably, the reaction of methyl ketones 1n,o required 2 equiv. of Oxone® for complete conversion. In addition, esters 2f, 2m, 2w, and 2w′ (*i.e.*, using substrates with 7-(4-NCPh)Pp 1f or fluorene 1m, or the dialdehyde 1w) were not isolated as they were formed together with traces of the corresponding alcohols; when we tried to purify theses esters, they were hydrolyzed to alcohols during the separation of the mixture using flash chromatography,^19^ making their isolation impossible.

#### General procedure for the hydrolysis of esters 2 to alcohols 3

A mixture of the respective ester 2a–o,w,w′ (0.25 mmol) and Na_2_CO_3_ (53 mg, 0.5 mmol) in MeOH/H_2_O 1 : 1 (4 mL) was subjected to ultrasound irradiation at room temperature for 5 min in a 25 mL TNPSFH glass container. Distilled water (6 mL) was added to the resulting mixture, and it was extracted with DCM (3 × 10 mL). The organic phase was dried over anhydrous MgSO_4_, filtered, and concentrated under reduced pressure. Finally, the residue was purified using flash chromatography (eluent: (i) DCM, (ii) DCM/MeOH 30 : 1 v/v) to afford the desired (hetero)aryl alcohols 3a–o in high yields (83–99%); however, the mixture of hydroxyaldehyde 3w and diol 3w′ was separated using (i) DCM (3w, 44%) and (ii) DCM/MeOH (3w′, 11%) 4 : 1 v/v.

#### General procedure for the synthesis of 3-alkoxy derivatives 5a–f

A mixture of 7-aryl-3-hydroxypyrazolo[1,5-*a*]pyrimidine 3a,b (0.5 mmol) and ^*t*^BuOK (1.5 equiv., 98%, 86 mg) in MeCN (2 mL) was stirred under reflux for 5 min to generate the de alkoxide anion. The alkyl agent 4a–d (1.5 equiv.) was added to the mixture dropwise for 1 min. The reaction mixture was then stirred under reflux for 1 hour. After completion, distilled water (5 mL) was added, and the resulting mixture was extracted with DCM (3 × 10 mL). The organic phase was dried over anhydrous MgSO_4_, filtered, and concentrated under reduced pressure. The residue was purified using flash chromatography (eluent: DCM/MeOH 50 : 1 v/v) to afford the desired products 5a–f in high yields (89–96%).

#### General procedure for synthesising the aroyl 5g and sulphonyl 5h esters

A mixture of nucleophilic reagent 3-hydroxy-7-(4-chlorophenyl)2-methylpyrazolo[1,5-*a*]pyrimidine (3b, 130 mg, 0.5 mmol) and triethylamine (Et_3_N, 99.5%, 105 μL/0.75 mmol) in DCM (2 mL) was stirred for 5 min at room temperature (∼20 °C). Then, either 4-bromobenzoyl chloride (4e 98%, 157 mg, 0.75 mmol) or phenylsulfonyl chloride (4f 99%, 97 μL, 0.75 mmol) was added to the mixture, and it was stirred for 30 min. Afterwards, distilled water (5 mL) was added, and the resulting mixture was extracted using DCM (3 × 10 mL). The organic phase was dried over anhydrous MgSO_4_, filtered, and the solution was concentrated under reduced pressure; the residue was purified using flash chromatography (eluent: DCM/MeOH 50 : 1 v/v) to afford the desired 4-bromobenzoyl 5g (yellow solid, 203 mg, 92%) and phenylsulphonyl 5h (white solid, 182 mg, 91%) esters.

## Author contributions

The individuals listed as authors have contributed to the development of this manuscript, and no other person was involved. The author contributions are as follows: C. C. and M. C. carried out the experiments (synthesis and characterization of products) and conducted literature review, while J. P. developed and composed the original draft, supervised it, and provided sources. All authors have read and agreed to the published version of this manuscript.

## Conflicts of interest

The authors declare no competing financial interest.

## Supplementary Material

RA-015-D5RA02812D-s001

## Data Availability

The data supporting the findings of this study are available within the article and its ESI.† Supporting data for this article are provided in the ESI,† which include the experimental procedures and characterization data, HRMS analysis with spectral copies, and ^1^H NMR, ^13^C NMR, and DEPT-135 spectral copies.
