# Supplementary material for: Oxone®-mediated Dakin-like reaction to synthesize hydroxyarenes: an approach using pyrazolo[1,5-a]pyrimidines
Source: RSC Adv. 2025 Jul 7;15(29):23441–7. doi: 10.1039/d5ra02812d (PMC12230941; doi:10.1039/d5ra02812d)
Supplement: RA-015-D5RA02812D-s001 [file RA-015-D5RA02812D-s001.pdf]

Supplementary Information (SI) for RSC Advances.
This journal is © The Royal Society of Chemistry 2025

Electronic supplementary information (ESI)

Oxone-mediated Dakin-like reaction to form hydroxyarenes: An approach to pyrazolo[1,5-*a*]pyrimidines

Carlos Cifuentes, Marianna Cubides, and Jaime Portilla*

Bioorganic Compounds Research Group, Department of Chemistry, Universidad de Los Andes, Carrera 1 No. 18A-10, Bogotá 111711, Colombia

*Email: jportill@uniandes.edu.co

Content

1. Overview of substrates and products numbering **S2**

2. Experimental procedures **S3**

2.1. General information **S3**

2.2. General procedures **S4**

3. Characterisation data **S6**

4. HRMS analysis **S15**

5. Copies of NMR spectra **S32**

6. References **S70**

1. Overview of substrates and products numbering

(a) Substrates: (hetero)arylaldehydes **1a-m**, methyl ketones **1n,o**, and *O*-substitution reagents **4a-f**

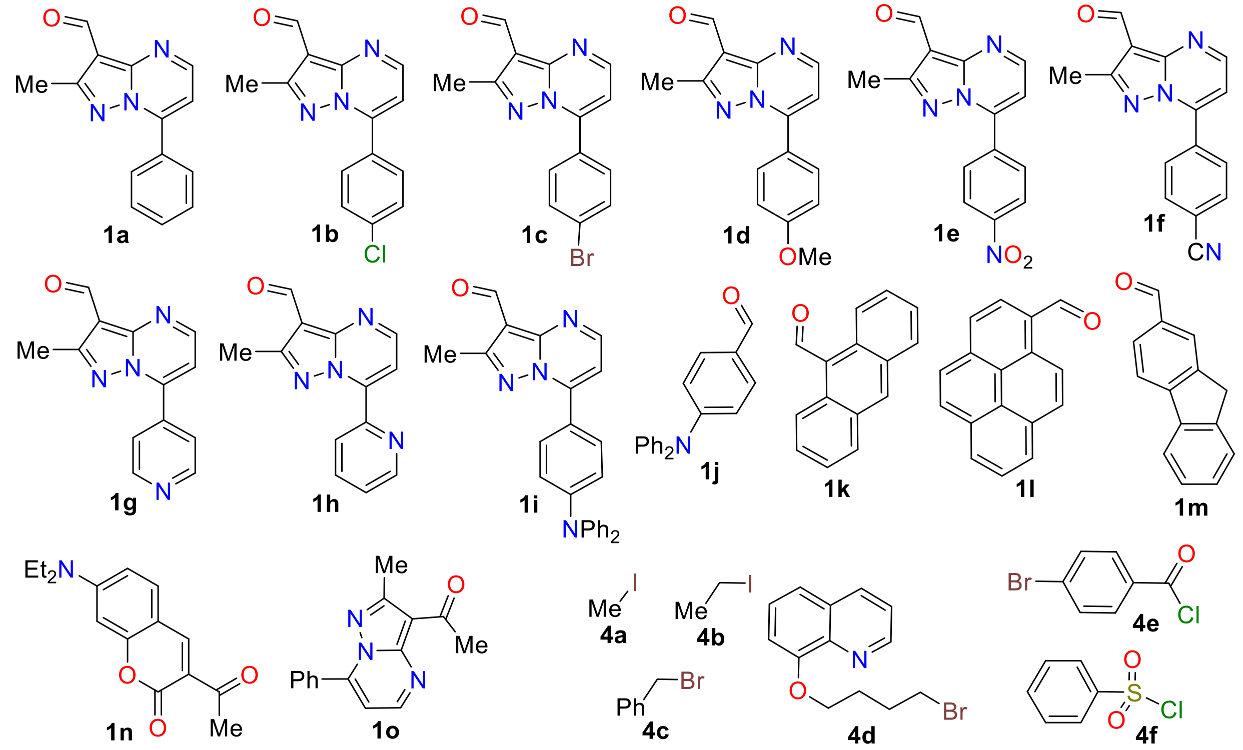

(b) Intermediates: pyrazolo[1,5-*a*]pyrimidin-3-yl formate **1a-i**, and formyl **j-m** and acetyl **1n,o** esters

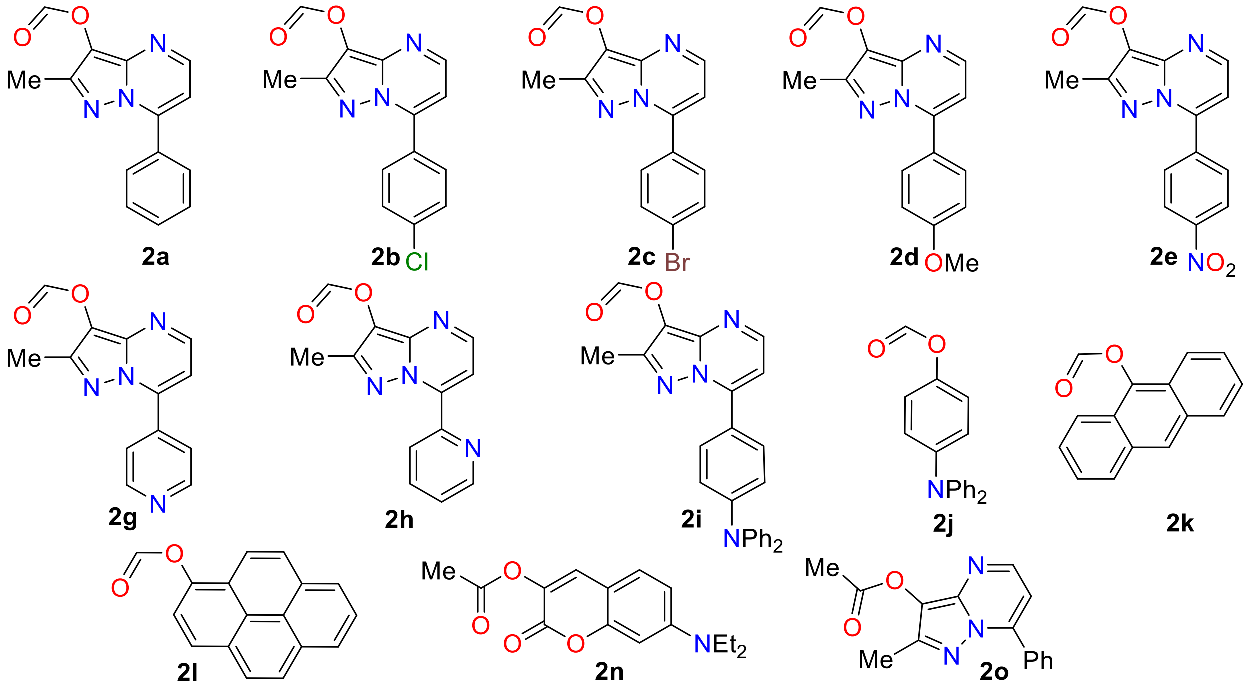

(c) Products: 3-hydroxypyrazolo[1,5-*a*]pyrimidine **1a-i** and other hydroxylated fluorophores **1j-o**

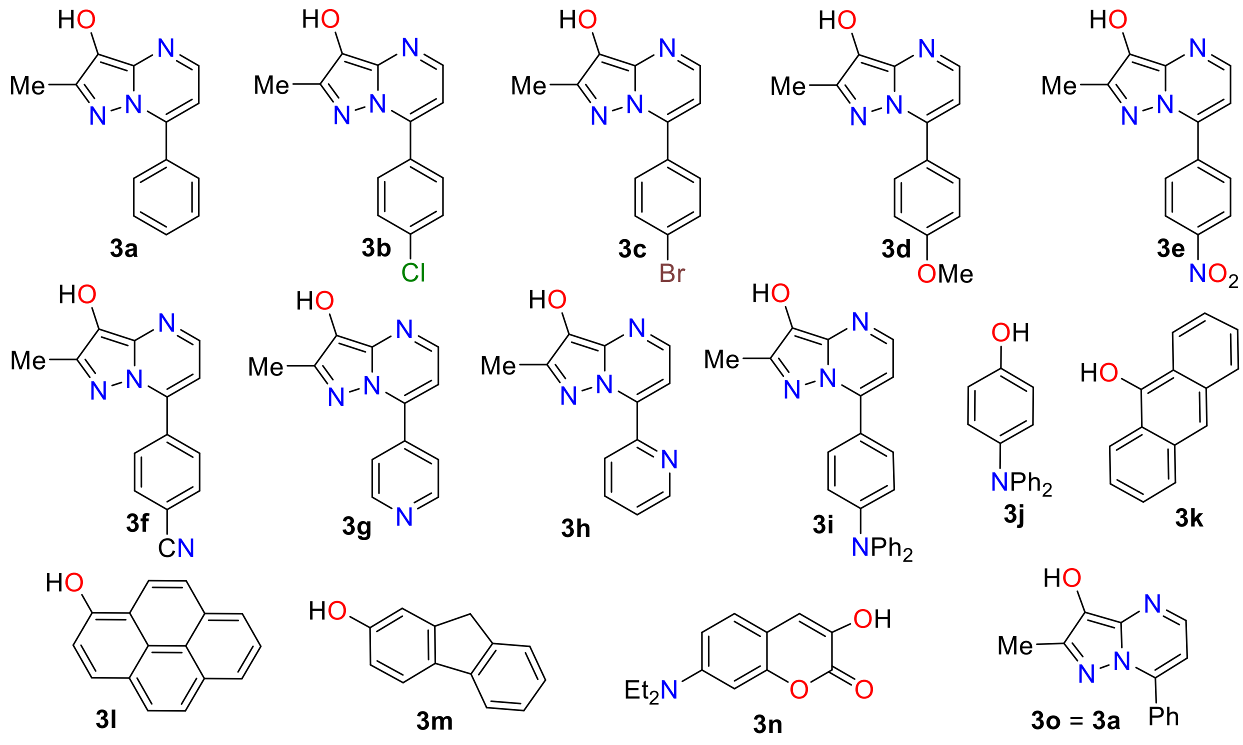

3a 3b 3c 3d 3e 3f 3g 3h 3i 3j 3k 3l 3m 3n 3o = 3a

(d) Alkylated **5a-f**, acylated **5g**, and sulfonylated **5h** 3-hydroxypyrazolo[1,5-*a*]pyrimidines

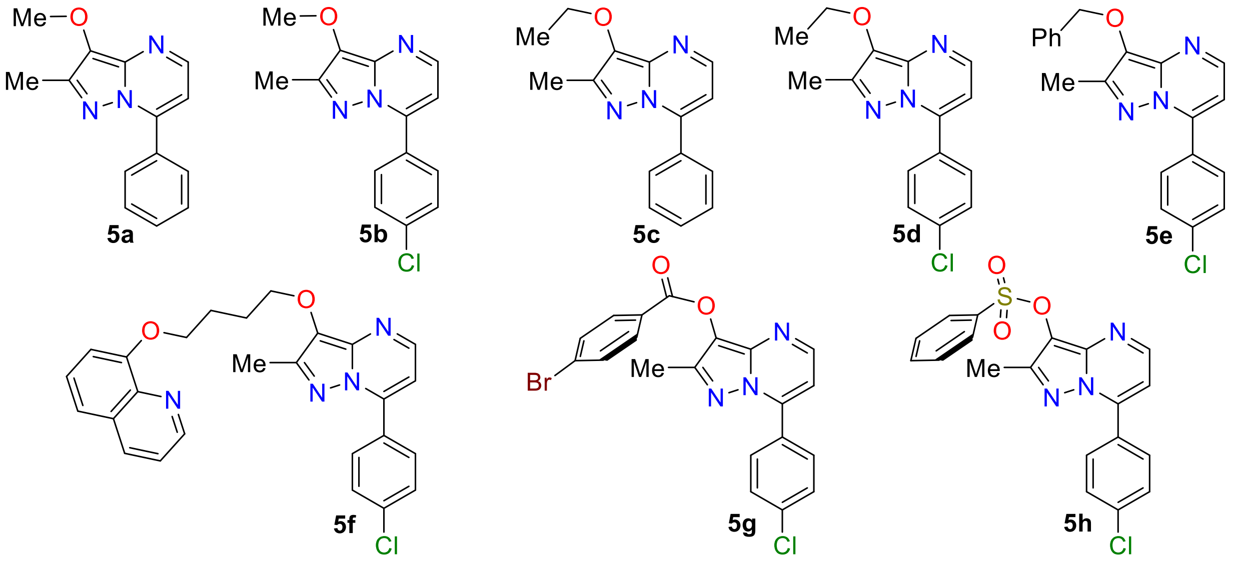

5a 5b 5c 5d 5e 5f 5g 5h

2. Experimental procedures

2.1. General information

All reagents were purchased from commercial sources, used without further purification, and weighed and handled in air at room temperature (~20 °C). The reactions were monitored by TLC

and visualised by a UV lamp (at 254 nm or 365 nm). The Dakin reaction was performed in a Schlenk tube (25 mL) under an N_2 atmosphere and constant stirring. The ester's hydrolysis was performed using an ultrasonic Sonics Vibra-Cell™ VCX 750 probe (0.2 MHz, 750 W) equipped with a tapered microtip of 1⁄4" and a thermocouple; reactions were performed using 50% power amplitude at ~20 °C in a glass two-neck pear-shaped flask (TNPSFH) of 25 ml. Some precursors were prepared under microwaves (MW) in a sealed reaction vessel (10 mL, for max pressure of 300 psi) bearing a Teflon-coated stir bar, and performed in a CEM Discover SP focused MW (v = 2.45 GHz) reactor with a built-in pressure measurement sensor and a vertically focused IR temperature sensor; controlled temperature, power, and time settings were used. *O*-Substitution reactions were developed by conventional heating in a 10 mL Pyrex flask.

NMR spectroscopic data were recorded in $CDCl_3$ and DMSO-d_6 using as internal standards the residual non-deuterated signal ^{1}H NMR (7.26/2.50 ppm in $CDCl_3$/DMSO-d_6) and the deuterated solvent signal for ^{13}C NMR spectroscopy (77.06/39.50 ppm in $CDCl_3$/DMSO-d_6).[1] DEPT-135 experiments were used for the carbon signals assignment (CH, CH_2, and CH_3). The following abbreviations are used for multiplicities: s = singlet, d = doublet, t = triplet, q = quartet, and m = multiplet. Chemical shifts (δ) are given in ppm and coupling constants (J) in Hz. High-resolution mass spectra (HRMS) were obtained on an Agilent Technologies Q-TOF 6520 spectrometer by an electrospray ionisation (ESI, 4000 V). Melting points were determined in capillary tubes on a Stuart SMP10 melting point apparatus and were uncorrected.

2.2. General procedures

2.2.1. Precursors synthesis

*3-Formylpyrazolo[1,5-a]pyrimidines **1a-j***

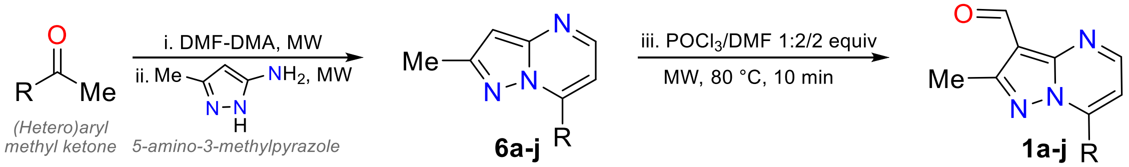

Aldehydes **1a-j** were obtained using three MW-assisted reaction steps[2–6] involving *i. β*-enaminones formation (from methyl ketones and DMF-DMA), *ii.* their cyclocondensation with 5-amino-3-methylpyrazole, and *iii.* formylation reaction of the respective cyclisation product **6a-j**; the last step is described below. A mixture of $POCl_3$ (2 mmol, 187 µL) and 310 µL of anhydrous *N,N*-dimethylformamide (4 mmol) was added into a sealed tube containing a Teflon-coated magnetic stirring bar, cooled to 0 °C and stirred for 30 min. Later, a solution of **6a-j** (1 mmol) in

300 μL of DMF under stirring at 0 °C for 10 min was added dropwise, and the reaction was warmed to ~20 °C and stirred for 15 min; finally, the mixture was irradiated with MW at 80 °C (100 W) for 10 min in a sealed tube under stirring. The resulting reaction mixture was cooled to 50 °C by airflow, was maintained at pH = 7 by adding an aqueous solution of $NaHCO_3$ (20%), and was vigorously stirred at ~20 °C for 30 min. The formed precipitate was filtered, washed with cold water (3 × 4 mL), and purified by flash chromatography on silica gel (eluent DCM) to afford the pure products **1a-j**. The characterisation data for **1e,f** (R = 4-O_2NPh, 4-NCPh) have not been previously reported by us, so the respective data are given in the next section.

*3-Acetyl-7-diethylaminocoumarin **1o***

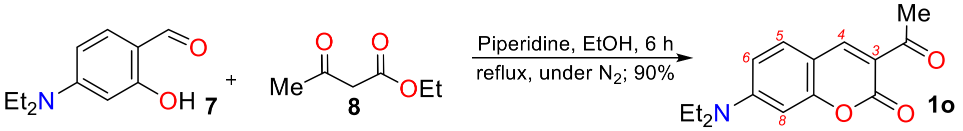

In a 10 mL flask, 4-(diethylamino)salicylaldehyde (**7**, 290 mg, 1.5 mmol) was dissolved in absolute EtOH (2.0 mL), and 99% ethyl acetoacetate (**8**, 387 μL, 3.0 mmol) and 99% piperidine (9 μL, 6% mol) were added; after, the reaction was refluxed for 6 h under N_2 and then, the resulting mixture was allowed to cool to room temperature (~20 °C). The solid formed from the mixture was filtered and washed with *n*-pentane to obtain the crude product, which was then recrystallised from ethanol to afford the desired methyl ketone as a yellow solid (Mp: 155 °C) in 90% yield (354 mg, CAS: 74696-96-1). ^{1}H NMR (400 MHz, $CDCl_3$): δ = 1.24 (t, *J* = 7.1 Hz, 6H), 2.68 (s, 3H), 3.45 (q, *J* = 7.1 Hz, 4H), 6.47 (d, *J* = 2.3 Hz, 1H*8*), 6.62 (dd, *J* = 8.9 Hz and 2.4 Hz, 1H*6*), 7.39 (d, *J* = 8.9 Hz, 1H*5*), 8.43 (s, 1H*4*) ppm. These experimental data matched with previously reported data by us.[7]

*3-Acetyl-7-fenyl-3-methylpyrazolo[1,5-a]pyrimidine **1p***

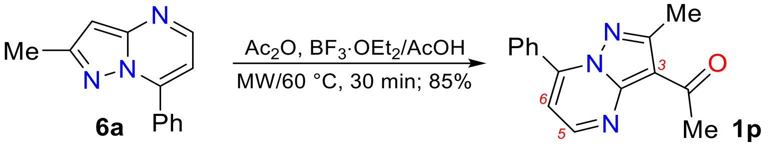

A mixture of 3-methyl-7-phenylpyrazolo[1,5-*a*]pyrimidine (**1a**, 209 mg 1 mmol), boron trifluoride etherate ($BF_3 \cdot OEt_2$, 1 mL, ∼8 equiv), and acetic anhydride (Ac_2O, 1 mL ∼10 equiv) in acetic acid (2 mL) was irradiated with microwaves at 60 °C (70 W) for 30 min under stirring in a sealed tube (10 mL) bearing a magnetic stir bar. After, the reaction was cooled to ~20 °C by airflow, and cold water (2.0 mL) was added to precipitate the product, which was filtered, washed, and dried under a high vacuum to give **1p** as an orange solid (Mp: 184 °C) in 85% yield (214 mg). ^{1}H NMR (400

MHz, $CDCl_3$): δ = 2.73 (s, CH_3), 2.86 (s, CH_3), 7.02 (d, J = 4.4 Hz, 1H*6*), 7.56–762 (m, 3H), 8.03 (m, 2H), 8.68 (d, J = 4.4 Hz, H*5*) ppm. These data matched with previously reported data.[4]

*8-(4-Bromobutoxy)quinoline **4d***

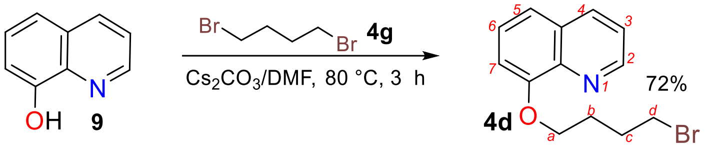

A mixture of quinolin-8-ol (**9**, 145 mg, 1 mmol), 1,4-dibromobutane (**4g**, 259 mg, 1.2 mmol), and Cs_2CO_3 (391 mg, 1.2 mmol) in 3 mL of DMF was added into a ball with a magnetic stir bar and was heated at 80 °C for 3 h; then, the mixture was allowed to cool to ~20 °C and it was extracted with DCM (4 × 5 mL). After combining the organic layers, the solution was washed with brine (2 × 10 mL), dried on anhydrous Na_2SO_4, filtered, the filtrate concentrated under a vacuum, and the residue was purified by flash chromatography (eluent: DCM). The alkyl bromide **4d** was obtained as a yellow oil (202 mg, 72%). ^{1}H NMR (400 MHz, $CDCl_3$): δ = 2.16 (s-br, 2CH_2 *b/c*), 3.53 (s-br, CH_2 *a*), 4.27 (s-br, CH_2 *d*), 7.05 (d, J = 7.6 Hz, 1H*7*), 7.35–7.49 (m, 3H), 8.12 (d, J = 8.2 Hz, 1H*4*), 8.93 (d, J = 2.7 Hz, 1H*2*) ppm. These experimental data matched with previously reported data.[8]

3. Data characterisation

*2-Methyl-7-(4-nitrophenyl)pyrazolo[1,5-a]pyrimidine-3-carbaldehyde **1e**.* By the general method

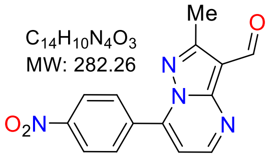

from **6e** (254 mg, 1 mmol), **1e** was obtained as a yellow solid (215 mg, 76%). Mp 244–246 °C. ^{1}H NMR (400 MHz, DMSO-d_6): δ = 2.60 (s, 3H), 7.58 (d, J = 4.1 Hz, 1H), 8.32 (d, J = 8.4 Hz, 2H), 8.44 (d, J = 8.4 Hz, 2H), 8.91 (d, J = 4.1 Hz, 1H), 10.29 (s, 1H) ppm. ^{13}C NMR (101 MHz, DMSO-d_6) δ = 14.3 (CH_3), 108.2 (C), 111.2 (CH), 123.6 (CH), 131.3 (CH), 135.9 (C), 144.8 (C), 148.8 (C), 151.54 (C), 153.5 (CH), 155.8 (C), 183.2 (CH) ppm; HRMS (ESI) m/z calcd for $C_{14}H_{11}N_4O_3^+$ $[M + H]^+$ 283.0826, found 283.0824.

*4-(3-Formyl-2-methylpyrazolo[1,5-a]pyrimidin-7-yl)benzonitrile **1f**.* By the general method from 2-

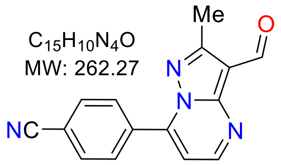

methyl-7-(4-cyanophenyl)pyrazolo[1,5-*a*]pyrimidine (**6f**, 234 mg, 1 mmol), **1f** was obtained as a yellow solid (163 mg, 62%). Mp 247–248 °C. ^{1}H NMR (400 MHz, DMSO-d_6): δ = 2.59 (s, 3H), 7.50 (d, J = 4.2 Hz, 1H), 8.07 (d, J = 8.1 Hz, 2H), 8.21 (d, J = 8.0 Hz, 2H), 8.86 (d, J = 4.2 Hz, 1H), 10.26 (s, 1H) ppm. ^{13}C NMR (101 MHz, DMSO-d_6) δ = 14.6 (CH_3), 108.6 (CH), 111.5 (C), 114.1 (C), 131.0 (CH), 132.9 (CH), 134.6 (C), 145.4 (C), 150.2 (C), 154.0 (CH), 156.4 (C), 183.9 (CH) ppm; HRMS (ESI) m/z calcd for $C_{15}H_{11}N_4O$ $[M + H]^+$ 263.0927, found 263.0924.

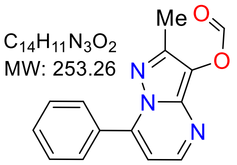

*2-Methyl-7-phenylpyrazolo[1,5-a]pyrimidin-3-yl formate **2a**.* This ester was obtained as a yellow solid (125 mg, 99%) from **1a** (119 mg, 0.5 mmol) via the general procedure for 2 h. Mp 145–146 °C. ^{1}H NMR (400 MHz, $CDCl_3$): δ = 2.44 (s, 3H), 6.86 (d, *J* = 4.3 Hz, 1H), 7.58 (m, 3H), 8.03 (m, 2H), 8.47/8.48 (s/s, 2H) ppm. ^{13}C NMR (101 MHz, $DCDCl_3$) δ = 11.8 (CH_3), 107.2 (CH), 120.8 (C), 127.4 (C), 128.8 (CH), 129.3 (CH), 130.3 (C), 131.4 (CH), 140.6 (C), 146.4 (C), 149.9 (CH), 158.9 (CH) ppm; HRMS (ESI) m/z calcd for $C_{14}H_{12}N_3O_2^+$ $[M + H]^+$ 254.0924, found 254.0926.

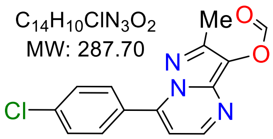

*7-(4-Chlorophenyl)-2-methylpyrazolo[1,5-a]pyrimidin-3-yl formate **2b**.* This ester was obtained as a yellow solid (141 mg, 98%) from **1b** (136 mg, 0.5 mmol) via the general procedure for 4 h. Mp 177–178 °C. ^{1}H NMR (400 MHz, $CDCl_3$): δ = 2.44 (s, 3H), 6.84 (d, *J* = 4.1 Hz, 1H), 7.55 (d, *J* = 8.3 Hz, 2H), 8.00 (d, *J* = 8.3 Hz, 2H), 8.46/847 (s/s, 2H) ppm. ^{13}C NMR (101 MHz, $DCDCl_3$) δ = 11.8 (CH_3), 107.0 (CH), 120.9 (C), 128.6 (C), 129.1 (CH), 130.7 (CH), 137.5 (C), 140.6 (C), 145.2 (C), 146.5 (C), 149.0 (CH), 158.7 (CH) ppm. HRMS (ESI+): calcd. for $C_{14}H_{11}{}^{35}ClN_3O_2^+$ 288.0534 $[M + H]^+$; found 288.0544. These data matched with previously reported data by us.[9]

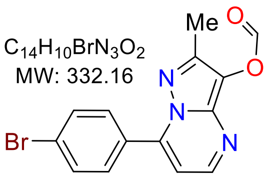

*7-(4-Bromophenyl)-2-methylpyrazolo[1,5-a]pyrimidin-3-yl formate **2c***. This ester was obtained as a yellow solid (162 mg, 98%) from **1c** (158 mg, 0.5 mmol) via the general method for 6 h. Mp 196–197 °C. ^{1}H NMR (400 MHz, $CDCl_3$): δ = 2.43 (s, 3H), 6.82 (d, *J* = 4.3 Hz, 1H), 7.70/7.92 (d/d, *J* = 8.6 Hz, 4H), 8.45/8.47 (s/s, 2H) ppm. ^{13}C NMR (101 MHz, $CDCl_3$): δ = 11.8 (CH_3), 107.0 (CH), 120.9 (C), 126.0 (C), 129.1 (C), 130.8 (CH), 132.1 (CH), 140.6 (C), 145.2 (C), 146.6 (C), 149.0 (CH), 158.8 (CH) ppm. HRMS (ESI+): calcd. for $C_{14}H_{11}{}^{79}BrN_3O_2^+$ 332.0029 $[M + H]^+$; found 332.0026.

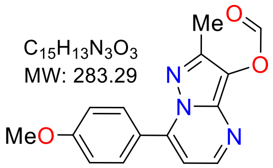

*7-(4-Methoxyphenyl)-2-methylpyrazolo[1,5-a]pyrimidin-3-yl formate **2d***. By the general method (4 h) from 3-formyl-7-(4-methoxyphenyl)-2-methylpyrazolo[1,5-*a*]pyrimidine (**1d**, 134 mg, 0.5 mmol), **2d** was obtained as a yellow solid (140 mg, 99%). Mp 163–164 °C. ^{1}H NMR (400 MHz, $CDCl_3$): δ = 2.44 (s, 3H), 3.89 (s, 3H), 6.82 (d, *J* = 4.1 Hz, 1H), 7.06 (d, *J* = 8.5 Hz, 2H), 8.06 (d, *J* = 8.5 Hz, 2H), 8.43 (m, 2H) ppm. ^{13}C NMR (101 MHz, $CDCl_3$): δ = 11.8 (CH_3), 55.5 (CH_3), 106.4 (CH), 114.2 (CH), 120.6 (C), 122.4 (C), 131.1 (CH), 140.1 (C), 146.1 (C), 146.2 (C), 149.0 (C), 158.9 (CH), 162.0 (C) ppm. HRMS (ESI+): calcd. for $C_{15}H_{14}N_3O_3^+$ 284.1030 $[M + H]^+$; found 284.1038.

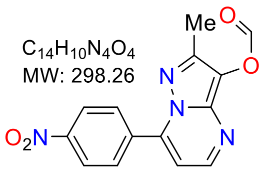

*2-Methyl-7-(4-nitrophenyl)pyrazolo[1,5-a]pyrimidin-3-yl formate **2e*** was obtained as a yellow solid (144 mg, 97%) from **1e** (141 mg, 0.5 mmol) via the general method for 14 h. Mp 162–163 °C. ^{1}H NMR (400 MHz, $CDCl_3$): δ = 2.44 (s, 3H), 6.92 (d, *J* = 4.2 Hz, 1H), 8.23 (d, *J* = 8.7 Hz, 2H), 8.42 (m, 3H), 8.52 (d, *J* = 4.2 Hz, 1H) ppm. ^{13}C NMR (101 MHz, $CDCl_3$): δ = 11.8 (CH_3), 107.7 (CH), 121.2 (C), 123.9 (CH),

130.5 (CH), 136.2 (CH), 140.6 (C), 143.7 (C), 147.0 (C), 148.9 (CH), 149.2 (C), 158.6 (CH) ppm. HRMS (ESI+): calcd. for $C_{14}H_{11}N_4O_4^+$ 299.0775 [M + H]$^+$; found 238.0772.

2-Methyl-7-(4-pyridyl)pyrazolo[1,5-a]pyrimidin-3-yl formate ***2g****.* This ester was obtained as a yellow solid (123 mg, 97%) from **1g** (119 mg, 0.5 mmol) via the general method for 6 h. Mp 187–188 °C. ^{1}H NMR (400 MHz, $CDCl_3$): δ = 2.44 (s, 3H), 6.92 (d, *J* = 4.2 Hz, 1H), 7.93 (m, 2H), 8.45 (s, 1H), 8.51 (d, *J* = 4.2 Hz, 1H), 8.85 (m, 2H) ppm. ^{13}C NMR (101 MHz, $CDCl_3$): δ = 11.8 (CH_3), 107.4 (CH), 121.2 (C), 123.1 (CH), 137.8 (C), 140.5 (C), 143.42 (C), 146.9 (C), 148.9 (CH), 149.0 (C), 150.5 (CH), 158.6 (CH) ppm. HRMS (ESI+): calcd. for $C_{13}H_{11}N_4O_2^+$ 255.0876 [M + H]$^+$; found 255.0876.

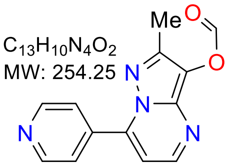

2-Methyl-7-(2-pyridyl)pyrazolo[1,5-a]pyrimidin-3-yl formate ***2h****.* By the general procedure from the 7-(2-pyridyl) derivative (**1h**, 119 mg, 0.5 mmol) for 6 h, **2h** was obtained as a yellow solid (125 mg, 99%). Mp 150–151 °C. ^{1}H NMR (400 MHz, $CDCl_3$): δ = 2.48 (s, 3H), 7.46 (m, 1H), 7.64 (d, *J* = 4.3 Hz, 1H), 7.93 (m, 1H), 8.47 (s, 1H), 8.55 (d, *J* = 4.3 Hz, 1H), 8.80 (d, *J* = 4.0 Hz, 1H), 9.04 (d, *J* = 8.0 Hz, 1H) ppm. ^{13}C NMR (101 MHz, $CDCl_3$): δ = 11.8 (CH_3), 107.8 (CH), 120.9 (C), 125.6 (CH), 126.1 (CH), 136.7 (CH), 141.0 (C), 143.6 (C), 146.1 (C), 147.8 (C), 149.1 (CH), 150.1 (CH), 158.8 (CH) ppm. HRMS (ESI+): calcd. for $C_{13}H_{11}N_4O_2^+$ 255.0876 [M + H]$^+$; found 255.0873.

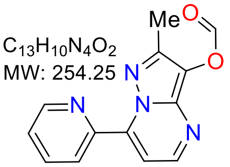

7-(4-Diphenylaminophenyl)-2-methylpyrazolo[1,5-a]pyrimidin-3-yl formate ***2i****.* This ester was obtained as a yellow solid (203 mg, 97%) from **1i** (202 mg, 0.5 mmol) via the general method for 2 h. Mp 134–136 °C. ^{1}H NMR (400 MHz, $CDCl_3$): δ = 2.44 (s, 3H), 6.83 (d, *J* = 4.3 Hz, 1H), 7.13 (m, 4H), 7.20 (m, 4H), 7.33 (m, 4H), 8.00 (d, *J* = 8.7 Hz, 2H), 8.44 (m, 2H) ppm. ^{13}C NMR (100 MHz, $CDCl_3$): δ = 11.8 (CH_3), 106.9 (CH), 120.5 (CH), 122.0 (C), 124.5 (CH), 125.9 (CH), 129.6 (CH), 130.4 (CH), 140.9 (C), 146.0 (C), 146.1 (C), 146.7 (C), 148.9 (CH), 150.7 (C), 159.0 (CH) ppm. HRMS (ESI+): calcd. for $C_{26}H_{21}N_4O_2^+$ 421.1659. [M + H]$^+$; found 421.1646.

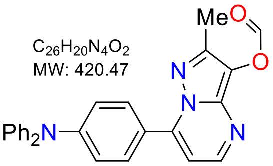

4-(Diphenylamino)phenyl formate ***2j****.* This ester was obtained (in 6 h) as a grey solid (143 mg, 99%) from 4-diphenylaminobenzaldehyde (**1j**, 136 mg, 0.5 mmol). Mp 106–108 °C. ^{1}H NMR (400 MHz, $CDCl_3$): δ = 7.02 (m, 4H), 7.08 (m, 6H), 7.25 (m, 4H), 8.30 (s, 1H) ppm. ^{13}C NMR (101 MHz, $CDCl_3$): δ = 121.8 (CH), 123.1 (CH), 124.3 (CH), 124.7 (CH), 129.4 (CH), 144.8 (C), 146.1 (C), 147.6 (C), 159.6 (CH) ppm. HRMS (ESI+): calcd. for $C_{19}H_{16}NO_2^+$ 290.1175 [M + H]$^+$; found 290.1161.

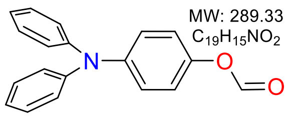

Anthracen-9-yl formate ***2k***. By the general method for 4 h from 9-formylanthracene (**1l**, 103 mg, 0.5 mmol), **2k** was obtained as a white solid (106 mg, 96%). Mp 97–98 °C (Lit.[10] 90–91 °C). ^{1}H NMR (400 MHz, $CDCl_3$): δ = 7.53 (m, 4H), 8.03 (m, 4H), 8.42 (s, 1H), 8.68 (s, 1H) ppm. ^{13}C NMR (101 MHz, $CDCl_3$): δ = 121.1 (CH), 123.8 (C), 125.4 (CH), 125.8 (CH), 126.8 (CH), 128.4 (CH), 131.8 (C), 141.0 (C), 159.9 (CH) ppm. HRMS (ESI+): calcd. for $C_{15}H_{11}O_2^+$ 223.0754 $[M + H]^+$; found 223.0759. These data matched with the previously reported results.[10]

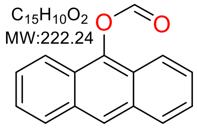

Pyren-1-yl formate ***2l***. By the general method for 14 h from 1-formypyrene (**1l**, 115 mg, 0.5 mmol), **2l** was obtained as a yellow solid (117 mg, 95%). Mp 85–86 °C. ^{1}H NMR (400 MHz, $CDCl_3$): δ = 7.79 (d, *J* = 8.3 Hz, 1H), 8.01 (m, 3H), 8.10–8.19 (m, 5H), 8.64 (s, 1H) ppm. ^{13}C NMR (101 MHz, $CDCl_3$): δ = 119.0 (CH), 119.9 (CH), 122.9 (C), 124.3 (C), 125.0 (CH), 125.4 (CH), 125.5 (C), 125.7 (CH), 126.5 (CH), 126.9 (CH), 127.4 (CH), 128.5 (CH), 129.6 (C), 130.8 (C), 131.0 (C), 143.2 (C), 159.8 (CH) ppm. HRMS (ESI+): calcd. for $C_{17}H_{11}O_2^+$ 247.0754 $[M + H]^+$; found 247.0754.

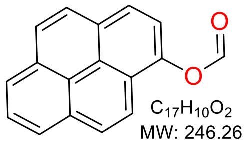

7-Diethylaminocoumarin-3-yl acetate ***2n***. Following the general procedure for 14 h from the ketone **1n** (130 mg, 0.5 mmol), **2n** was obtained as a green solid (132 mg, 96%). Mp 129–130 °C. ^{1}H NMR (400 MHz, $CDCl_3$): δ = 1.20 (t, *J* = 6.8 Hz, 6H), 2.32 (s, 3H), 3.40 (q, *J* = 6.7 Hz, 4H), 6.51 (s, 1H), 6.60 (d, *J* = 8.5 Hz, 1H), 7.21–7.35 (m, 2H). ^{13}C NMR (101 MHz, $CDCl_3$): δ = 12.4 (CH_3), 20.6 (CH_3), 44.8 (CH_2), 97.5 (CH), 107.0 (C), 109.3 (CH), 128.7 (CH), 131.0 (C), 132.0 (CH), 150.4 (C), 154.8 (C), 157.8 (C), 168.8 (C) ppm. HRMS (ESI+): calcd. for $C_{15}H_{18}NO_4^+$ 276.1230 $[M + H]^+$; found 276.1234.

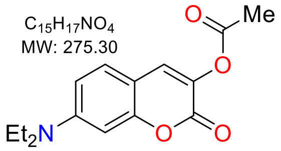

2-Methyl-7-phenylpyrazolo[1,5-a]pyrimidin-3-yl acetate ***2o***. By the general procedure for 6 h from 3-acetyl-2-methyl-7-phenylpyrazolo[1,5-*a*]pyrimidine (**1o**, 126 mg, 0.5 mmol), **2o** was obtained as a yellow solid (131 mg, 98%). Mp 129–130 °C (amorphous). ^{1}H NMR (400 MHz, $CDCl_3$): δ = 2.41/244 (s/s, 6H), 6.80 (d, *J* = 4.3 Hz, 1H), 7.56 (m, 3H), 8.02 (m, 2H), 8.44 (d, *J* = 4.3 Hz, 1H) ppm. ^{13}C NMR (101 MHz, $CDCl_3$): δ = 11.9 (CH_3), 20.6 (CH_3), 107.0 (CH), 121.7 (C), 128.8 (CH), 129.3 (CH), 130.5 (C), 131.2 (CH), 140.8 (C), 146.3 (C), 146.7 (C), 148.8 (CH), 169.1 (C) ppm. HRMS (ESI+): calcd. for $C_{15}H_{14}N_3O_2^+$ 268.1080 $[M + H]^+$; found 268.1080.

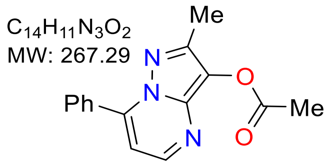

2-Methyl-7-phenylpyrazolo[1,5-a]pyrimidin-3-ol ***3a***. This alcohol was obtained as a red solid (53 mg, 95%) via the general method from 2-methyl-7-phenylpyrazolo[1,5-*a*]pyrimidin-3-yl formate (**2a**, 64 mg, 0.25 mmol). Mp 173–174 °C. ^{1}H NMR (400 MHz, $CDCl_3$): δ = 2.54 (s, 3H), 6.65 (s-br, 1H), 7.55 (m, 3H), 8.04 (m, 2H), 8.27 (s-br, 1H), 9.20 (s-br, 1H) ppm. ^{13}C NMR (101 MHz, $DCDCl_3$) δ = 11.4 (CH_3), 105.2 (CH), 128.2 (C), 128.7 (CH), 129.2 (CH), 130.7 (C), 131.2 (CH), 136.8 (C), 143.7 (C), 145.8

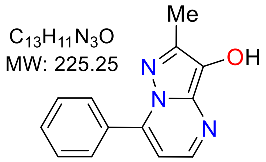

(CH), 146.5 (C) ppm; HRMS (ESI) m/z calcd for $C_{13}H_{12}N_3O_2^+$ [M + H]$^+$ 226.0975, found 226.0979. These data matched with previously reported data by us.[9]

7-(4-Chlorophenyl)-2-methylpyrazolo[1,5-a]pyrimidin-3-ol ***3b***. By the general procedure from 7-(4-

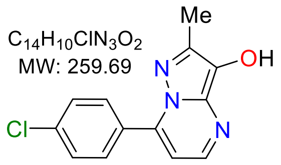

chlorophenyl)-2-methylpyrazolo[1,5-*a*]pyrimidin-3-yl formate (**2b**, 72 mg, 0.25 mmol), **3b** was obtained as a red solid (60 mg, 92%). Mp 200–201 °C. ^{1}H NMR (400 MHz, DMSO-d_6): δ = 2.33 (s, 3H), 6.94 (d, *J* = 4.2 Hz, 2H), 7.64 (d, *J* = 8.5 Hz, 2H), 8.12 (d, *J* = 8.5 Hz, 2H), 8.32 (d, *J* = 4.2 Hz, 2H), 8.86 (s-br, 1H) ppm. ^{13}C NMR (101 MHz, DMSO-d_6): δ = 11.23 (CH_3), 105.9 (CH), 128.1 (C), 128.6 (CH), 129.5 (C), 131.0 (CH), 135.4 (C), 137.8 (C), 142.2 (C), 142.9 (C), 146.0 (CH) ppm. HRMS (ESI+): calcd. for $C_{13}H_{11}{}^{35}ClN_3O^+$ 260.0585 [M + H]$^+$; found 260.0587.

7-(4-Bromophenyl)-2-methylpyrazolo[1,5-a]pyrimidin-3-ol ***3c***. Following the general procedure

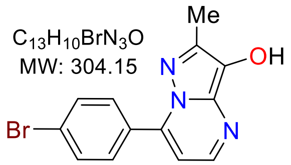

from 7-(4-bromophenyl)-3-formyl-2-methylpyrazolo[1,5-*a*]pyrimidin-3-yl formate (**2c**, 83 mg, 0.25 mmol), **3c** was obtained as a red solid (73 mg, 96%). Mp 196–197 °C. ^{1}H NMR (400 MHz, DMSO-d_6): δ = 2.33 (s, 3H), 6.94 (d, *J* = 4.2 Hz, 1H), 7.78 (d, *J* = 8.6 Hz, 2H), 8.04 (d, *J* = 8.6 Hz, 2H), 8.32 (d, *J* = 4.2 Hz, 1H), 8.85 (s-br, 1H) ppm. ^{13}C NMR (101 MHz, DMSO-d_6): δ = 11.8 (CH_3), 107.0 (CH), 120.8 (C), 122.9 (C), 129.1 (CH), 130.8 (CH), 140.6 (C), 145.2 (C), 146.5 (C), 149.0 (CH), 158.7 (C) ppm. HRMS (ESI+): calcd. for $C_{13}H_{11}{}^{79}BrN_3O^+$ 304.0080 [M + H]$^+$; found 304.0080.

*7-(4-Methoxyphenyl)-2-methylpyrazolo[1,5-a]pyrimidin-3-ol 3**d***. Following the general procedure

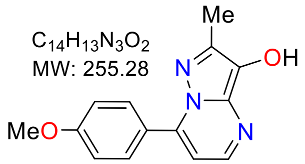

and from 7-(4-methoxyphenyl)-2-methylpyrazolo[1,5-*a*]pyrimidine-3-yl formate (**2d**, 71 mg, 0.25 mmol), **3d** was obtained as a red solid (62 mg, 97%). Mp 214–215 °C. ^{1}H NMR (400 MHz, DMSO-d_6): δ = 2.34 (s, 3H), 3.85 (s, 3H), 6.89 (d, *J* = 4.1 Hz, 1H), 7.12 (d, *J* = 8.7 Hz, 2H), 8.12 (d, *J* = 8.7 Hz, 2H), 8.27 (d, *J* = 4.1 Hz, 1H), 8.76 (s-br, 1H) ppm. ^{13}C NMR (101 MHz, DMSO-d_6): δ = 11.3 (CH_3), 55.4 (CH_3), 105.0 (CH), 113.9 (CH), 122.7 (C), 127.8 (C), 130.9 (CH), 138.1 (C), 142.0 (C), 143.9 (C), 146.0 (CH), 161.2 (C) ppm. HRMS (ESI+): calcd. for $C_{14}H_{14}N_3O_2^+$ 256.1081 [M + H]$^+$; found 256.1087.

2-Methyl-7-(4-nitrophenyl)pyrazolo[1,5-a]pyrimidin-3-ol ***3e***. This alcohol was obtained as a red

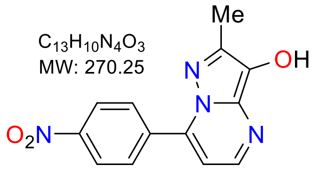

solid (63 mg, 93%) by the general procedure from 7-(4-nitrophenyl)-2-methylpyrazolo[1,5-*a*]pyrimidin-3-yl formate (**2e**, 75 mg, 0.25 mmol), Mp 236–237 °C. ^{1}H NMR (400 MHz, DMSO-d_6): δ = 2.33 (s, 3H), 7.02 (d, *J* = 4.2 Hz, 1H), 8.37 (m, 5H), 8.9 (s-br, 1H) ppm. ^{13}C NMR (101 MHz, DMSO-d_6): δ = 11.2 (CH_3), 106.8 (CH), 123.4 (CH), 128.4 (C), 130.6 (CH), 136.8 (C), 137.8 (C), 141.9 (C), 142.3 (C), 146.0 (CH), 148.3 (C) ppm. HRMS (ESI+): calcd. for $C_{13}H_{11}N_4O_3^+$ 271.0826 [M + H]$^+$; found 271.0825.

*7-(4-Cyanophenyl)-2-methylpyrazolo[1,5-a]pyrimidin-3-ol **3f***. By the general method and using the crude ester obtained from 7-(4-cyanophenyl)-2-methylpyrazolo[1,5-*a*]pyrimidine-3-carbaldehyde (**1f**, 131 mg, 0.5 mmol), **3f** was obtained as a red solid (110 mg, 88%). Mp 244–246 °C. ^{1}H NMR (400 MHz, DMSO-d_6): δ = 2.33 (s, 3H), 7.01 (d, *J* = 4.2 Hz, 1H), 8.05 (d, *J* = 8.0 Hz, 2H), 8.26 (d, *J* = 8.0 Hz, 2H), 8.36 (d, *J* = 4.3 Hz, 1H), 8.92 (s-br, 1H) ppm. ^{13}C NMR (101 MHz, DMSO-d_6): δ = 11.2 (CH_3), 106.6 (CH), 113.0 (C), 118.4 (C), 128.3 (C), 130.0 (CH), 132.4 (CH), 135.1 (C), 137.8 (C), 142.2 (C), 142.3 (C), 146.0 (CH) ppm. HRMS (ESI+): calcd. for $C_{14}H_{11}N_4O^+$ 251.0927 [M + H]$^+$; found 251.0915.

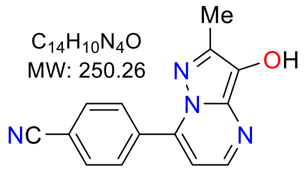

*2-Methyl-7-(4-pyridyl)pyrazolo[1,5-a]pyrimidin-3-ol **3g***. By the general method from 2-methyl-7-(4-pyridyl)pyrazolo[1,5-*a*]pyrimidin-3-yl formate (**2g**, 64 mg, 0.25 mmol), **3g** was obtained as a red solid (49 mg, 86%). Mp 228–230 °C. ^{1}H NMR (400 MHz, DMSO-d_6): δ = 2.34 (s, 3H), 7.08 (d, *J* = 4.1 Hz, 2H), 8.08 (d, *J* = 5.2 Hz, 2H), 8.38 (d, *J* = 4.1 Hz, 2H), 8.80 (d, *J* = 4.8 Hz, 2H), 8.95 (s-br, 1H) ppm. ^{13}C NMR (101 MHz, DMSO-d_6): δ = 11.2 (CH_3), 106.5 (CH), 123.1 (CH), 128.4 (C), 137.8 (C), 138.1 (C), 141.5 (C), 142.4 (C), 146.0 (CH), 150.1 (CH) ppm. HRMS (ESI+): calcd. for $C_{12}H_{11}N_4O^+$ 227.0927 [M + H]$^+$; found 227.0928.

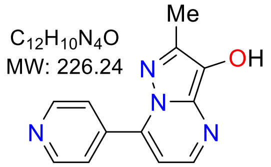

*2-Methyl-7-(2-pyridyl)pyrazolo[1,5-a]pyrimidin-3-ol **3h***. By the general method from 2-methyl-7-(2-pyridyl)pyrazolo[1,5-*a*]pyrimidin-3-yl formate (**2h**, 64 mg, 0.25 mmol), **3h** was obtained as a red solid (47 mg, 83%). Mp 246–247 °C. ^{1}H NMR (400 MHz, DMSO-d_6): δ = 2.39 (s, 3H), 7.47 (s-br, 1H), 7.60 (m-br, 1H), 8.07 (t-br, 1H), 8.42 (s-br, 1H), 8.92 (m, 3H) ppm. ^{13}C NMR (101 MHz, DMSO-d_6): δ = 11.3 (CH_3), 106.1 (CH), 125.5 (CH), 125.7 (CH), 128.3 (C), 137.0 (CH), 138.4 (C), 141.8 (C), 142.0 (C), 145.8 (CH), 147.8 (C), 150.1 (CH) ppm. HRMS (ESI+): calcd. for $C_{12}H_{11}N_4O^+$ 227.0927 [M + H]$^+$; found 227.0928.

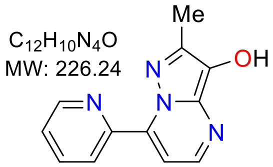

*7-(4-Diphenylaminophenyl)-2-methylpyrazolo[1,5-a]pyrimidin-3-ol **3i***. This alcohol was obtained as a red solid (93 mg, 95%) by the general protocol from 3-formyl-7-(4-diphenylaminophenyl)-2-methylpyrazolo[1,5-*a*]pyrimidin-3-yl formate (**2i**, 105 mg, 0.25 mmol). Mp 226–227 °C. ^{1}H NMR (400 MHz, DMSO-d_6): δ = 2.53 (s, 3H), 6.63 (s, 1H), 7.14 (m, 8H), 7.31 (t, *J* = 7.8 Hz, 4H), 8.01 (d, *J* = 8.8 Hz, 2H), 8.21 (s, 1H), 9.38 (s-br, 1H) ppm. ^{13}C NMR (101 MHz, DMSO-d_6): δ = 11.4 (CH_3), 104.1 (CH), 120.6 (CH), 122.6 (C), 124.3 (CH), 125.8 (CH), 128.0 (C), 129.6 (CH), 130.3 (CH), 137.1 (C), 143.4 (C), 145.6 (CH), 146.1 (C), 146.8 (C) ppm. HRMS (ESI+): calcd. for $C_{25}H_{21}N_4O^+$ 393.1710 [M + H]$^+$; found 393.1701.

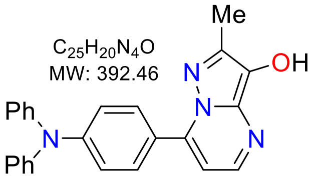

4-(Diphenylamino)phenol ***3j***. This alcohol was obtained as a grey solid (69 mg, 95%) by the general procedure from 4-diphenylaminophenyl formate (**1j**, 72 mg, 0.25 mmol). Mp 127–128 °C (Lit.[11] 121 °C). ^{1}H NMR (400 MHz, $CDCl_3$): δ = 4.95 (s-br, 1H), 6.79 (m, 2H), 6.97–7.05 (m, 8H), 7.23 (m, 4H) ppm. ^{13}C NMR (101 MHz, $CDCl_3$): δ = 116.3 (CH), 121.9 (CH), 122.9 (CH), 127.5 (CH), 129.1 (CH), 140.9 (C), 148.1 (C), 151.9 (C) ppm. These data matched with the previously reported results.[11]

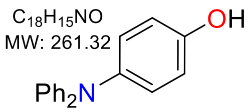

Anthracen-9-ol → anthracen-9(10H)-one ***3k***. By the general protocol from 9-anthracenyl formate (**1k**, 56 mg, 0.25 mmol), **3k** was obtained as a red solid (44 mg, 90%). Mp 182–183 °C (Lit.[12] 155–158 °C). ^{1}H NMR (400 MHz, $CDCl_3$): δ = 4.34 (s, 2H), 7.46 (m, 4H), 7.58 (m, 2H), 8.35 (d, J = 7.5 Hz, 2H) ppm. ^{13}C NMR (100 MHz, $CDCl_3$): δ = 32.3 (CH_2), 127.0 (CH), 127.6 (CH), 128.5 (CH), 132.0 (C), 132.8 (CH), 140.5 (C), 184.3 (C) ppm. These data matched with the previously reported.[12]

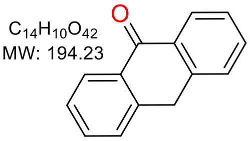

Pyren-1-ol ***3l*** was obtained as a red solid (51 mg, 93%) by the general protocol from the ester **2l** (62 mg, 0.25 mmol). Mp 176–178 °C (Lit.[13] 169–171). ^{1}H NMR (400 MHz, $CDCl_3$): δ = 7.59 (d, J = 8.3 Hz, 1H), 7.89 (d, J = 8.9 Hz, 1H), 7.95–8.05 (m, 3H), 8.12 (t, J = 7.1 Hz, 3H), 8.33 (d, J = 9.2 Hz, 1H), 10.62 (s-br, 1H) ppm. ^{13}C NMR (101 MHz, $CDCl_3$): δ = 113.2 (CH), 118.1 (C), 121.4 (CH), 123.5 (CH), 123.6 (C), 123.8 (CH), 123.9 (CH), 124.4 (C), 125.4 (C), 125.5 (CH), 126.1 (CH), 126.2 (CH), 127.4 (CH), 131.3 (C), 131.4 (C), 152.1 (C) ppm. These data matched with the reported.[13]

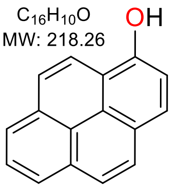

9H-Fluoren-2-ol ***3m***. By the general method and using the crude ester obtained from 2-formyl-9*H*-fluorene (**1m**, 97 mg, 0.5 mmol), **3m** was obtained as a yellow solid (76 mg, 83%). Mp 166–168 °C (Lit.[14] 168–169 °C). ^{1}H NMR (400 MHz, $CDCl_3$): δ = 3.81 (s, 2H), 6.79 (d, J = 8.1 Hz, 1H), 6.98 (s, 1H), 7.18 (t, J = 7.3 Hz, 1H), 7.30 (t, J = 7.4 Hz, 1H), 7.48 (d, J = 7.4 Hz, 1H), 7.66 (d, J = 8.2 Hz, 1H), 7.70 (d, J = 7.5 Hz, 1H), 9.49 (s-br, 1H) ppm. ^{13}C{^{1}H} NMR (100 MHz, $CDCl_3$): δ = 36.3 (CH_2), 112.1 (CH), 114.1 (CH), 118.7 (CH), 120.8 (CH), 124.9 (CH), 125.1 (CH), 126.6 (CH), 132.4 (C), 141.5 (C), 142.2 (C), 144.9 (C), 157.1 (C) ppm. These data matched with the previous reports.[14]

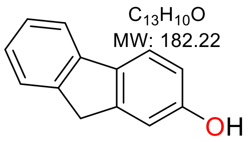

7-Diethylaminocoumarin-3-ol ***3n***. By the general procedure from 7-diethylaminocoumarin-3-yl acetate (**2n**, 69 mg, 0.25 mmol), **3n** was obtained as a red solid (53 mg, 91%). Mp 99–100 °C. ^{1}H NMR (400 MHz, DMSO-d_6): δ = 1.09 (t, J = 6.4 Hz, 6H), 3.36 (d-br, J = 6.5 Hz, 4H), 6.50 (s, 1H), 6.64 (d, J = 8.5 Hz, 1H), 6.98 (s, 1H), 7.26 (d, J = 8.5 Hz, 1H) ppm. ^{13}C NMR (101 MHz, $CDCl_3$): δ = 12.3 (CH_3), 43.9 (CH_2), 97.0 (CH), 108.6 (CH), 109.2 (CH), 116.7 (CH), 126.9 (C), 137.4 (C), 147.5 (C), 151.4 (C), 159.2 (C) ppm. HRMS (ESI+): calcd. for $C_{13}H_{16}NO_3^+$ 234.1125 $[M + H]^+$; found 234.1126.

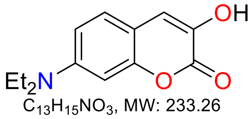

4-((4-hydroxyphenyl)(phenyl)amino)benzaldehyde ***3w***. This hydroxyaldehyde was obtained as a pale-yellow solid (64 mg, 44%) by the general procedure from 4,4'-(phenylazanediyl)dibenzaldehyde (**1w**, 151 mg, 0.5 mmol). Mp 138–139 °C (Lit.[15] 141 °C). ^{1}H NMR (400 MHz, $CDCl_3$): δ = 6.66 (s-br, 1H), 6.87 (d, *J* = 8.7 Hz 2H), 6.93 (d, *J* = 8.7 Hz 2H), 7.06 (d, *J* = 8.7 Hz 2H), 6.87 (d, *J* = 8.7 Hz 2H), 7.12–7.18 (m, 3H), 7.33 (t, *J* = 7.8 Hz 2H), 7.65 (d, *J* = 8.7 Hz 2H), 9.76 (s, 1H) ppm. ^{13}C NMR (101 MHz, $CDCl_3$): δ = 116.8 (CH), 117.9 (CH), 124.9 (CH), 125.9 (CH), 128.2 (C), 128.7 (CH), 129.7 (CH), 131.5 (CH), 138.3 (C), 146.1 (C), 153.9 (C), 154.4 (C), 190.7 (C) ppm. HRMS (ESI+): calcd. for $C_{19}H_{16}NO_2^+$ 290.1176 [M + H]$^+$; found 290.1168.

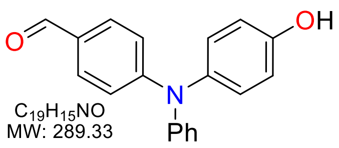

4-(Diphenylamino)phenol ***3w'***. This alcohol was obtained as a white solid (15 mg, 11%) by the general procedure from **1w** (151 mg, 0.5 mmol). Mp 217–218 °C (Lit.[16] 220-222 °C). ^{1}H NMR (400 MHz, $CDCl_3$): δ = 6.78 –7.10 (m, 13H), 8.17 (s, 2H, OH) ppm. ^{13}C NMR (101 MHz, $CDCl_3$): δ = 116.1 (CH), 119.7 (CH), 119.9 (CH), 126.7 (CH), 128.6 (CH), 140.1 (C), 149.6 (C), 152.8 (C) ppm. HRMS (ESI+): calcd. for $C_{18}H_{16}NO_2^+$ 278.1175 [M + H]$^+$; found 278.1159.

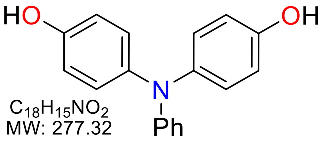

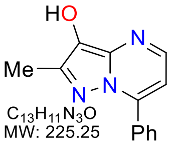

2-Methyl-7-phenylpyrazolo[1,5-a]pyrimidin-3-ol ***3o*** = ***3a***. By the general procedure from 2-methyl-7-phenylpyrazolo[1,5-*a*]pyrimidin-3-yl acetate (**2o**, 67 mg, 0.25 mmol), **3o** was obtained as a red-orange solid (56 mg, 99%). This compound was also obtained from de respective formyl ester **2a** (see characterization data above).[9]

3-Methoxy-2-methyl-7-phenylpyrazolo[1,5-a]pyrimidine ***5a***. By the general procedure from **3a** (113 mg, 0.5 mmol) and iodomethane (**4a**, 70 mg, 0.75 mmol), **5a** was obtained as a yellow solid (110 mg, 92%). Mp 96–97 °C. ^{1}H NMR (400 MHz, $CDCl_3$): δ = 2.47 (s, 3H), 4.12 (s, 3H), 6.69 (d, *J* = 4.2 Hz, 1H), 7.54 (m, 3H), 8.02 (m, 2H), 8.34 (d, *J* = 4.2 Hz, 1H) ppm. ^{13}C NMR (101 MHz, $CDCl_3$): δ = 11.6 (CH_3), 61.7 (CH_3), 106.2 (CH), 128.7 (CH), 129.2 (CH), 130.9 (C), 131.0 (CH), 131.6 (C), 139.9 (C), 145.3 (C), 145.8 (C), 146.9 (CH), ppm. HRMS (ESI+): calcd. for $C_{14}H_{14}N_3O^+$ 240.1131 [M + H]$^+$; found 240.1134.

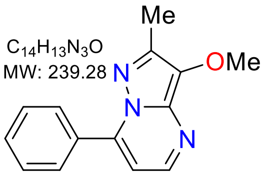

7-(4-Chlorophenyl)-3-methoxy-2-methylpyrazolo[1,5-a]pyrimidine ***5b***. By the general method from **3b** (130 mg, 0.5 mmol) and iodomethane (**4a**, 70 mg, 0.75 mmol), **4b** was obtained as a yellow solid (132 mg, 96%). Mp 134–136 °C. ^{1}H NMR (400 MHz, $CDCl_3$): δ = 2.47 (s, 3H), 4.12 (s, 3H), 6.68 (d, *J* = 3.8 Hz, 1H), 7.52 (d, *J* = 8.3 Hz, 2H), 7.99 (d, *J* = 8.3 Hz, 2H), 8.33 (d, *J* = 3.8 Hz, 1H) ppm. ^{13}C NMR (101 MHz, $CDCl_3$): δ = 11.5 (CH_3), 61.7 (CH_3), 105.9 (CH), 128.9 (CH), 129.2 (C), 130.5 (CH), 131.7 (C), 137.0 (C), 139.8 (C), 144.5 (C), 145.3 (C), 146.7 (CH), ppm. HRMS (ESI+): calcd. for $C_{14}H_{13}{}^{35}ClN_3O^+$ 274.0742 [M + H]$^+$; found 274.0736.

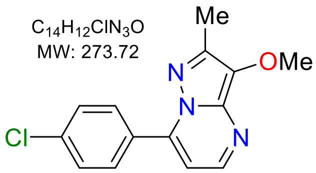

3-Methoxy-2-ethyl-7-phenylpyrazolo[1,5-a]pyrimidine ***5c***. By the general procedure from **3a** (113 mg, 0.5 mmol) and iodoethane (**4b**, 117 mg, 0.75 mmol), **5c** was obtained as a yellow solid (60 mg, 95%). Mp 100–101 °C. ^{1}H NMR (400 MHz, $CDCl_3$): δ = 1.42 (t, *J* = 7.0 Hz, 3H), 2.46 (s, 3H), 4.36 (q, *J* = 7.0 Hz, 2H), 6.70 (d, *J* = 4.2 Hz, 1H), 7.54 (m, 3H), 8.02 (m, 2H), 8.34 (d, *J* = 4.2 Hz, 1H) ppm. ^{13}C NMR (101 MHz, $CDCl_3$): δ = 11.6 (CH_3), 15.6 (CH_3), 69.8 (CH_2), 106.2 (CH), 128.7 (CH), 129.2 (CH), 130.3 (C), 130.9 (C), 131.0 (CH), 140.2 (C), 145.8 (C), 146.0 (C), 146.9 (CH) ppm. HRMS (ESI+): calcd. for $C_{15}H_{16}N_3O^+$ 254.1288 $[M + H]^+$; found 254.1300.

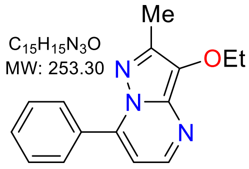

7-(4-Chlorophenyl)-3-ethoxy-2-methylpyrazolo[1,5-a]pyrimidine ***5d***. By the general procedure from **3b** (130 mg, 0.5 mmol) and iodoethane (**4b**, 117 mg, 0.75 mmol), the ether **5d** was obtained as a yellow solid (137 mg, 95%). Mp 99–100 °C. ^{1}H NMR (400 MHz, $CDCl_3$): δ = 1.42 (t, *J* = 7.0 Hz, 3H), 2.46 (s, 3H), 4.36 (q, *J* = 7.0 Hz, 2H), 6.68 (d, *J* = 4.2 Hz, 2H), 7.52 (d, *J* = 8.5 Hz, 2H), 7.99 (d, *J* = 8.5 Hz, 2H), 8.33 (d, *J* = 4.2 Hz, 2H) ppm. ^{13}C NMR (101 MHz, $CDCl_3$): δ = 11.6 (CH_3), 15.6 (CH_3), 69.8 (CH_2), 106.0 (CH), 129.0 (CH), 129.3 (C), 130.4 (C), 130.6 (CH), 137.0 (C), 140.1 (C), 144.6 (C), 146.1 (C), 146.8 (CH) ppm. HRMS (ESI+): calcd. for $C_{15}H_{15}{}^{35}ClN_3O^+$ 288.0898 $[M + H]^+$; found 288.0897.

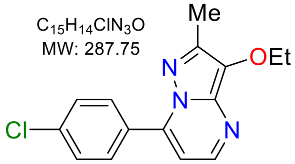

3-Benzyloxy-7-(4-chlorophenyl)-2-methylpyrazolo[1,5-a]pyrimidine ***5e***. By the general procedure from **3b** (130 mg, 0.5 mmol) and benzyl bromide (**4c**, 115 mg, 0.75 mmol), **5e** was obtained as a yellow solid (113 mg, 95%). Mp 99–100 °C. ^{1}H NMR (400 MHz, $CDCl_3$): δ = 2.31 (s, 3H), 5.39 (s, 2H), 6.70 (d, *J* = 4.2 Hz, 1H), 7.30–7.37 (m, 3H), 7.46 (d, *J* = 6.7 Hz, 2H), 7.52 (d, *J* = 8.6 Hz, 2H), 7.99 (d, *J* = 8.6 Hz, 2H), 8.38 (d, *J* = 4.2 Hz, 1H) ppm. ^{13}C NMR (101 MHz, $CDCl_3$): δ = 11.5 (CH_3), 75.5 (CH_2), 106.0 (CH), 128.1 (CH), 128.4 (CH), 128.6 (CH), 129.0 (CH), 129.2 (C), 130.1 (C), 130.6 (CH), 137.1 (C), 137.6 (C), 140.1 (C), 144.6 (C), 146.2 (C), 146.8 (CH) ppm. HRMS (ESI+): calcd. for $C_{20}H_{17}{}^{35}ClN_3O^+$ 350.1055 $[M + H]^+$; found 350.1063.

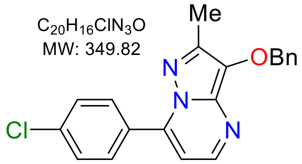

7-(4-Chlorophenyl)-3-(4-(8-quinolinoxy)butoxy)-2-methylpyrazolo[1,5-a]pyrimidine ***5f:*** By the general method from **3b** (130 mg) and 8-(4-bromobutoxy)quinoline (**4d**, 210 mg, 0.75 mmol), **37f** was obtained as a red oil (204 mg, 89%). ^{1}H NMR (400 MHz, $CDCl_3$): δ = 2.07 (m, 2H), 2.27 (m, 2H), 2.44 (s, 3H), 4.34–4.43 (m, 4H), 6.63 (d, *J* = 4.1 Hz, 1H), 7.10 (d, *J* = 7.5 Hz, 1H), 7.32–7.45 (m, 4H), 7.50 (d, *J* = 8.5 Hz, 2H), 7.97 (d, *J* = 8.5 Hz, 1H), 8.10 (d, *J* = 9.2 Hz, 1H), 8.30 (d, *J* = 4.1 Hz, 1H), 8.92 (d, *J* = 4.1 Hz, 1H) ppm. ^{13}C NMR (101 MHz, $CDCl_3$): δ = 11.6 (CH_3), 25,6 (CH_2), 26,7 (CH_2), 68.6 (CH_2), 73.6 (CH_2), 105.9 (CH), 108.7 (CH), 119.4 (CH), 121.5 (CH), 126.7 (CH), 128.9 (CH), 129.2 (C), 129.5 (C), 130.5 (CH), 130.6 (C), 135.8 (CH), 137.0 (C), 139.9 (C), 140.4 (C), 144.4 (C), 145.7 (C), 146.7 (CH), 149.2 (CH), 154.7 (C) ppm. HRMS (ESI+): calcd. for $C_{26}H_{24}ClN_4O_2^+$ 459.1582 $[M + H]^+$; found 459.1577.

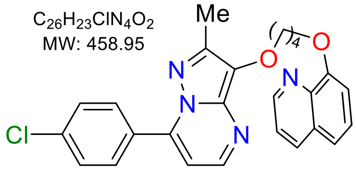

7-(4-Chlorophenyl)-2-methylpyrazolo[1,5-a]pyrimidin-3-yl 4-bromobenzoate ***5g***. By the general method from **3b** (130 mg) and 4-bromobenzoyl chloride (**4e** 98%, 157 mg/0.75 mmol), **5g** was obtained as a yellow solid (203 mg, 92%). Mp 208–209 °C. ^{1}H NMR (400 MHz, $CDCl_3$): δ = 2.45 (s, 3H), 6.82 (d, *J* = 4.3 Hz, 1H), 7.55 (d, *J* = 8.6 Hz, 2H), 7.68 (d, *J* = 8.5 Hz, 2H), 8.02 (d, *J* = 8.6 Hz, 2H), 8.16 (d, *J* = 8.5 Hz, 2H), 8.46 (d, *J* = 4.3 Hz, 1H) ppm. ^{13}C NMR (101 MHz, $CDCl_3$): δ = 11.9 (CH_3), 106.9 (CH), 121.8 (C), 127.6 (C), 128.8 (C), 129.1 (CH), 129.2 (C), 130.7 (CH), 132.0 (CH), 137.4 (C), 140.9 (C), 145.1 (C), 146.9 (C), 148.8 (C), 164.0 (C) ppm. HRMS (ESI+): calcd. for $C_{20}H_{14}BrClN_3O_2^+$ 441.9952 [M + H]$^+$; found 441.9953.

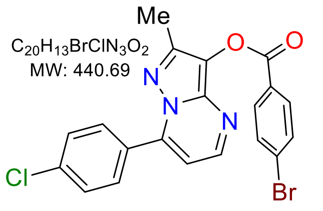

7-(4-Chlorophenyl)-2-methylpyrazolo[1,5-a]pyrimidin-3-yl benzenesulfonate ***5h:*** By the general procedure from **3b** (130 mg, 0.5 mmol) and benzenesulfonyl chloride (**4f** 99%, 97 μL g/0.75 mmol), **5h** was obtained as a white solid (182 mg, 91%). Mp 190–191 °C. ^{1}H NMR (400 MHz, $CDCl_3$): δ = 2.25 (s, 3H), 6.80 (d, *J* = 4.2 Hz, 1H), 7.54 (m, 4H), 7.69 (t, *J* = 7.5 Hz, 1H), 7.97 (m, 4H), 8.36 (d, *J* = 4.2 Hz, 1H) ppm. ^{13}C NMR (100 MHz, $CDCl_3$): δ = 11.5 (CH_3), 107.4 (CH), 120.5 (C), 128.4 (C), 128.9 (CH), 129.1 (CH), 129.2 (CH), 130.7 (CH), 134.4 (CH), 135.4 (C), 137.6 (C), 141.8 (C), 145.0 (C), 148.0 (C), 149.5, ppm. HRMS (ESI+): calcd. for $C_{19}H_{15}ClN_3O_3S^+$ 400.0517 [M + H]$^+$; found 400.0527.

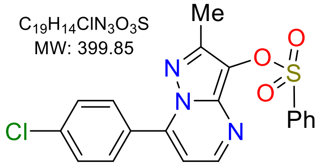

4. HRMS analysis

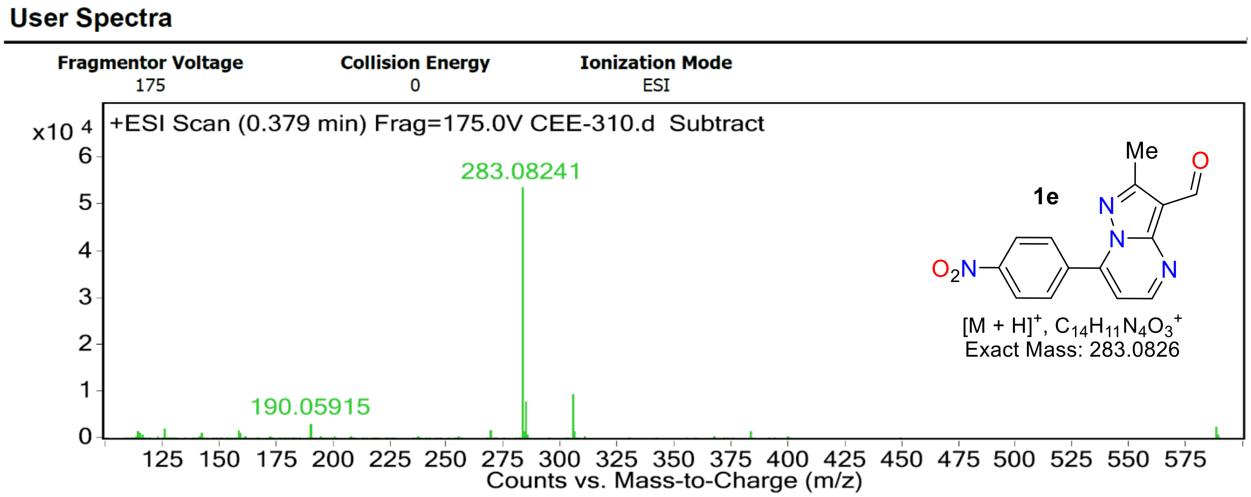

Peak List

m/z	z	Abund	Abund %
190.05915		3295.3	6.13
283.08241	1	53727	100
283.18546		2724.4	5.07
284.08525	1	8026.6	14.94
305.06413		9675.6	18.01

Fig. S1 HRMS analysis of 2-methyl-7-(4-nitrophenyl)pyrazolo[1,5-*a*]pyrimidine-3-carbaldehyde (**1e**).

User Spectra

Fragmentor Voltage	Collision Energy	Ionization Mode
175	0	ESI

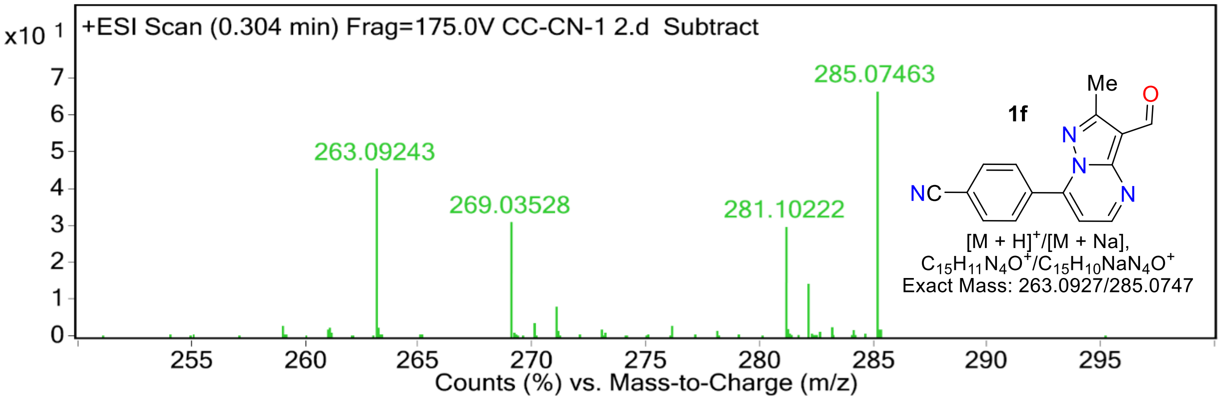

Peak List

m/z	z	Abund	Abund %
112.01878	2	55114.8	100
184.983		30381.1	55.12
201.03441	2	15882.4	28.82
263.09243	1	25175.7	45.68
269.03528		17193.9	31.2
281.10222		16535.9	30
285.07463	1	36852	66.86

Fig. S2 HRMS analysis of 7-(4-cyanophenyl)-3-formyl-2-methylpyrazolo[1,5-*a*]pyrimidine (**1f**)

User Spectra

Fragmentor Voltage	Collision Energy	Ionization Mode
175	0	ESI

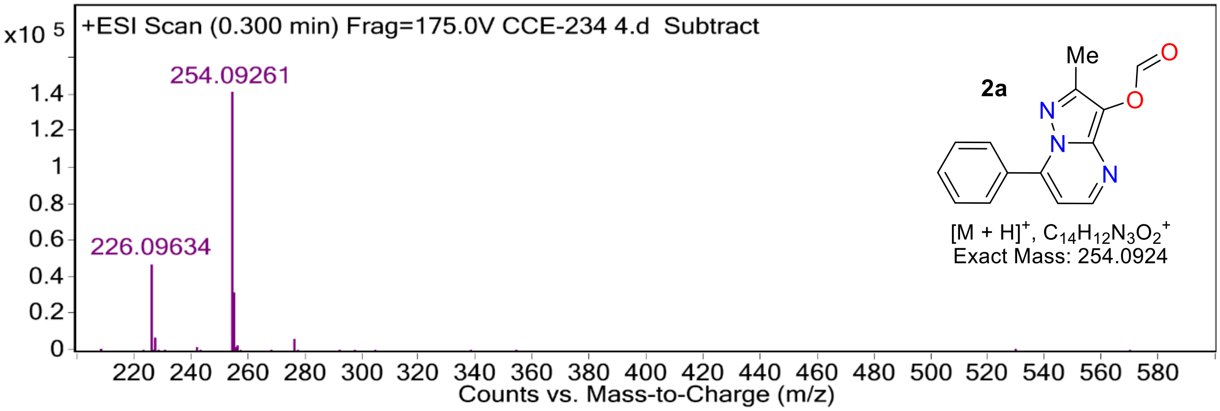

Peak List

m/z	z	Abund	Abund %
157.07501		9186.7	6.49
226.09634	1	47828.9	33.77
227.09959	1	7887.8	5.57
254.09261	1	141643.3	100
255.09359	1	32539.4	22.97

Fig. S3 HRMS analysis of 2-methyl-7-phenylpyrazolo[1,5-*a*]pyrimidin-3-yl formate (**2a**)

User Spectra

Fragmentor Voltage	Collision Energy	Ionization Mode
175	0	ESI

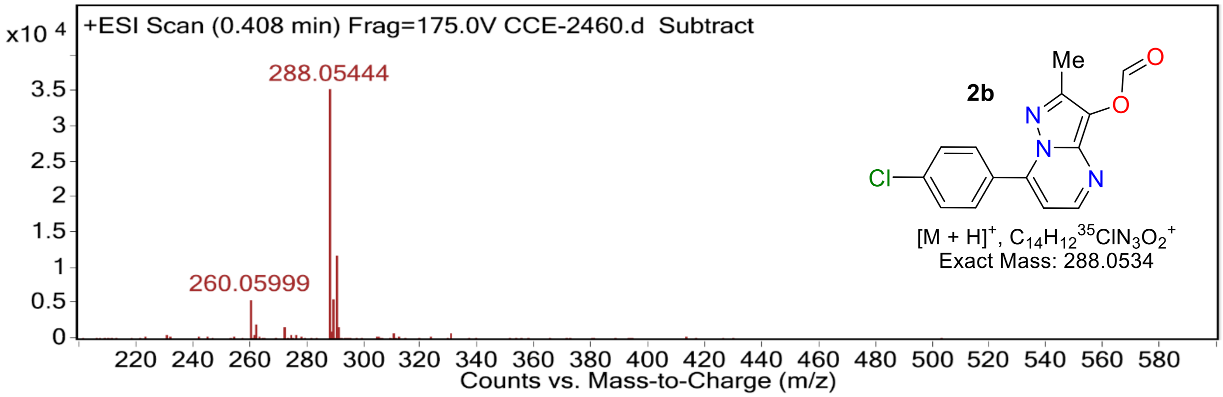

Peak List

m/z	z	Abund	Abund %
81.94189		2133.7	6.04
260.05999		5574.3	15.77
262.05436		2045.4	5.79
288.05444	1	35343.6	100
289.05633	1	5701.5	16.13
290.05222	1	11920.7	33.73

Fig. S4 HRMS analysis of 7-(4-chlorophenyl)-2-methylpyrazolo[1,5-*a*]pyrimidin-3-yl formate (**2b**).

User Spectra

Fragmentor Voltage	Collision Energy	Ionization Mode
175	0	ESI

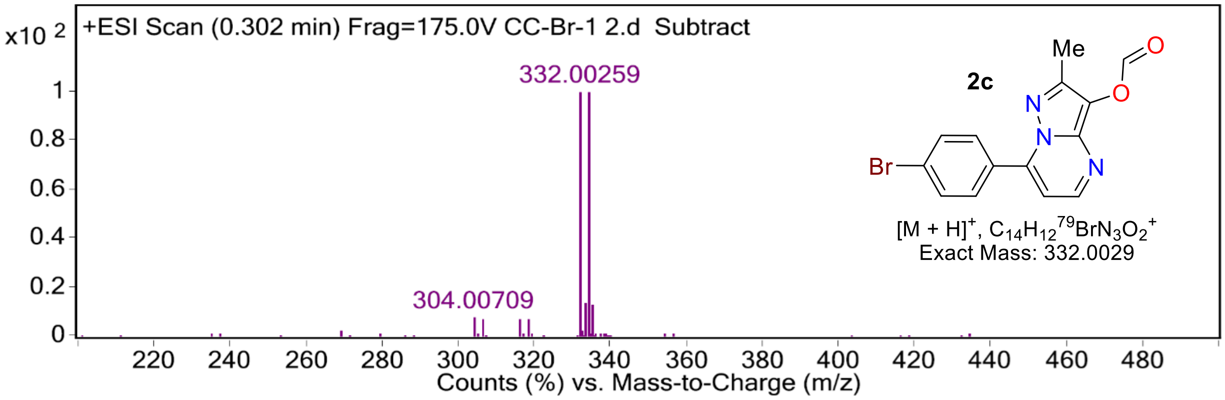

Peak List

m/z	z	Abund	Abund %
184.98358		13409.8	9.33
304.00709		11238.6	7.82
306.00579		10867.2	7.56
316.00735		10278.1	7.15
318.00579		10237.9	7.12
332.00259	1	143734.7	100
333.00484	1	19868.5	13.82
334.00039	1	143300	99.7

Fig. S5 HRMS analysis of 7-(4-bromophenyl)-2-methylpyrazolo[1,5-*a*]pyrimidin-3-yl formate (**2c**).

User Spectra

Fragmentor Voltage	Collision Energy	Ionization Mode
175	0	ESI

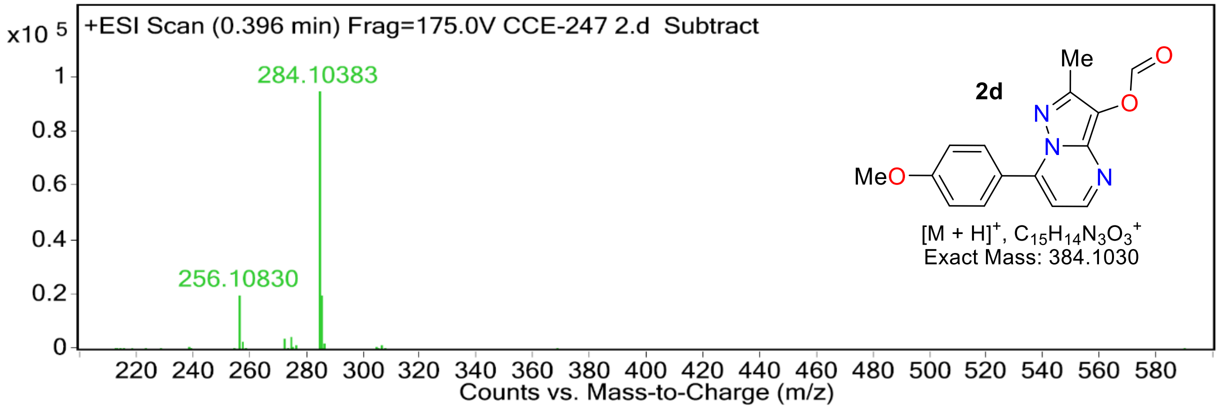

Peak List

m/z	z	Abund	Abund %
256.1083		19782.3	20.84
284.10383	1	94908.8	100
285.107	1	20045.3	21.12

Fig. S6 HRMS analysis of 7-(4-methoxyphenyl)-2-methylpyrazolo[1,5-*a*]pyrimidin-3-yl formate (**2d**).

User Spectra

Fragmentor Voltage	Collision Energy	Ionization Mode
175	0	ESI

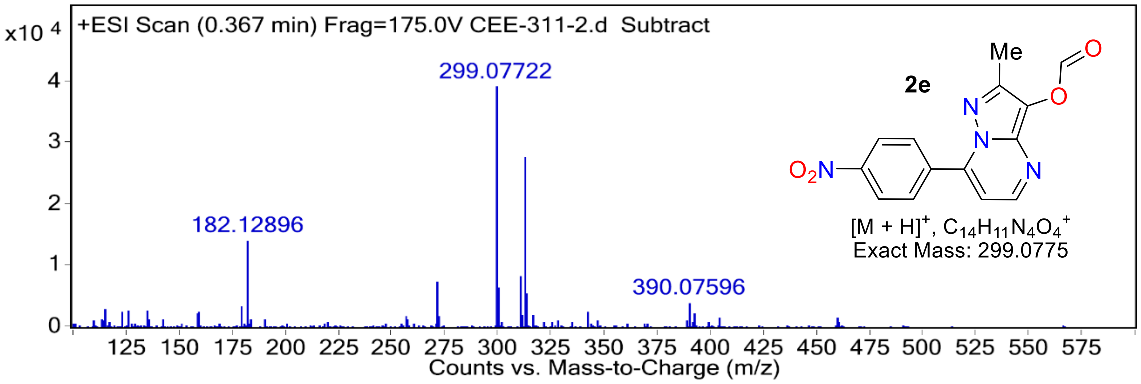

Peak List

m/z	z	Abund	Abund %
81.93831		4257.8	10.84
182.12896		14203.1	36.15
271.08223		7515.3	19.13
299.07722	1	39291.6	100
300.07911	1	6597	16.79
310.1559	1	8432.8	21.46
312.1704	1	27640	70.35
390.07596		4043.5	10.29

Fig. S7 HRMS analysis of 7-(4-nitrophenyl)-2-methylpyrazolo[1,5-*a*]pyrimidin-3-yl formate (**2e**).

User Spectra

Fragmentor Voltage	Collision Energy	Ionization Mode
175	0	ESI

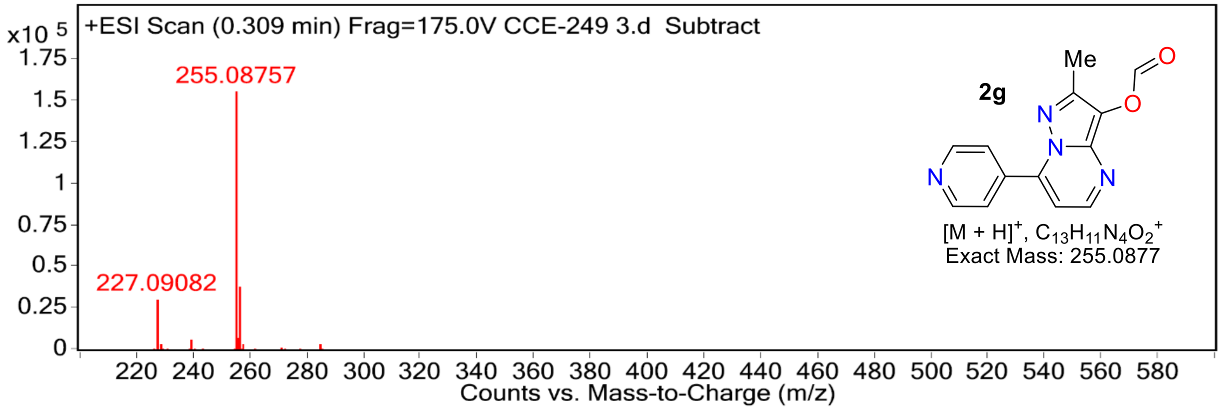

Peak List

m/z	z	Abund	Abund %
131.05827		8061.5	5.18
158.06958		23998.2	15.41
227.09082		30350.2	19.48
255.08757	1	155773.5	100
256.08929	1	37928.4	24.35

Fig. S8 HRMS analysis of 2-methyl-7-(4-pyridyl)pyrazolo[1,5-*a*]pyrimidin-3-yl formate (**2g**).

User Spectra

Fragmentor Voltage	Collision Energy	Ionization Mode
175	0	ESI

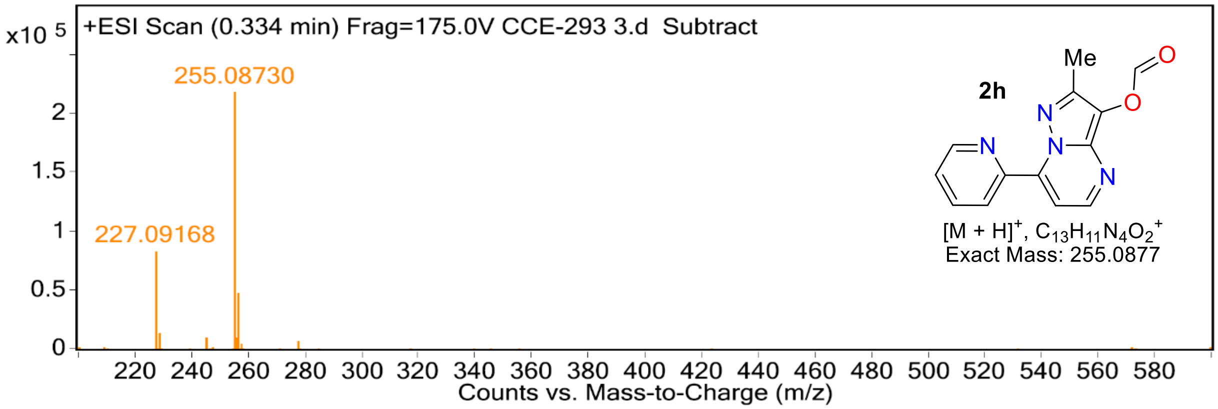

Peak List

m/z	z	Abund	Abund %
158.06931		28026	12.82
199.09608		10962.4	5.02
227.09168	1	83745.9	38.31
228.09338	1	13810.2	6.32
255.0873	1	218575.3	100
256.08922	1	48098.1	22.01

Fig. S9 HRMS analysis of 2-methyl-7-(2-pyridyl)pyrazolo[1,5-*a*]pyrimidin-3-yl formate (**2h**).

User Spectra

Fragmentor Voltage	Collision Energy	Ionization Mode
175	0	ESI

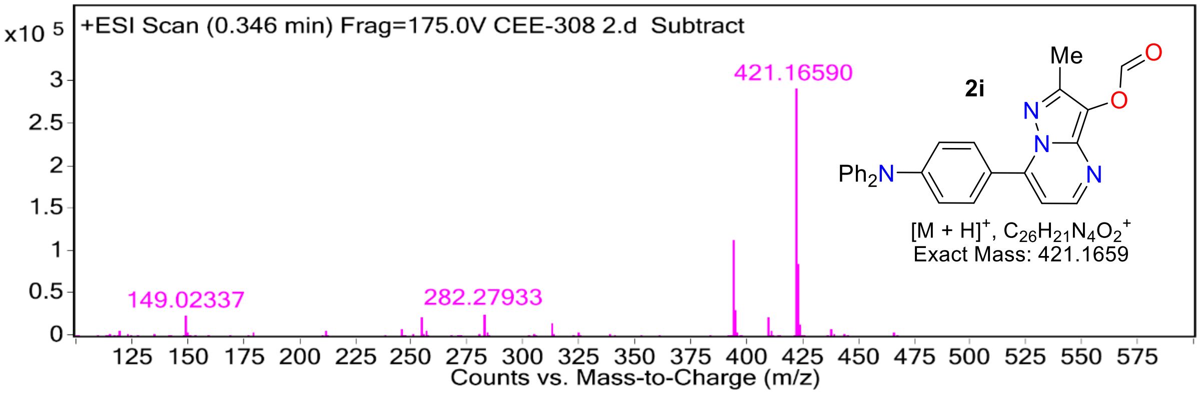

Peak List

m/z	z	Abund	Abund %
149.02337		23761	8.13
254.13087		23225.9	7.95
282.27933		26931.8	9.21
312.14923		16424.7	5.62
393.17063	1	114394.1	39.14
394.17344	1	31856.9	10.9
409.16562		22623.6	7.74
421.1659	1	292277.5	100

Fig. S10 HRMS analysis of 7-(4-Ph_2Nphenyl)-2-methylpyrazolo[1,5-*a*]pyrimidin-3-yl formate (**2i**).

User Spectra

Fragmentor Voltage	Collision Energy	Ionization Mode
175	0	ESI

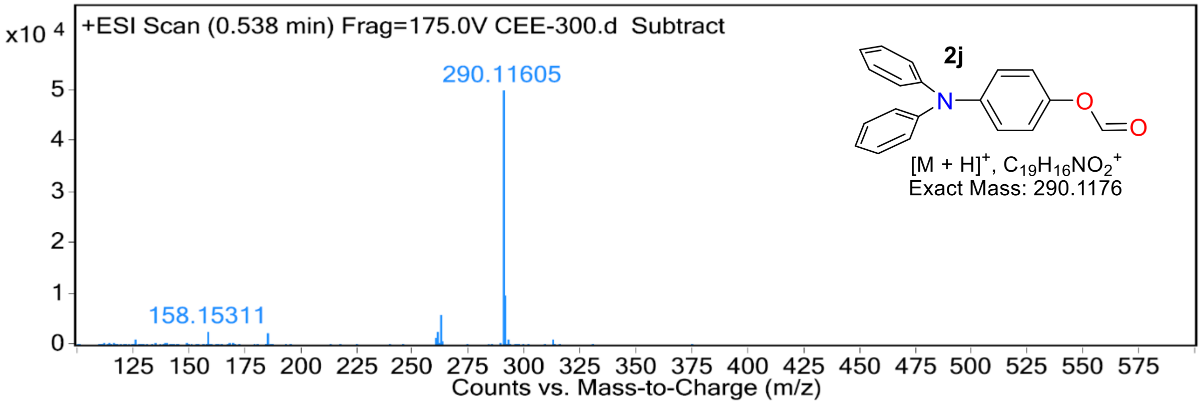

Peak List

m/z	z	Abund	Abund %
158.15311		2574.9	5.16
261.11485		2542.9	5.09
262.12013		6080.6	12.18
290.11605	1	49924.8	100
290.18275		4665.4	9.34
291.11861	1	9783.4	19.6

Fig. S11 HRMS analysis of 4-(diphenylamino)phenyl formate (**2j**).

User Spectra

Fragmentor Voltage	Collision Energy	Ionization Mode
80	0	ESI

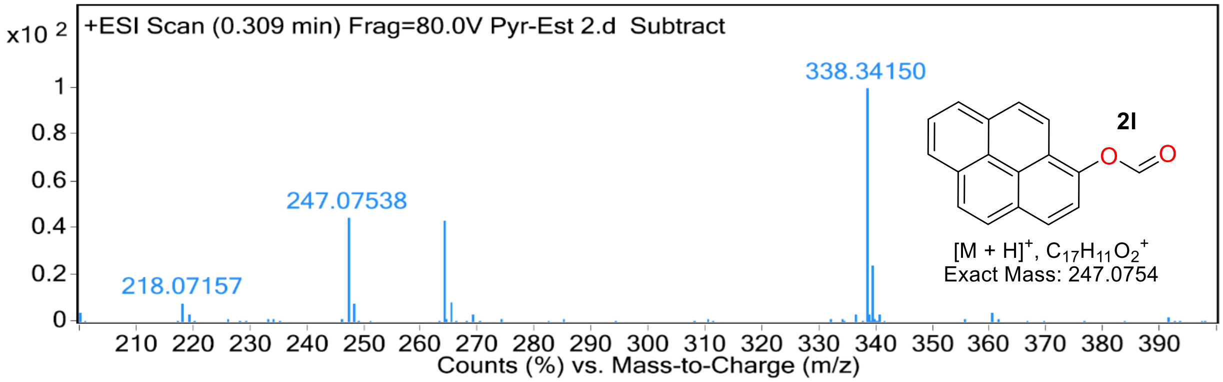

Peak List

m/z	z	Abund	Abund %
85.05914		42523.1	33.94
89.50712		18918.5	15.1
218.07157		10407.7	8.31
247.07538	1	55987.2	44.68
264.10198	1	54201.1	43.26
265.10487	1	10592.1	8.45
338.3415	1	125296.5	100
339.34413	1	30313.3	24.19

Fig. S12 HRMS analysis of pyren-1-yl formate (**2l**).

User Spectra

Fragmentor Voltage	Collision Energy	Ionization Mode
175	0	ESI

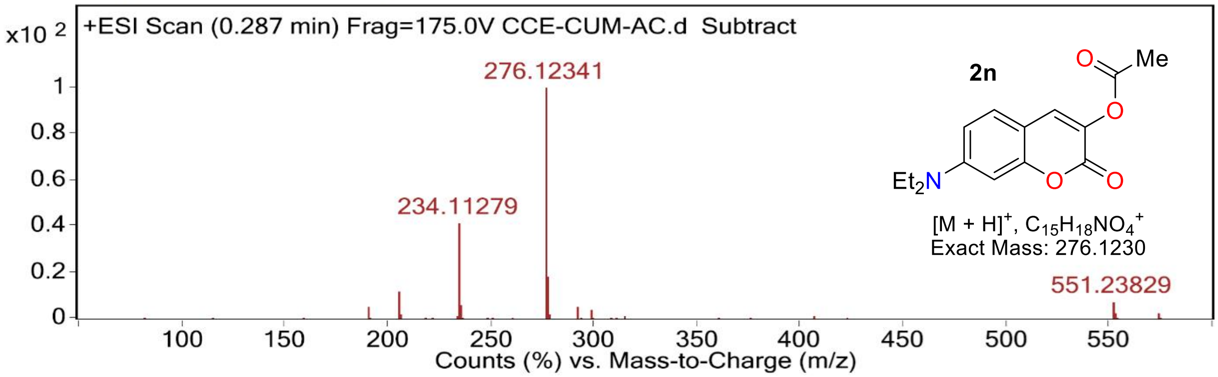

Peak List

m/z	z	Abund	Abund %
190.0498		22294.8	5.49
205.07321		47854.4	11.78
234.11279	1	168638.6	41.52
235.11544	1	23533	5.79
276.12341	1	406160.7	100
277.12663	1	74135.1	18.25
292.11794		22124.7	5.45
551.23829		30752.9	7.57

Fig. S13 HRMS analysis of 7-(diethylamino)coumarin-3-yl acetate (**2n**).

User Spectra

Fragmentor Voltage	Collision Energy	Ionization Mode
175	0	ESI

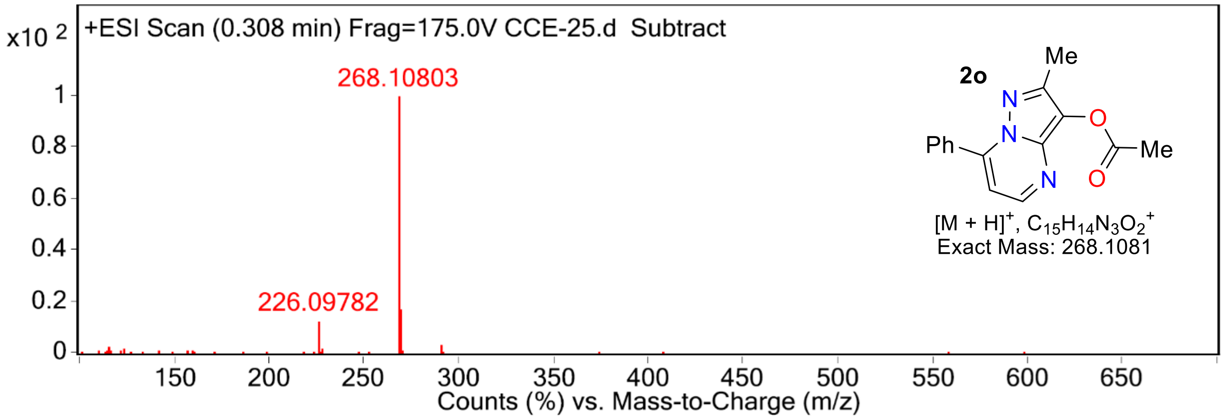

Peak List

m/z	z	Abund	Abund %
226.09782		18735.1	12.88
268.10803	1	145426.3	100
269.1106	1	24653.6	16.95

Fig. S14 HRMS analysis of 2-methyl-7-phenylpyrazolo[1,5-*a*]pyrimidin-3-yl acetate (**2o**).

User Spectra

Fragmentor Voltage	Collision Energy	Ionization Mode
175	0	ESI

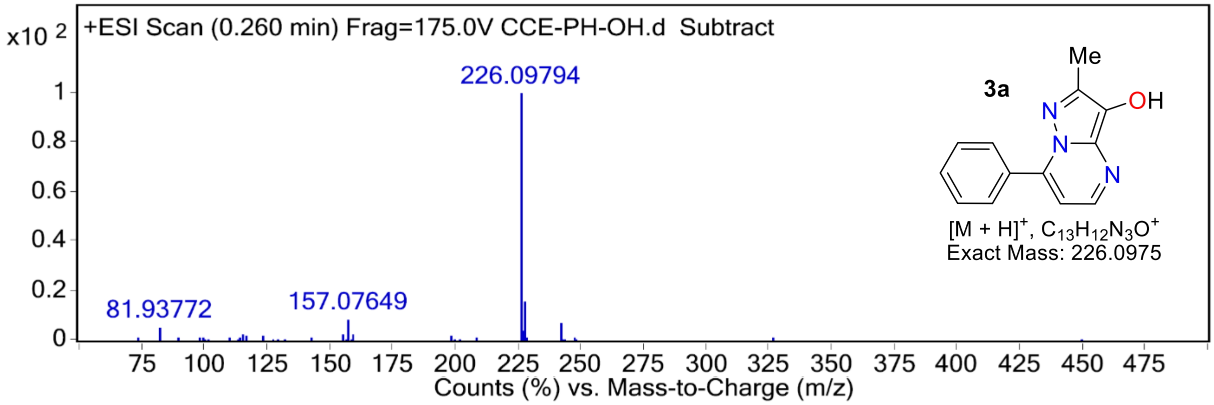

Peak List

m/z	z	Abund	Abund %
81.93772		13835.7	5.59
157.07649		21894.2	8.85
226.09794	1	247358.7	100
226.17182		13165.8	5.32
227.10061	1	39091.3	15.8
242.093		18505.1	7.48

Fig. S15 HRMS analysis of 2-methyl-7-phenylpyrazolo[1,5-*a*]pyrimidin-3-ol (**3a**)

User Spectra

Fragmentor Voltage	Collision Energy	Ionization Mode
175	0	ESI

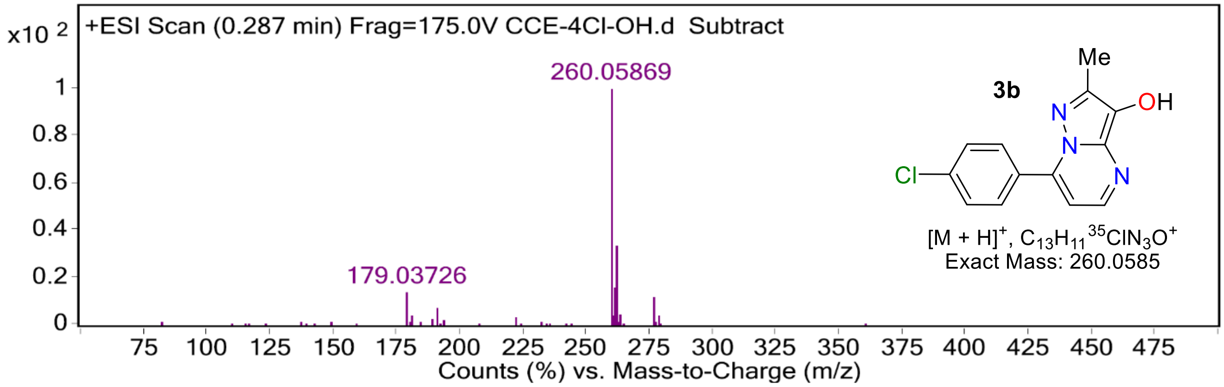

Peak List

m/z	z	Abund	Abund %
179.03726		67080.4	13.82
191.03747		36514.4	7.52
260.05869	1	485400.4	100
261.06138	1	78329.4	16.14
262.05599	1	162053.4	33.39
276.05386		58774.5	12.11

Fig. S16 HRMS analysis of 7-(4-chlorophenyl)-2-methylpyrazolo[1,5-*a*]pyrimidin-3-ol (**3b**).

User Spectra

Fragmentor Voltage	Collision Energy	Ionization Mode
175	0	ESI

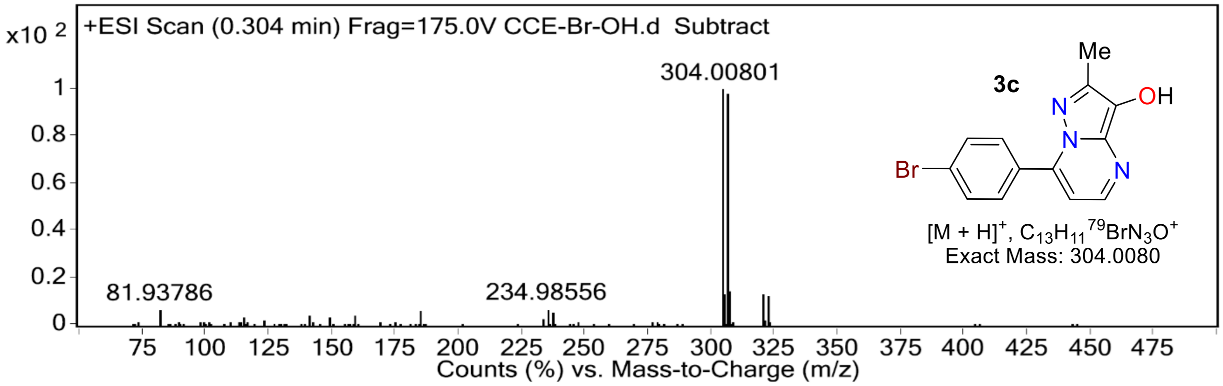

Peak List

m/z	z	Abund	Abund %
81.93786		4763.8	6.68
184.98291		4328	6.07
234.98556		4990	7
236.98368		3903	5.47
304.00801	1	71312.5	100
305.01082	1	9594.5	13.45
306.00622	1	69870.9	97.98
307.00961	1	10380.1	14.56

Fig. S17 HRMS analysis of 7-(4-bromophenyl)-2-methylpyrazolo[1,5-*a*]pyrimidin-3-ol (**3c**).

User Spectra

Fragmentor Voltage	Collision Energy	Ionization Mode
175	0	ESI

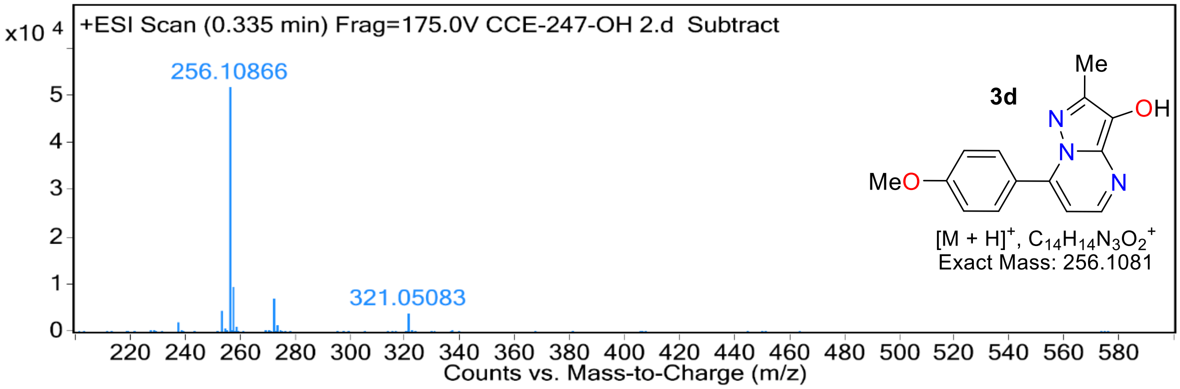

Peak List

m/z	z	Abund	Abund %
169.01132		11532	22.2
187.08446		4039.8	7.78
253.06317		4598.2	8.85
256.10866	1	51936.4	100
257.11075	1	9699.6	18.68
272.1032		7261.7	13.98
321.05083		3927.8	7.56

Fig. S18 HRMS analysis of 7-(4-methoxyphenyl)-2-methylpyrazolo[1,5-*a*]pyrimidin-3-ol (**3d**).

User Spectra

Fragmentor Voltage	Collision Energy	Ionization Mode
175	0	ESI

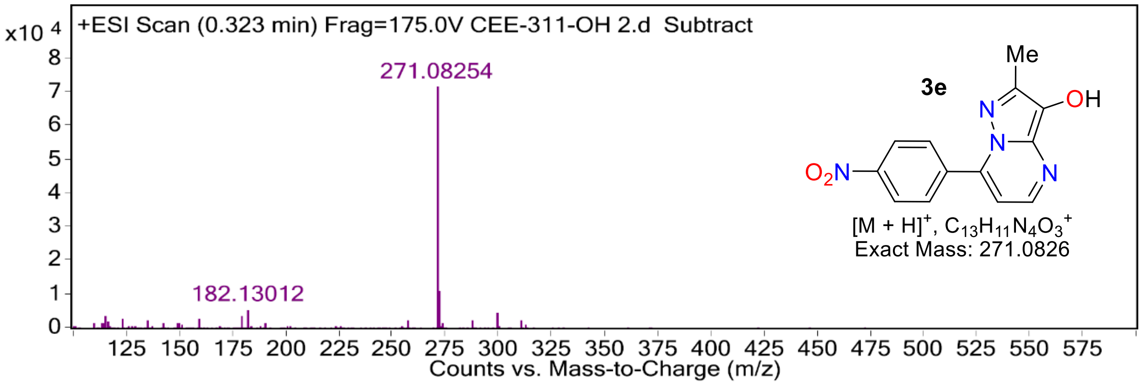

Peak List

m/z	z	Abund	Abund %
81.93823		6546.7	9.12
114.9845		3983.6	5.55
178.94486		4021.5	5.6
182.13012		5634.3	7.85
271.08254	1	71813.6	100
271.16675		4931.8	6.87
272.08444	1	11338.1	15.79
299.07869		4530.8	6.31

Fig. S19 HRMS analysis of 7-(4-nitrophenyl)-2-methylpyrazolo[1,5-*a*]pyrimidin-3-ol (**3e**).

User Spectra

Fragmentor Voltage	Collision Energy	Ionization Mode
175	0	ESI

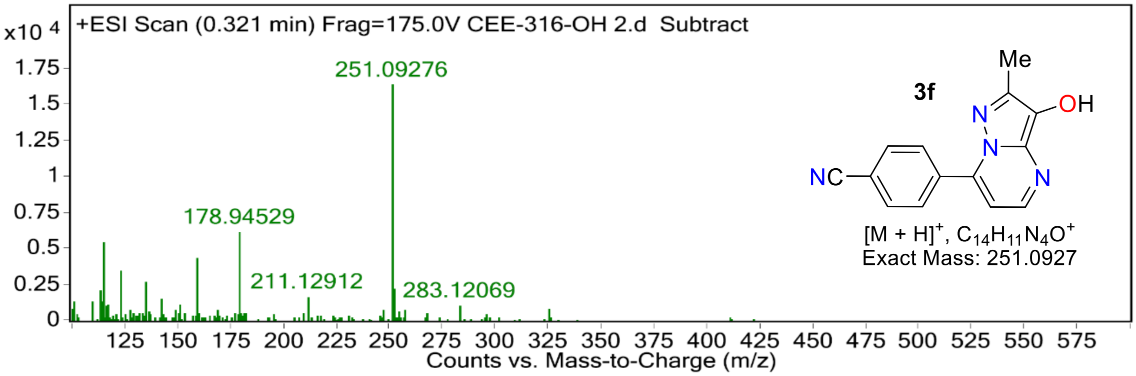

Peak List

m/z	z	Abund	Abund %
114.98406		5480	33.38
122.96458		3583.3	21.82
134.9551		2759	16.8
141.95856		1555.4	9.47
158.97242		4407.4	26.84
178.94529		6197.7	37.75
211.12912		1702.9	10.37
251.09276	1	16418.9	100
251.13339		2794.8	17.02
252.09282	1	2304.9	14.04

Fig. S20 HRMS analysis of 7-(4-cyanophenyl)-2-methylpyrazolo[1,5-*a*]pyrimidin-3-ol (**3f**).

User Spectra

Fragmentor Voltage	Collision Energy	Ionization Mode
175	0	ESI

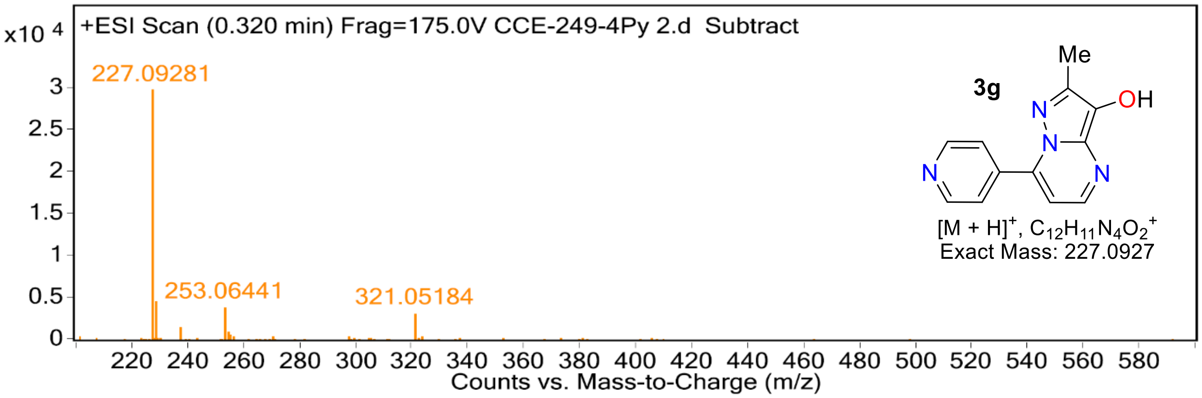

Peak List

m/z	z	Abund	Abund %
158.07192		2783.4	9.33
169.01176		10403.7	34.86
227.09281	1	29841.6	100
228.09398	1	4675.7	15.67
236.99801		1642.9	5.51
253.06441		3940.8	13.21
321.05184		3244.2	10.87

Fig. S21 HRMS analysis of 2-methyl-7-(4-pyridyl)pyrazolo[1,5-*a*]pyrimidin-3-ol (**3g**).

User Spectra

Fragmentor Voltage	Collision Energy	Ionization Mode
175	0	ESI

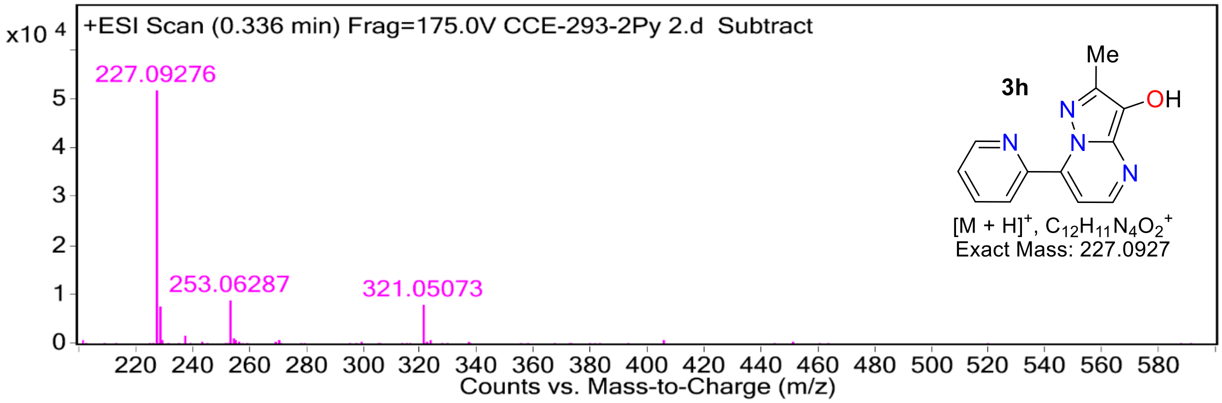

Peak List

m/z	z	Abund	Abund %
158.07004		5317.7	10.25
169.01138		14248.6	27.47
227.09276	1	51870.6	100
228.09608	1	7774	14.99
253.06287		8890.9	17.14
321.05073		7965	15.36

Fig. S22 HRMS analysis of 2-methyl-7-(2-pyridyl)pyrazolo[1,5-*a*]pyrimidin-3-ol (**3h**).

User Spectra

Fragmentor Voltage	Collision Energy	Ionization Mode
175	0	ESI

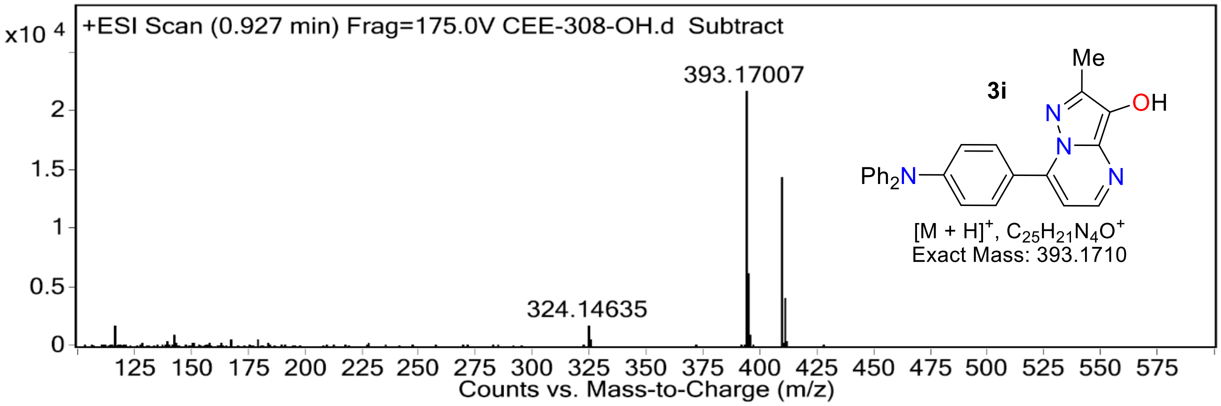

Peak List

m/z	z	Abund	Abund %
116.01598		1824.2	8.38
324.14635		1772.2	8.14
393.17007	1	21763.2	100
394.17263	1	6227.6	28.62
409.16643	1	14476.5	66.52
409.25624		1553.2	7.14
410.16484	1	4186.9	19.24

Fig. S23 HRMS analysis of 7-(4-Ph_2Nphenyl)-2-methylpyrazolo[1,5-*a*]pyrimidin-3-ol (**3i**).

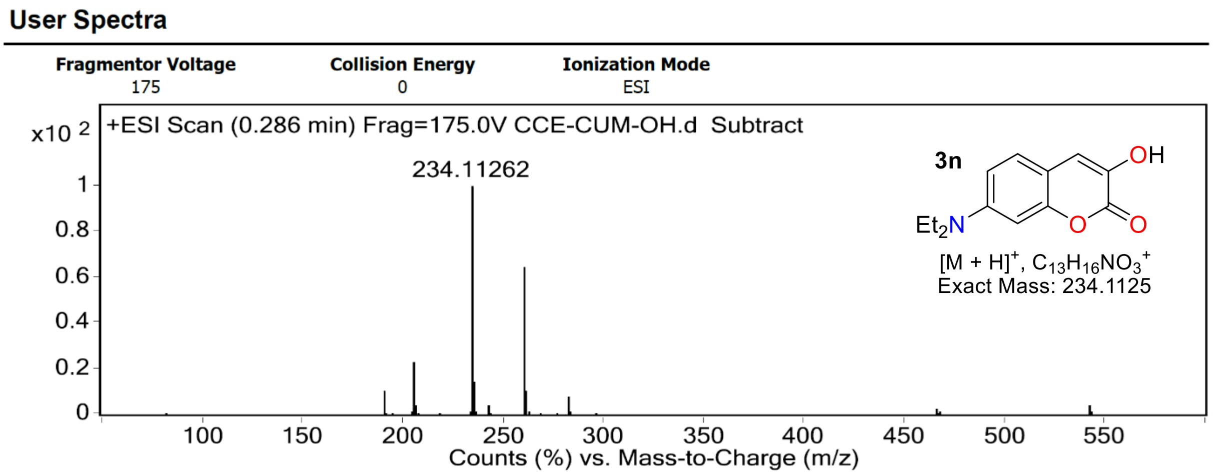

Peak List

m/z	z	Abund	Abund %
190.05037		95930.2	10.59
205.07392		207076.3	22.85
234.11262	1	906238.8	100
234.14778		135758.3	14.98
235.11614	1	134683.7	14.86
260.12843	1	580422.8	64.05
261.13155	1	94171.2	10.39
282.11025		72993	8.05

Fig. S24 HRMS analysis of 7-(diethylamino)coumarin-3-ol (**3n**).

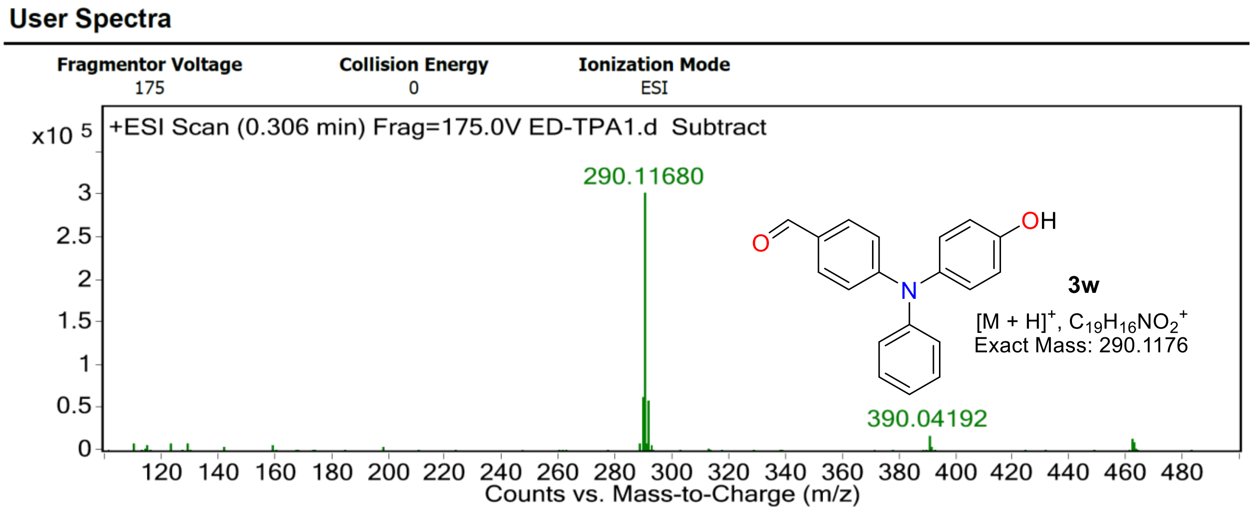

Peak List

m/z	z	Abund	Abund %
81.93792	1	22868.2	7.55
289.10903	1	62738.3	20.72
290.1168	1	302824.7	100
291.11981	1	59878.9	19.77
390.04192	1	18080.5	5.97

Fig. S25 HRMS analysis of 4-((4-hydroxyphenyl)(phenyl)amino)benzaldehyde (**3w**).

User Spectra

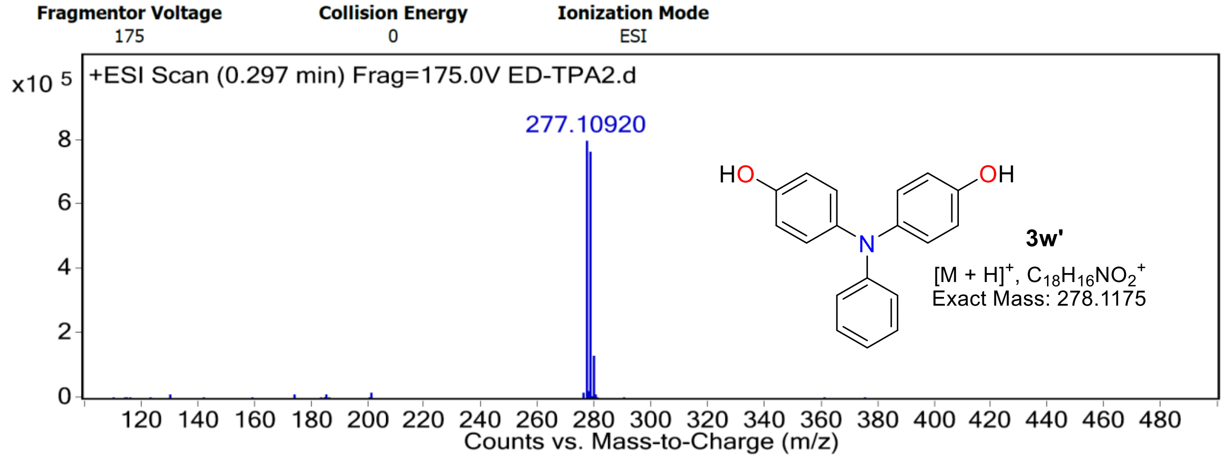

Peak List

m/z	z	Abund	Abund %
277.1092	1	799051.3	100
277.14693	1	95780.1	11.99
277.18904	1	47716.1	5.97
278.11593	1	766871.1	95.97
278.15548	1	98192.8	12.29
279.11967	1	132648.4	16.6

Fig. S26 HRMS analysis of 4,4'-(phenylazanediyl)diphenol (**3w'**).

User Spectra

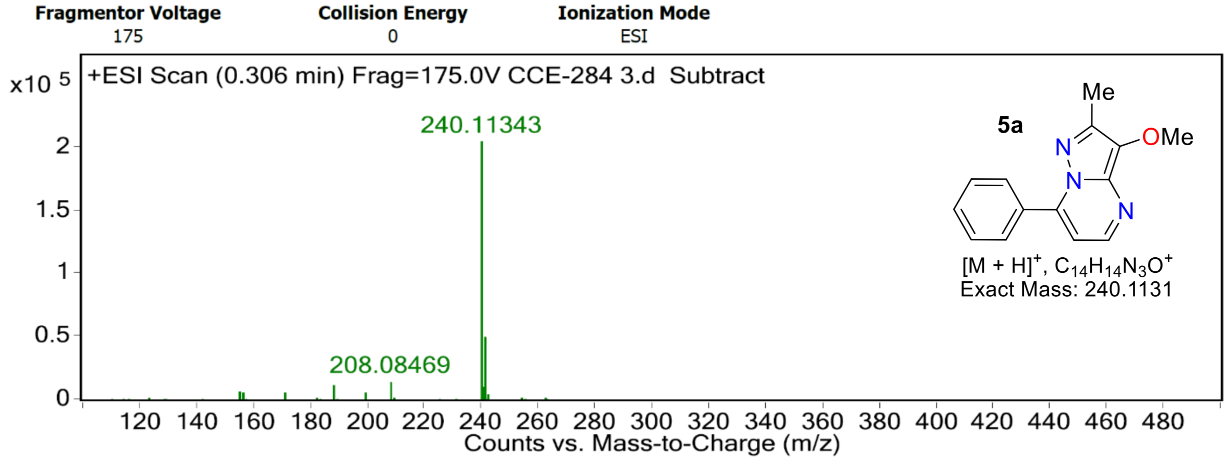

Peak List

m/z	z	Abund	Abund %
188.05648		12268.7	5.99
208.08469		14527.6	7.09
240.11343	1	204983	100
240.29178		10660.8	5.2
241.1145	1	50562.7	24.67

Fig. S27 HRMS analysis of 3-methoxy- 2-methyl-7-phenylpyrazolo[1,5-*a*]pyrimidine (**5a**)

User Spectra

Fragmentor Voltage	Collision Energy	Ionization Mode
175	0	ESI

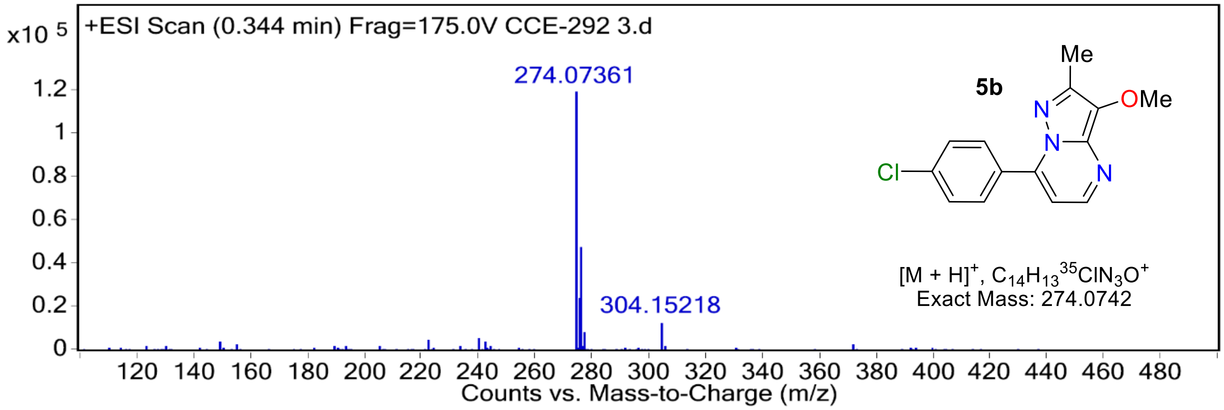

Peak List

m/z	z	Abund	Abund %
274.07361	1	119614.1	100
275.07505	1	24390.4	20.39
276.06996	1	48074.8	40.19
277.07206	1	8280.1	6.92
304.15218		13134.1	10.98

Fig. S28 HRMS analysis of 7-(4-chlorophenyl)-3-methoxy-2-methylpyrazolo[1,5-*a*]pyrimidine (**5b**).

User Spectra

Fragmentor Voltage	Collision Energy	Ionization Mode
175	0	ESI

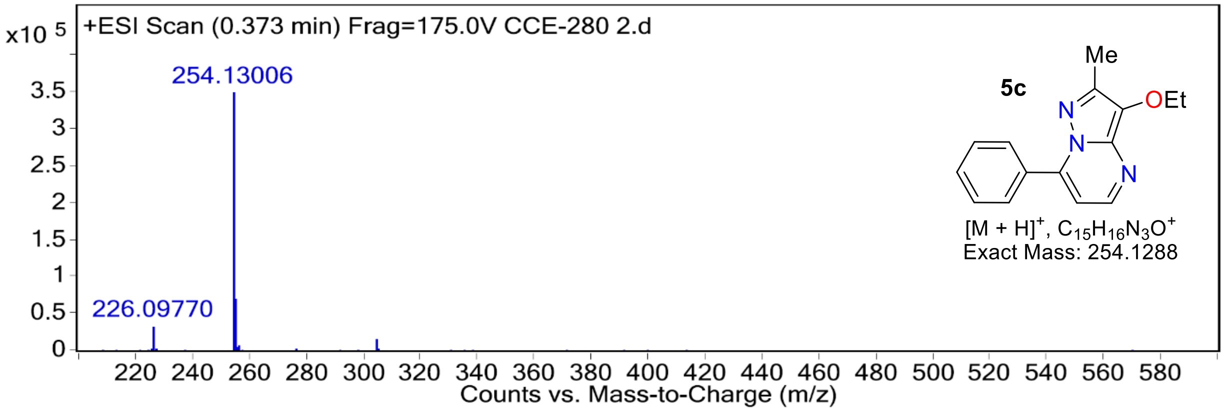

Peak List

m/z	z	Abund	Abund %
226.0977		34366.8	9.8
254.13006	1	350815.3	100
255.133	1	71095.6	20.27

Fig. S29 HRMS analysis of 3-ethoxy- 2-methyl-7-phenylpyrazolo[1,5-*a*]pyrimidine (**5c**)

User Spectra

Fragmentor Voltage	Collision Energy	Ionization Mode
175	0	ESI

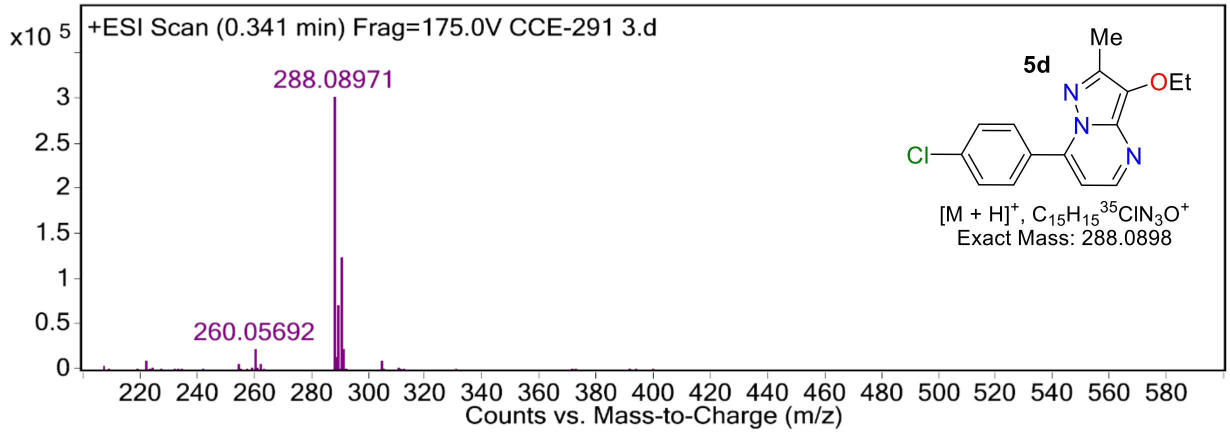

Peak List

m/z	z	Abund	Abund %
260.05692		22586.8	7.48
288.08971	1	301988.3	100
289.09138	1	71494.1	23.67
290.08626	1	123744.6	40.98
291.08802	1	23947.8	7.93

Fig. S30 HRMS analysis of 7-(4-chlorophenyl)-3-ethoxy-2-methylpyrazolo[1,5-*a*]pyrimidine (**5d**).

User Spectra

Fragmentor Voltage	Collision Energy	Ionization Mode
175	0	ESI

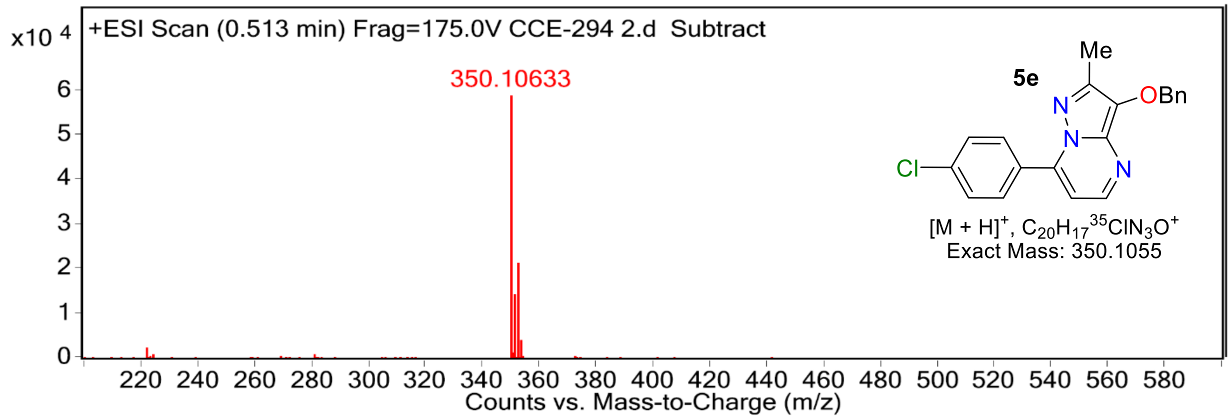

Peak List

m/z	z	Abund	Abund %
350.10633	1	58884.1	100
351.10883	1	14421.4	24.49
352.10345	1	21463.3	36.45
353.10515	1	4195.1	7.12

Fig. S31 HRMS analysis of 3-benzyloxy-7-(4-chlorophenyl)-2-methylpyrazolo[1,5-*a*]pyrimidine (**5e**).

User Spectra

Fragmentor Voltage	Collision Energy	Ionization Mode
175	0	ESI

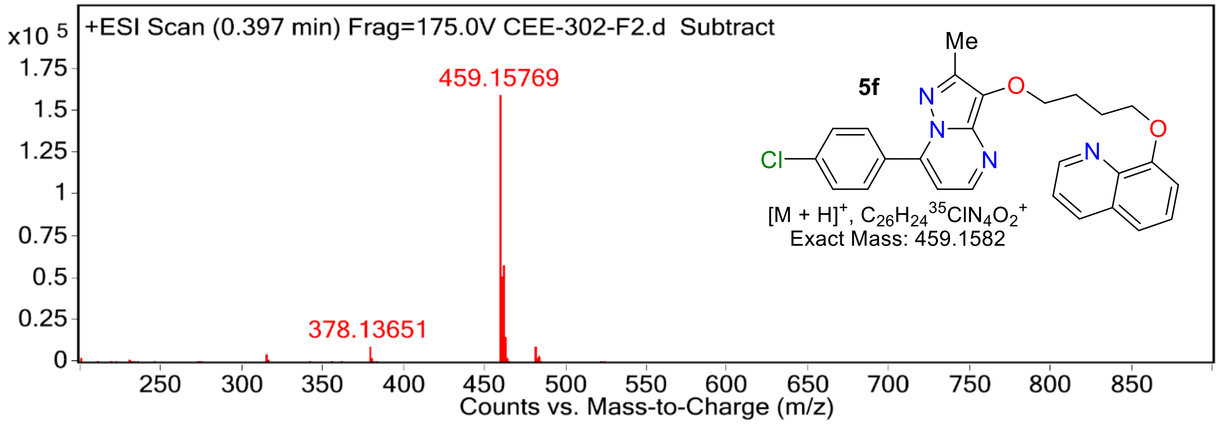

Peak List

m/z	z	Abund	Abund %
378.13651		9145.5	5.72
459.15769	1	159807.9	100
459.33397		8338.1	5.22
460.16022	1	51212.1	32.05
461.15542	1	57989.7	36.29
462.15868	1	15516	9.71
481.13893		9394.1	5.88

Fig. S32 HRMS analysis of hybrid compound **5f**.

User Spectra

Fragmentor Voltage	Collision Energy	Ionization Mode
175	0	ESI

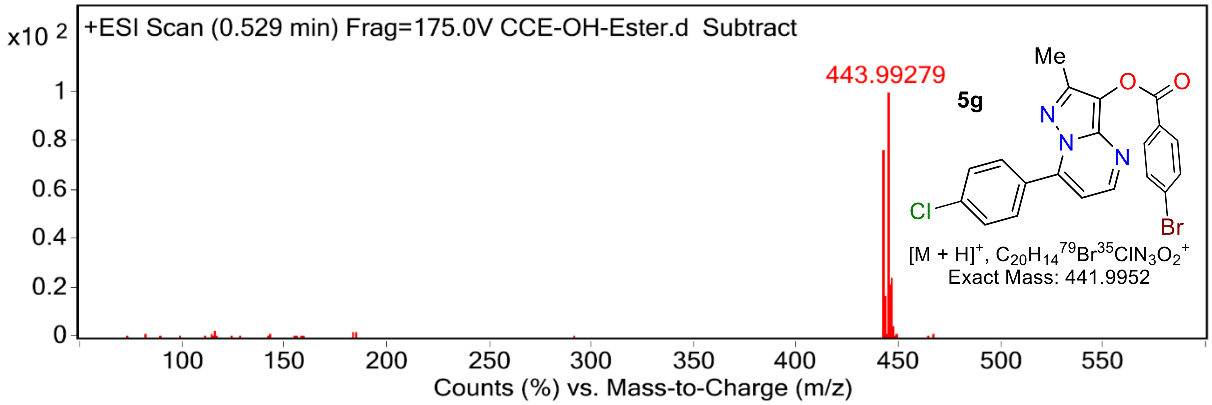

Peak List

m/z	z	Abund	Abund %
441.99533	1	44680.2	76.19
442.99715	1	9914.7	16.91
443.99279	1	58641.1	100
444.12527		2939.2	5.01
444.9956	1	12734.9	21.72
445.99241	1	14265.1	24.33

Fig. S33 HRMS of 7-(4-chlorophenyl)-2-methylpyrazolo[1,5-*a*]pyrimidin-3-yl 4-bromobenzoate (**5g**).

User Spectra

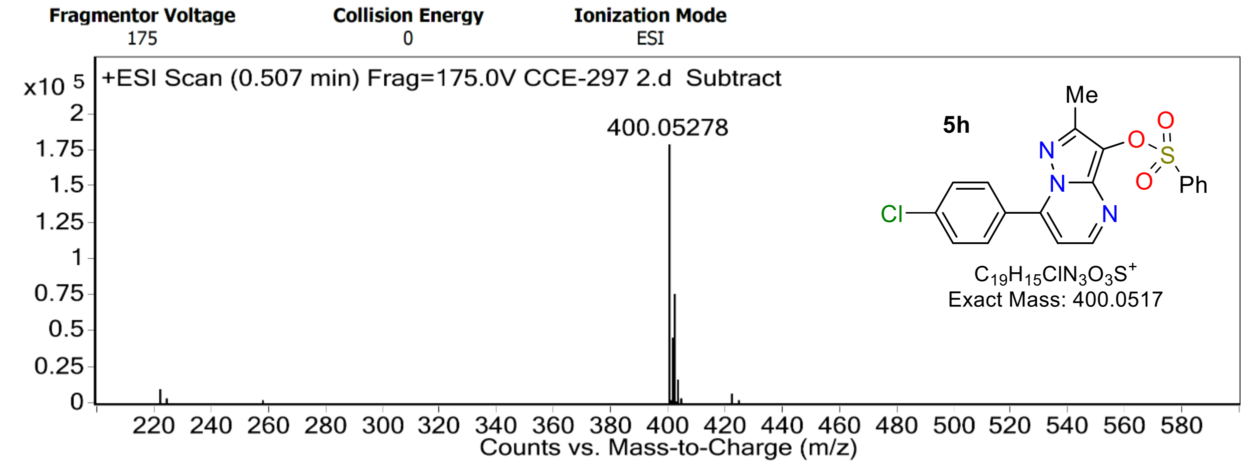

Peak List

m/z	z	Abund	Abund %
189.02184		12245.9	6.82
222.01889		11112.7	6.19
400.05278	1	179524.7	100
401.05462	1	45950.8	25.6
402.05001	1	76138.5	42.41
403.05186	1	16993.5	9.47

Fig. S34 HRMS of 7-(4-chlorophenyl)-2-methylpyrazolo[1,5-*a*]pyrimidin-3-yl benzenesulfonate (**5h**).

5. Copies of NMR spectra

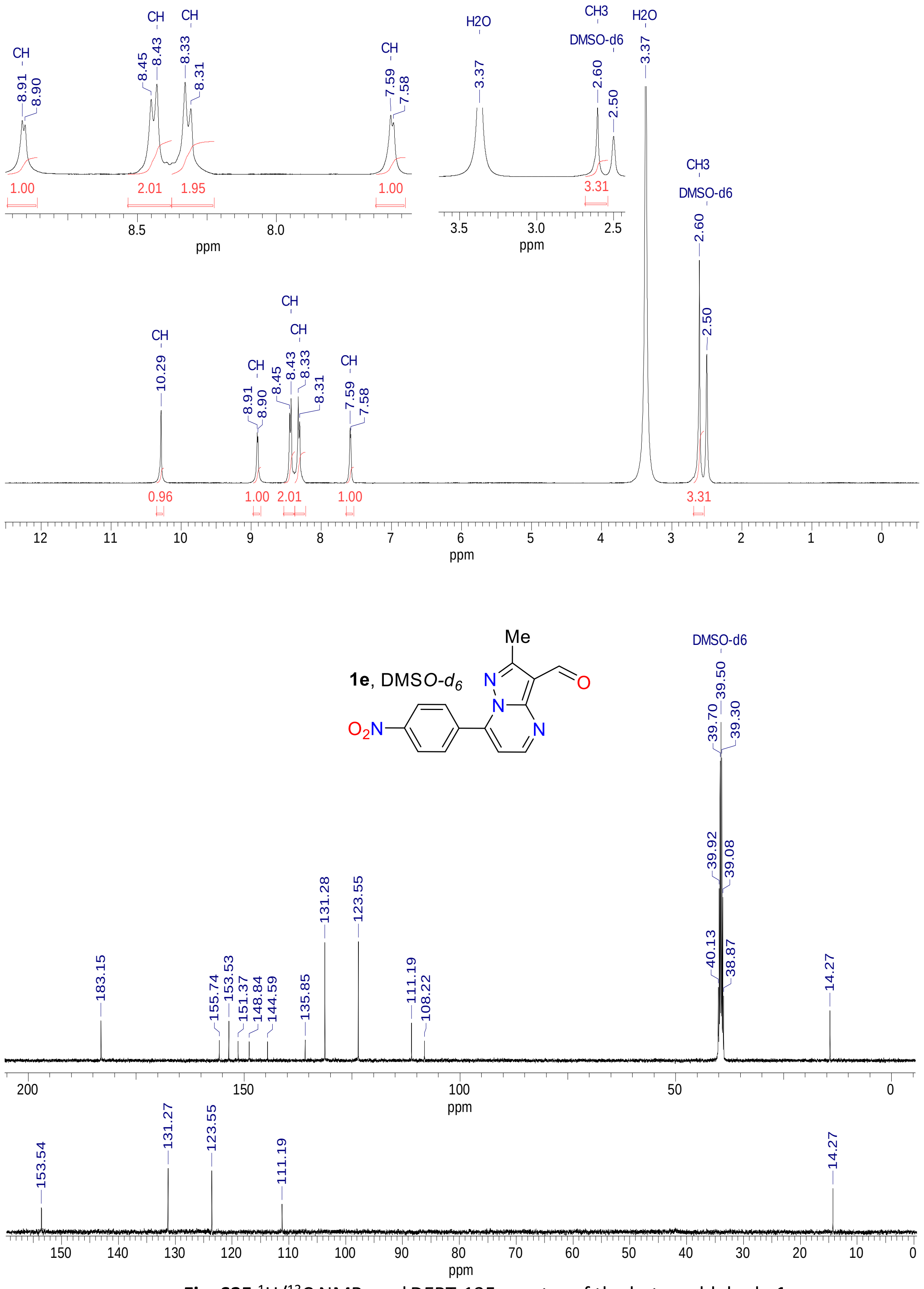

Fig. S35 $^{1}H/^{13}C$ NMR, and DEPT-135 spectra of the heteroaldehyde **1e**

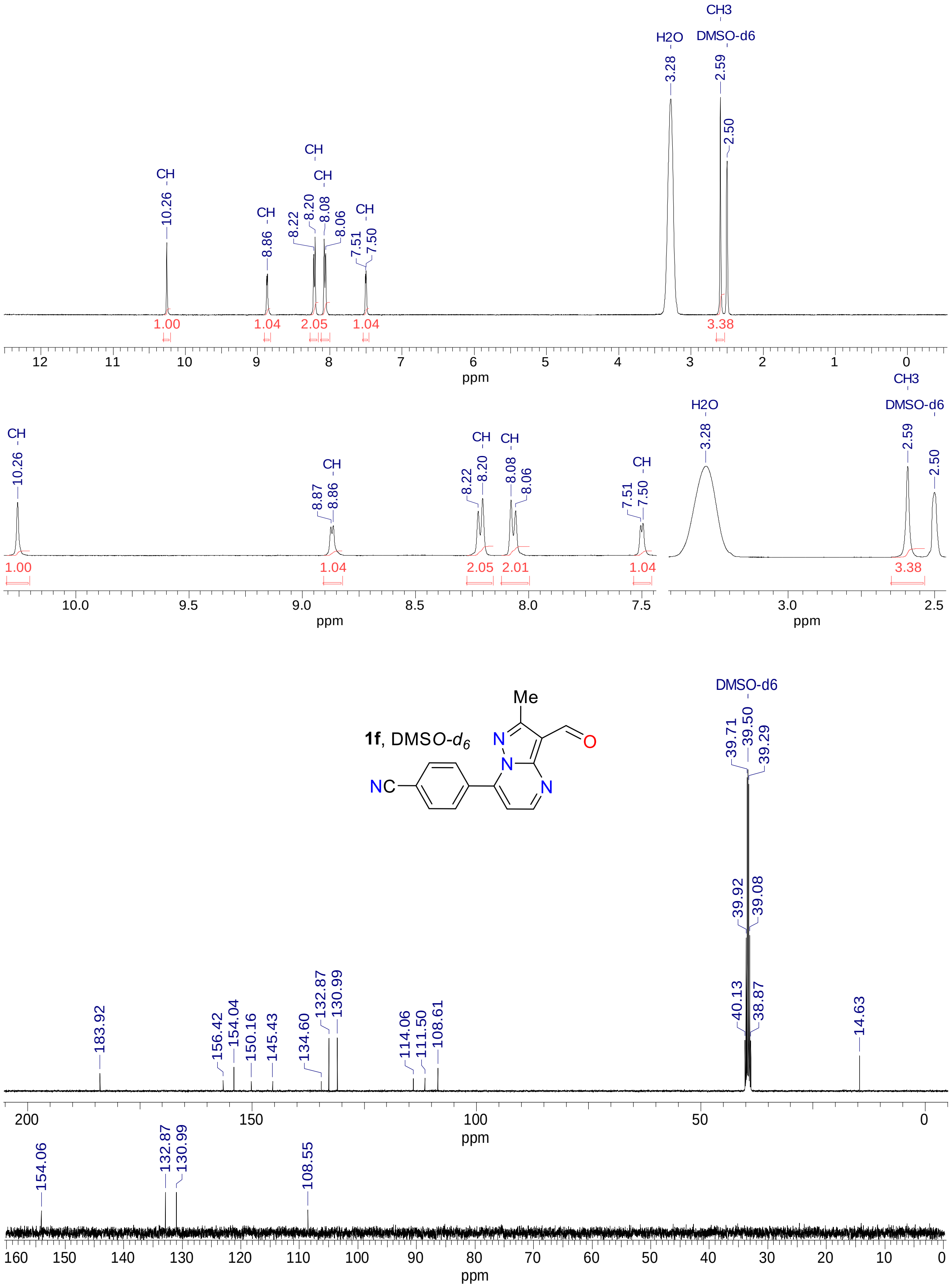

Fig. S36 ^{1}H/^{13}C NMR, and DEPT-135 spectra of the heteroaldehyde **1f**.

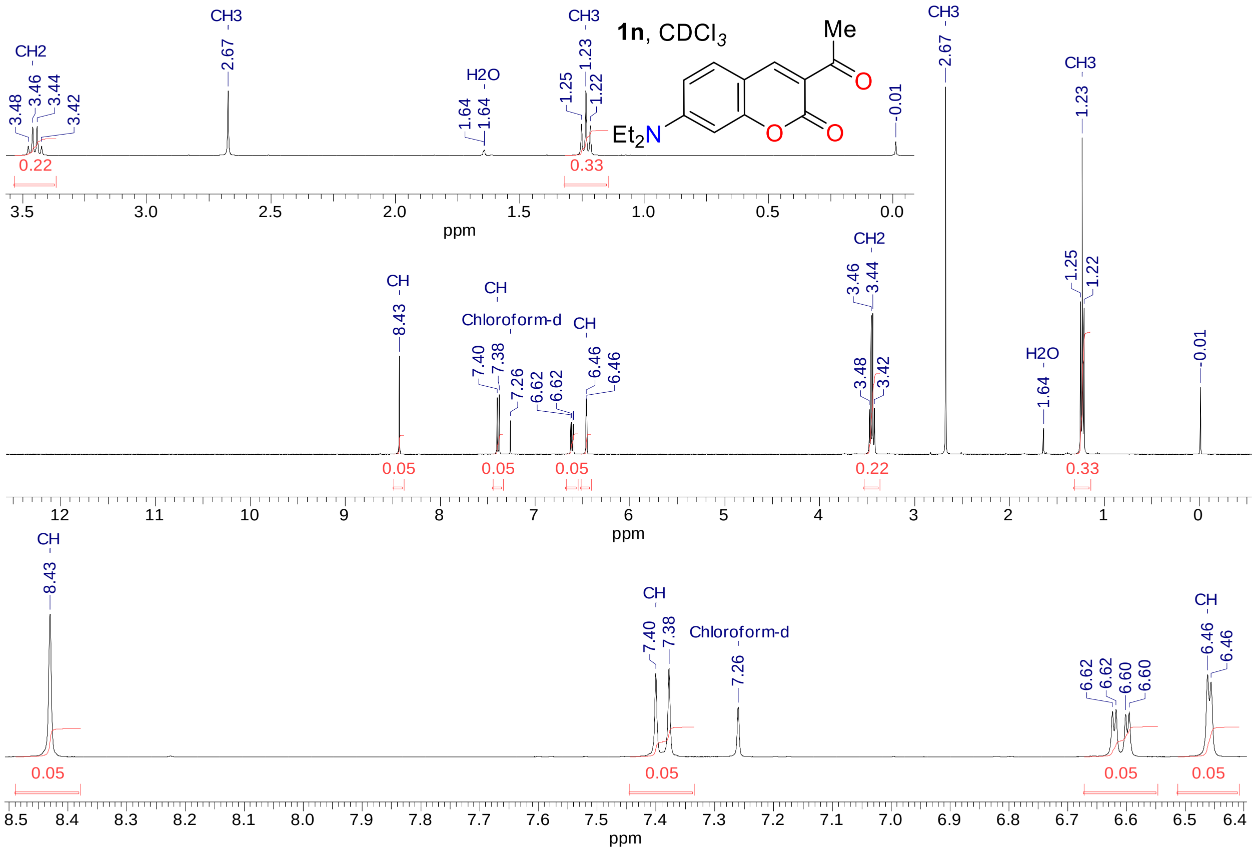

Fig. S37 ^{1}H NMR spectrum of 3-acetyl-7-diethylaminocoumarin **1n**.

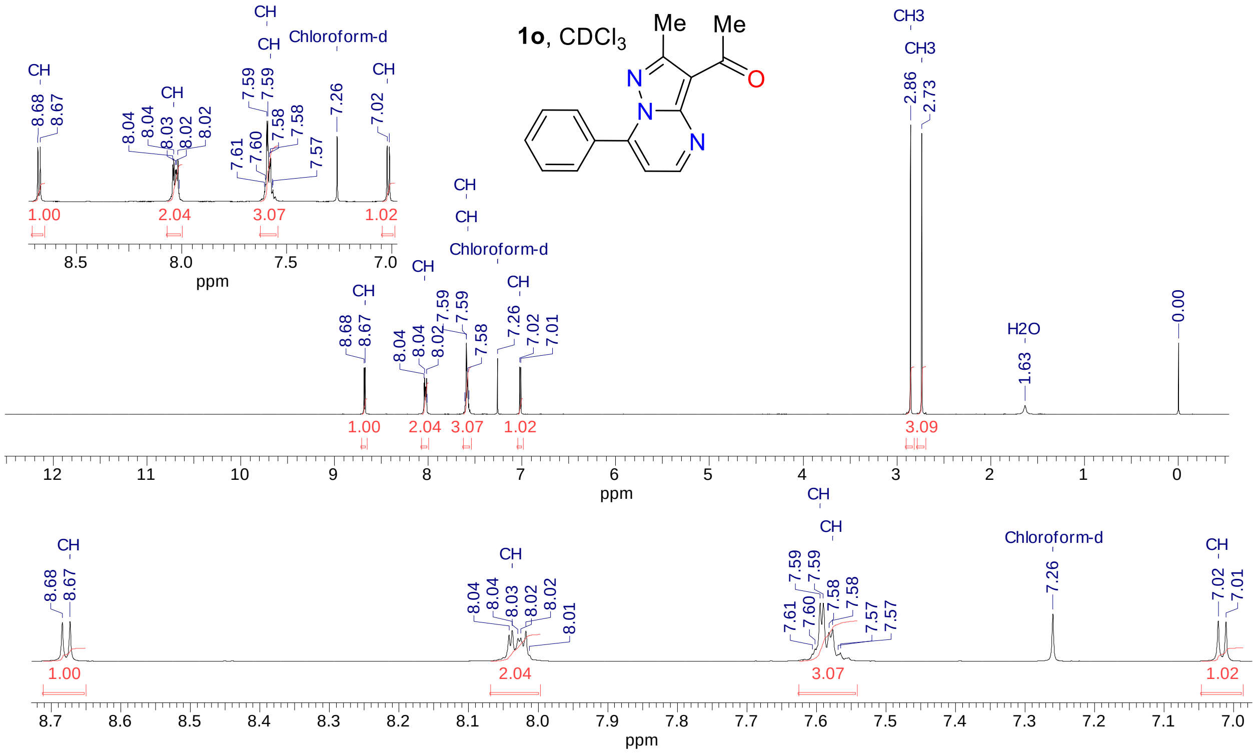

Fig. S38 ^{1}H NMR spectrum of the heteroaryl methyl ketone **1o**.

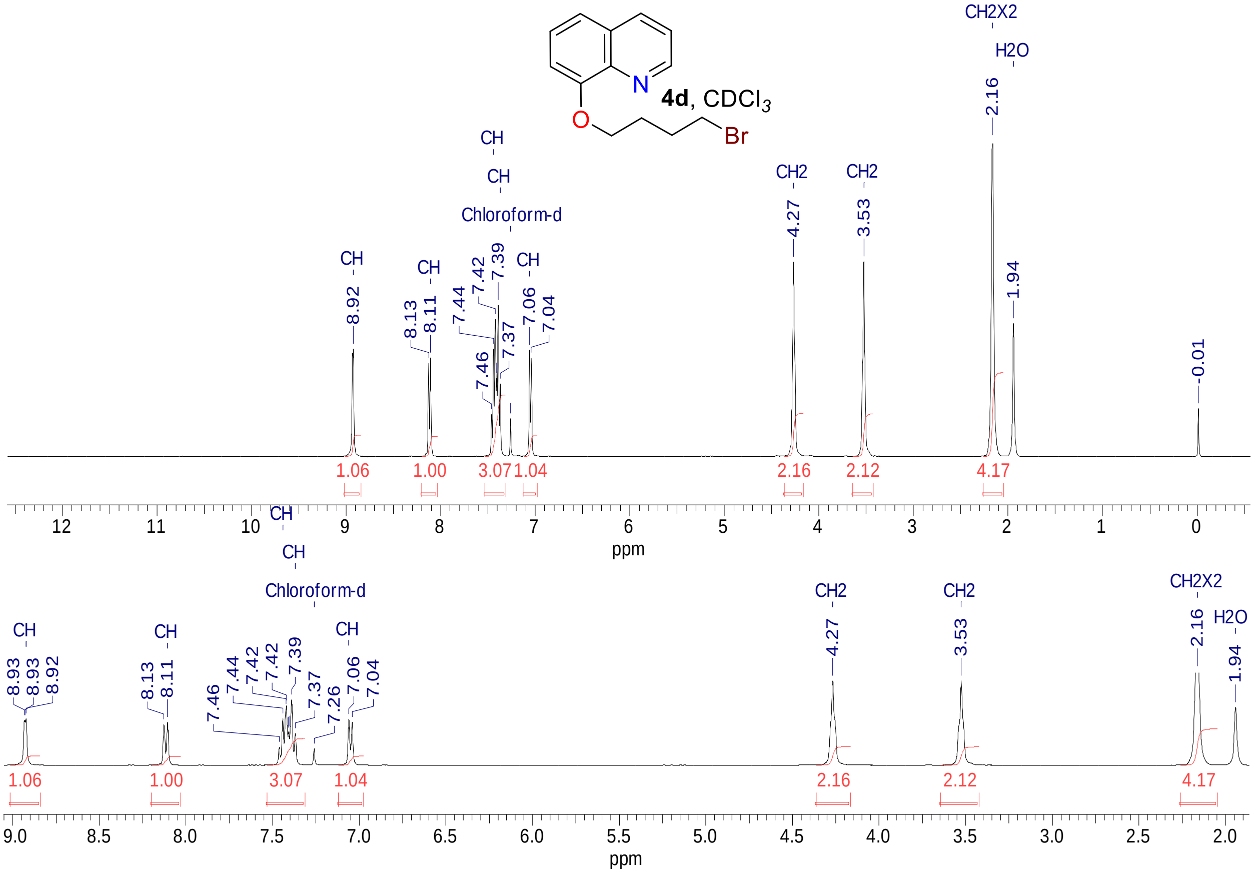

Fig. S39 ^{1}H NMR spectrum of 8-(4-bromobutoxy)quinoline **4d**.

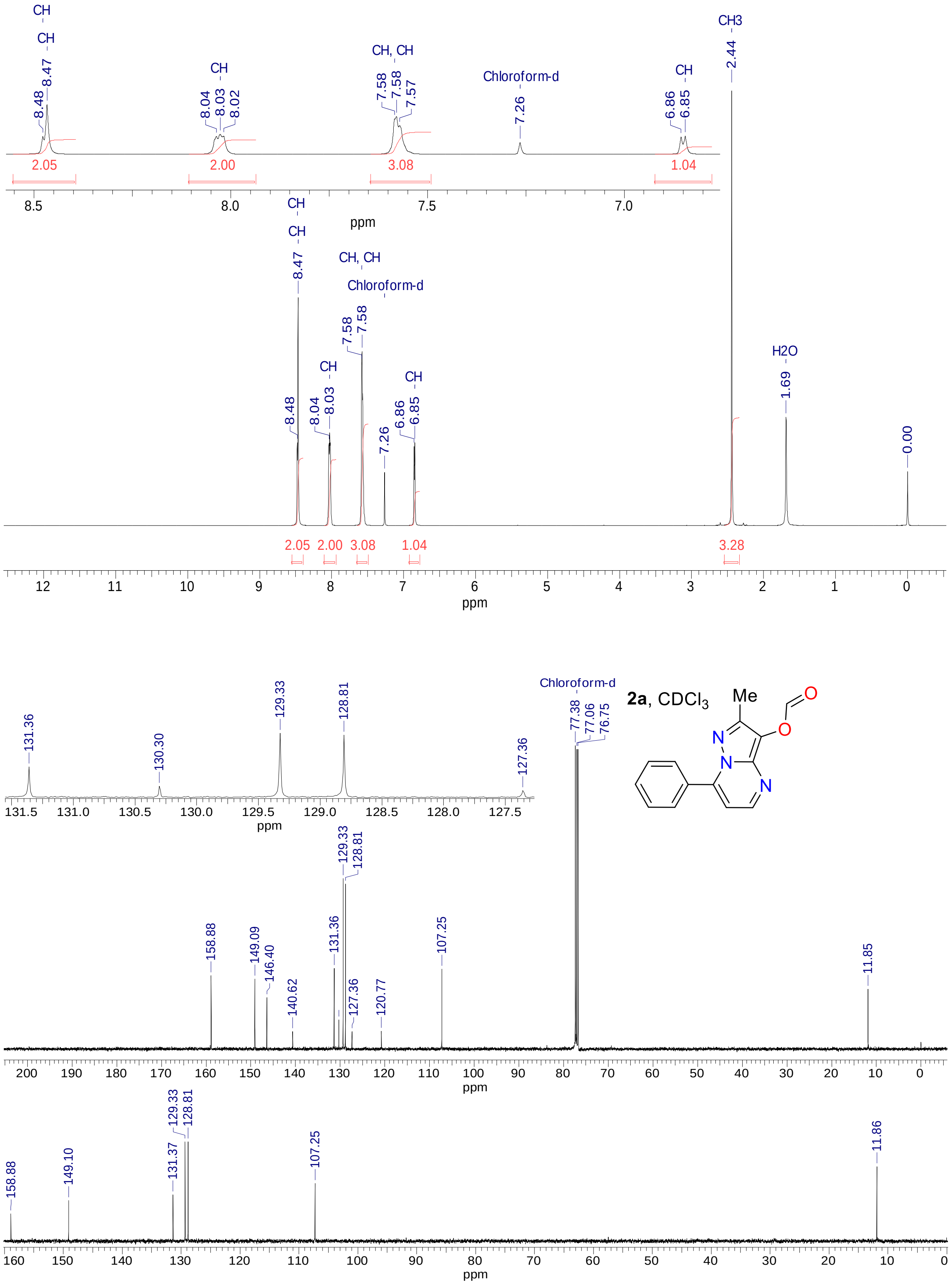

Fig. S40 ^{1}H/^{13}C NMR and DEPT-135 spectra of 2-methyl-7-phenylpyrazolo[1,5-*a*]pyrimidin-3-yl formate (**2a**).

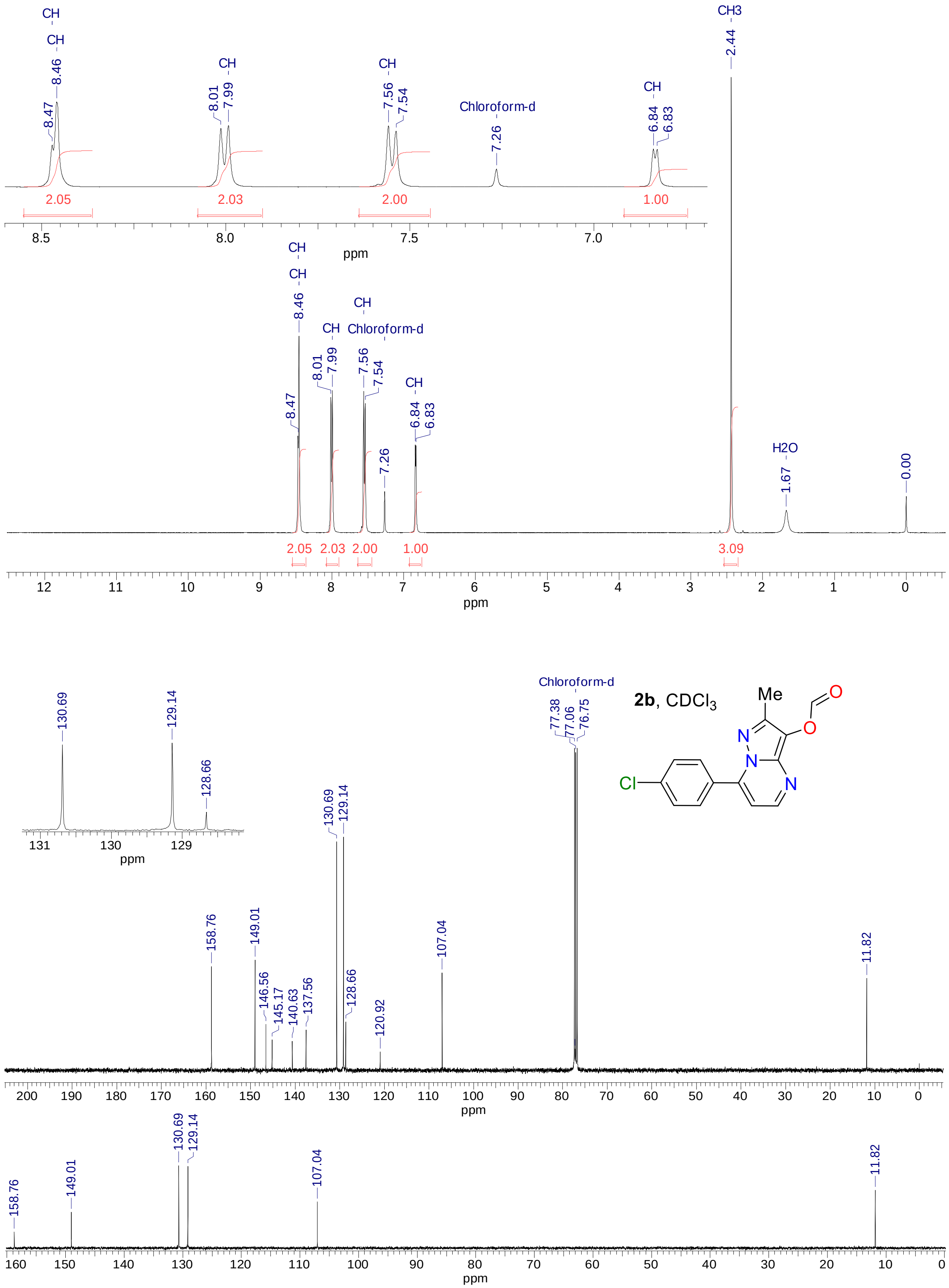

Fig. S41 $^{1}H/^{13}C$ NMR and DEPT-135 spectra of 7-(4-chlorophenyl)-2-methyl-Pp-3-yl formate (**2b**).

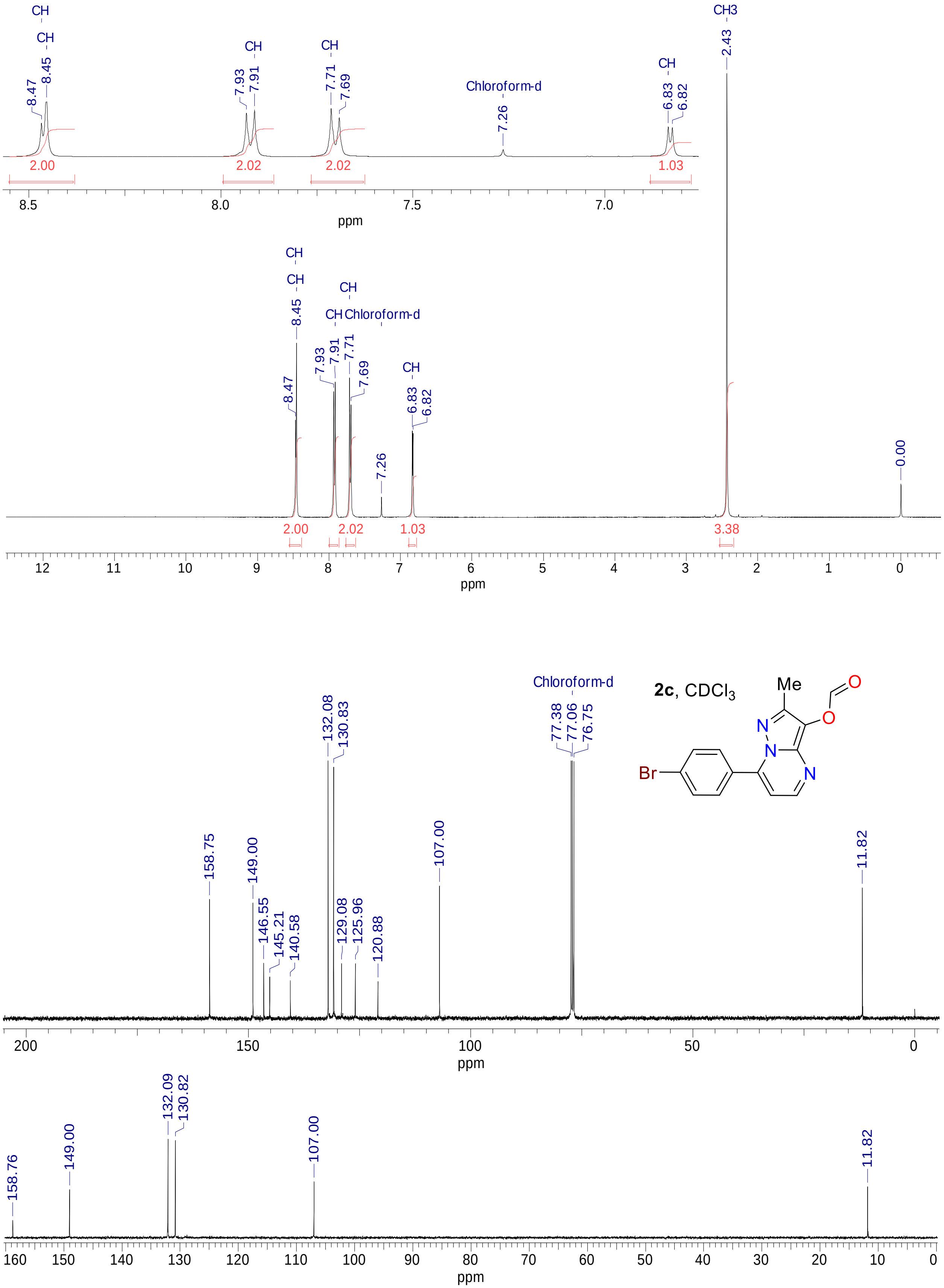

Fig. S42 $^{1}H/^{13}C$ NMR and DEPT-135 spectra of 7-(4-bromophenyl)-2-methyl-Pp-3-yl formate (**2c**).

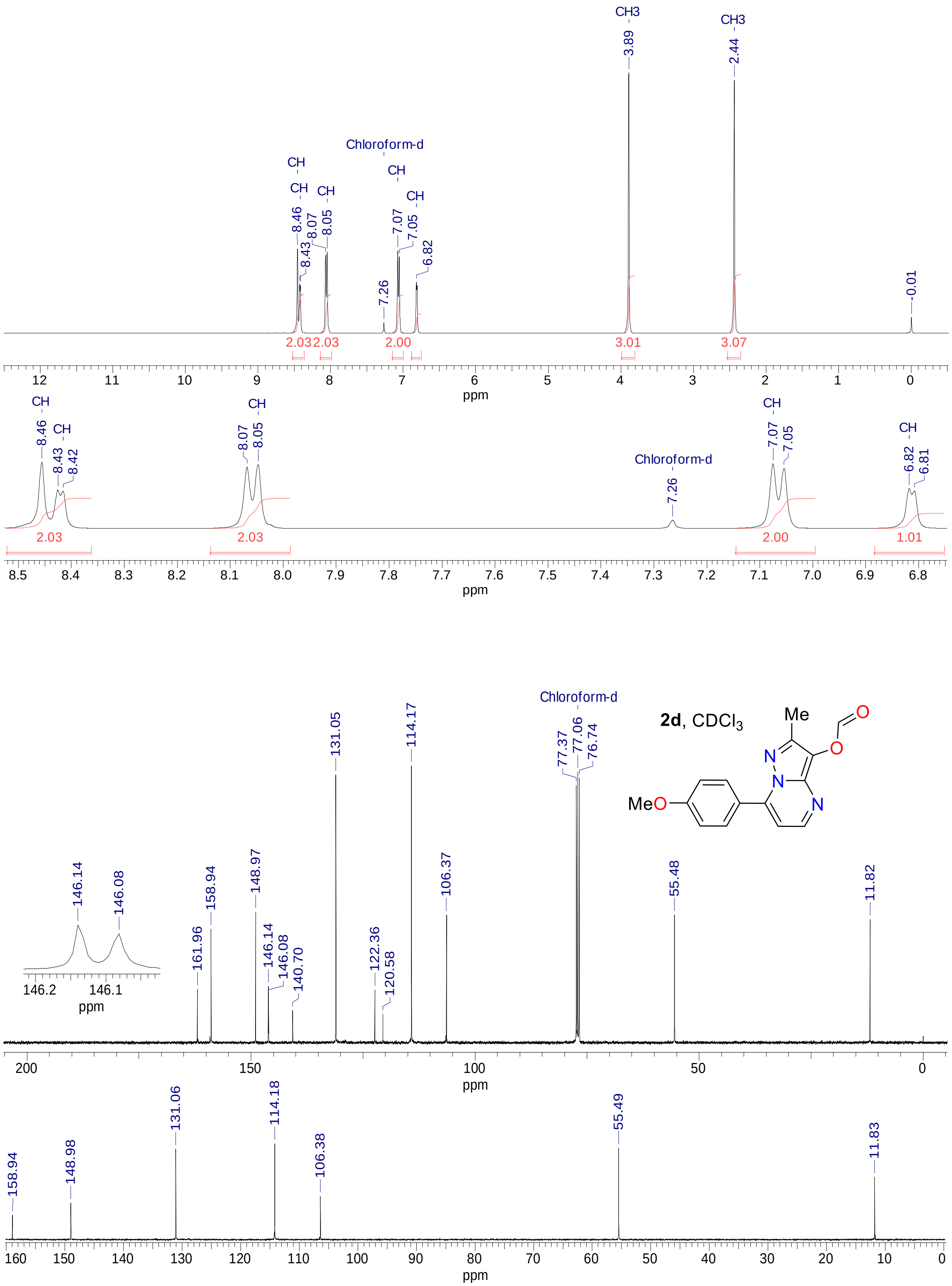

Fig. S43 $^{1}H/^{13}C$ NMR and DEPT-135 spectra of 7-(4-methoxyphenyl)-2-methyl-Pp-3-yl formate (**2d**).

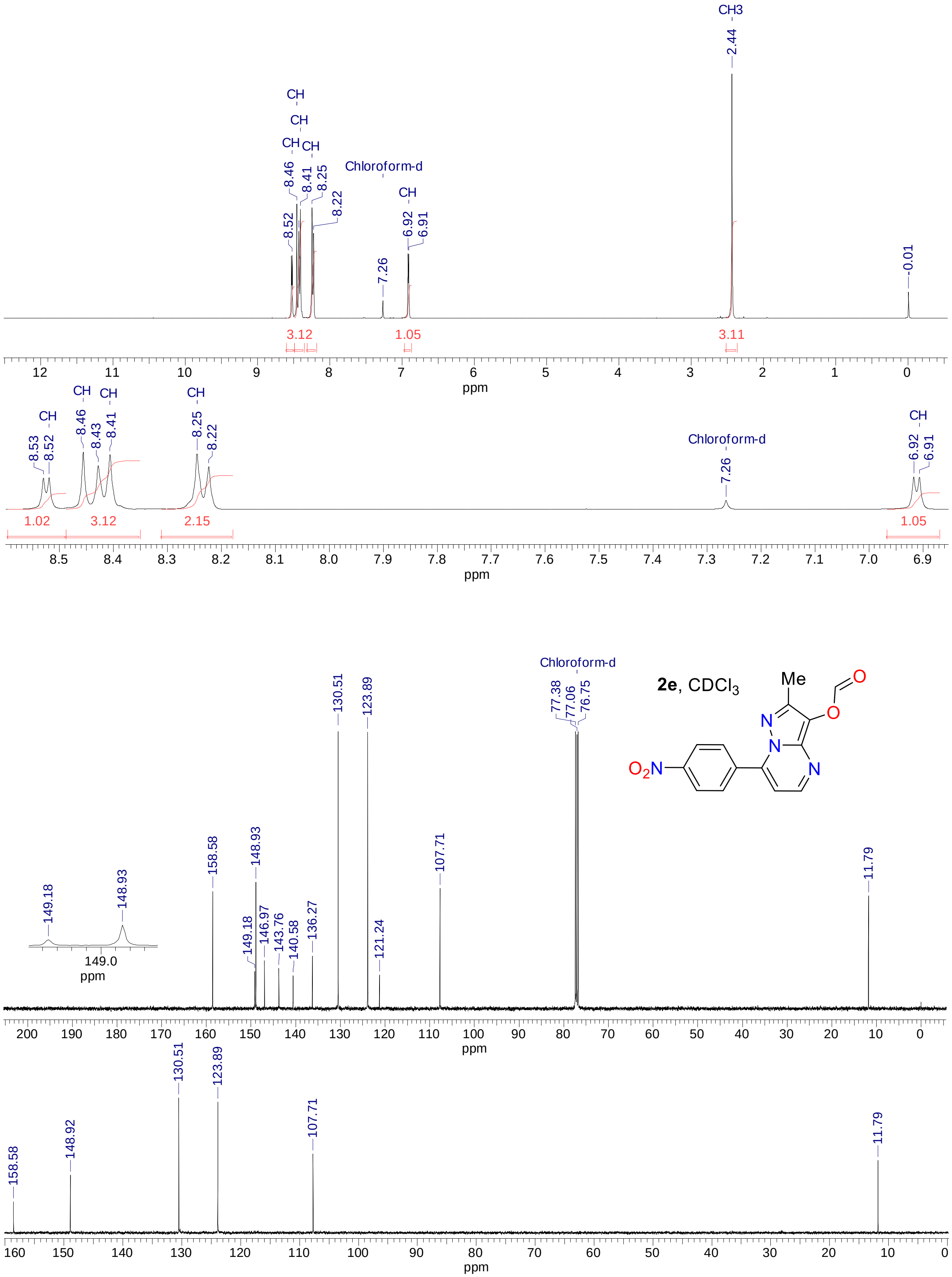

Fig. S44 ^{1}H/^{13}C NMR and DEPT-135 spectra of 2-methyl-7-(4-nitrophenyl)-Pp-3-yl formate (**2e**).

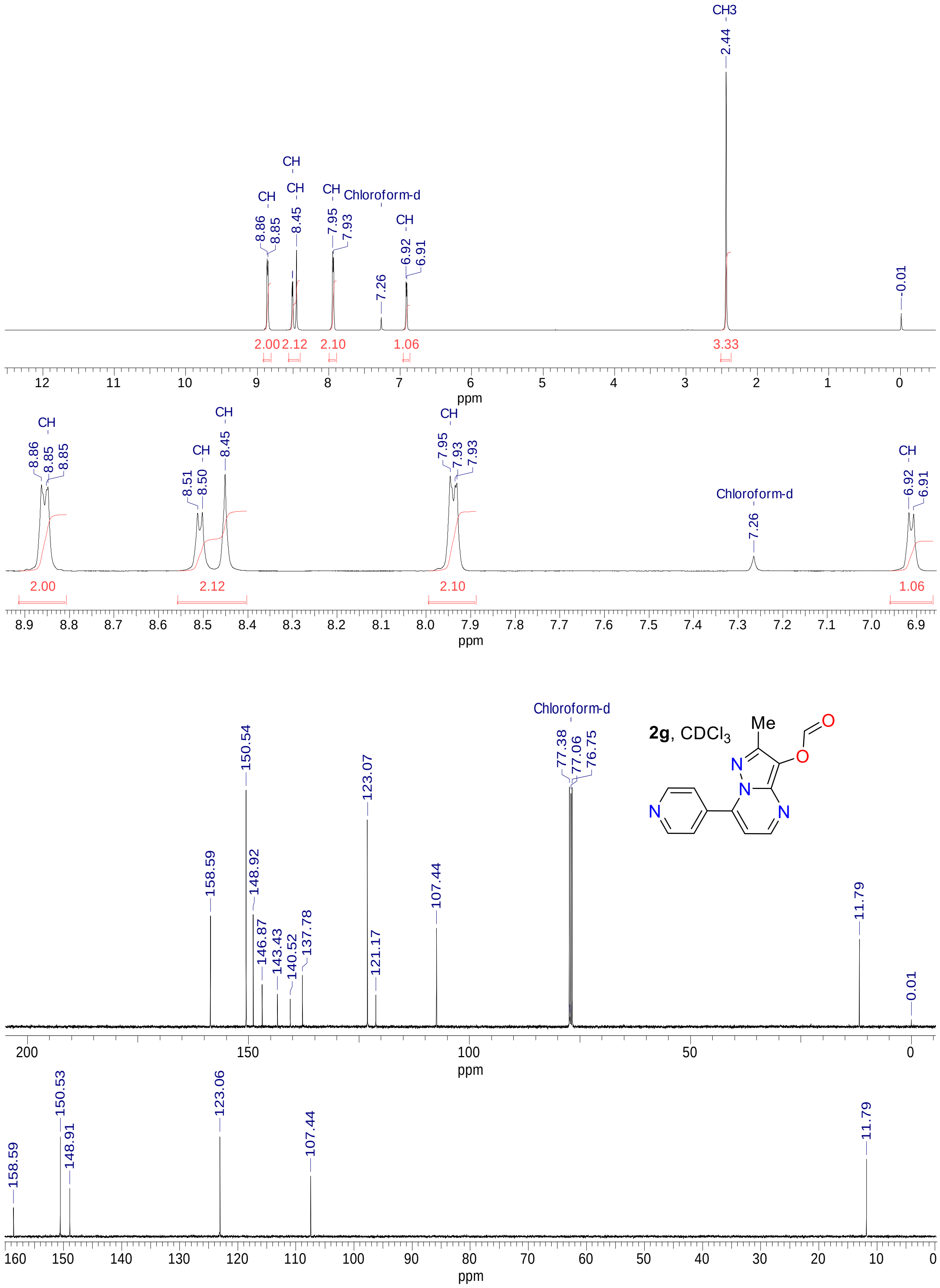

Fig. S45 $^{1}H/^{13}C$ NMR and DEPT-135 spectra of 2-methyl-7-g(4-pyridyl)-Pp-3-yl formate (**2g**).

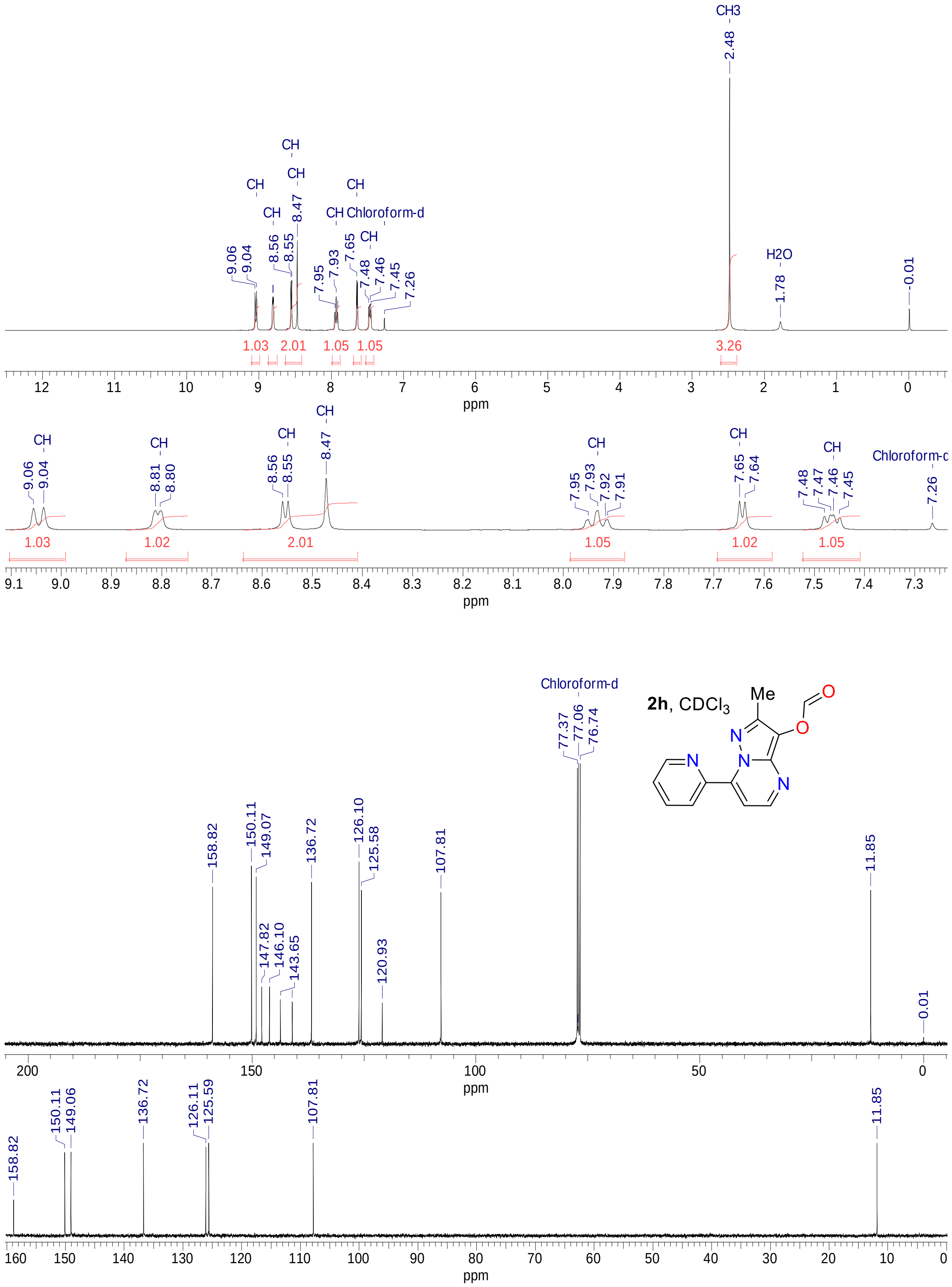

Fig. S46 $^{1}H/^{13}C$ NMR and DEPT-135 spectra of 2-methyl-7-(2-pyridyl)-Pp-3-yl formate (**2h**).

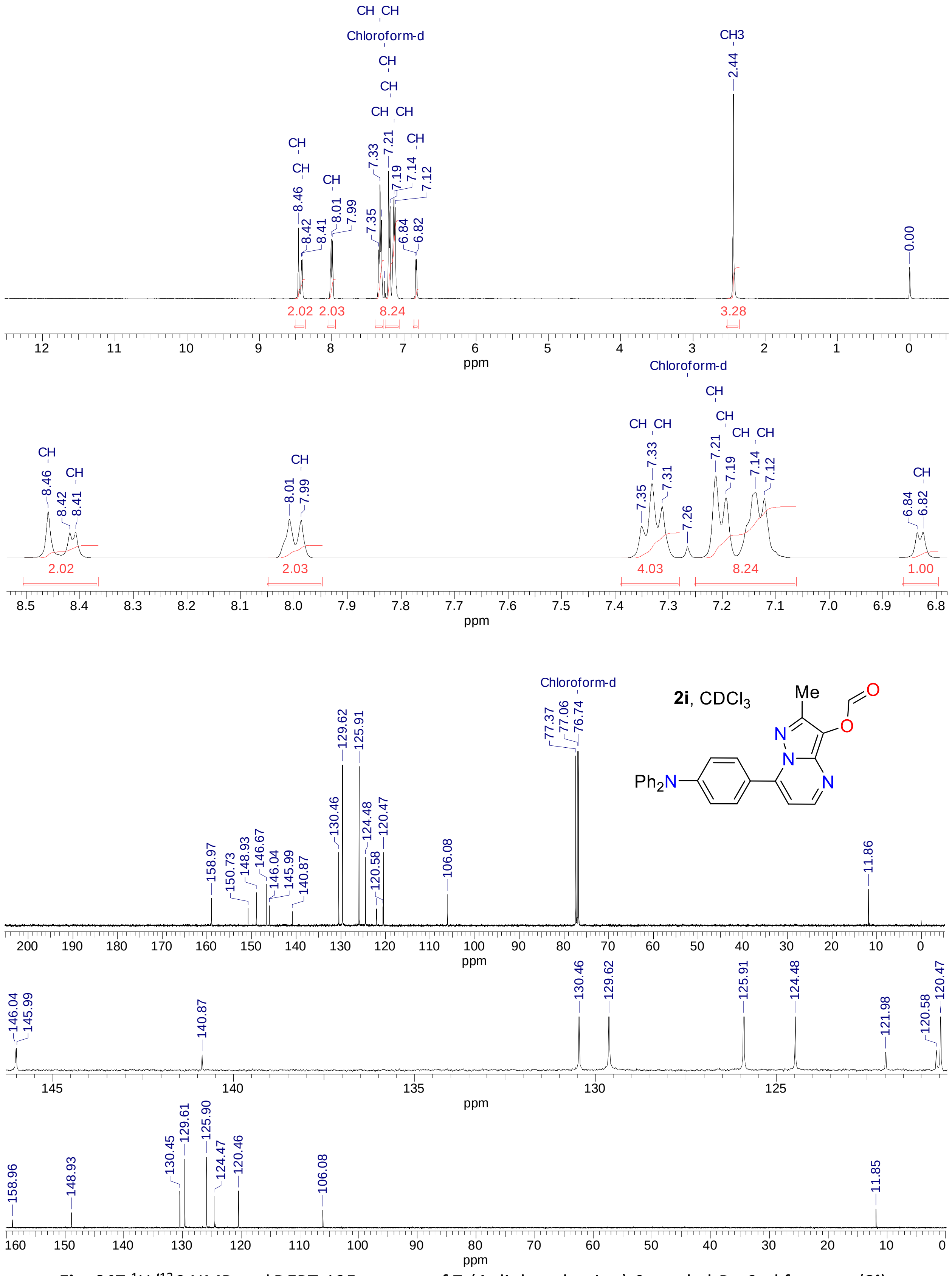

Fig. S47 $^{1}H/^{13}C$ NMR and DEPT-135 spectra of 7-(4-diphenylamino)-2-methyl-Pp-3-yl formate (**2i**).

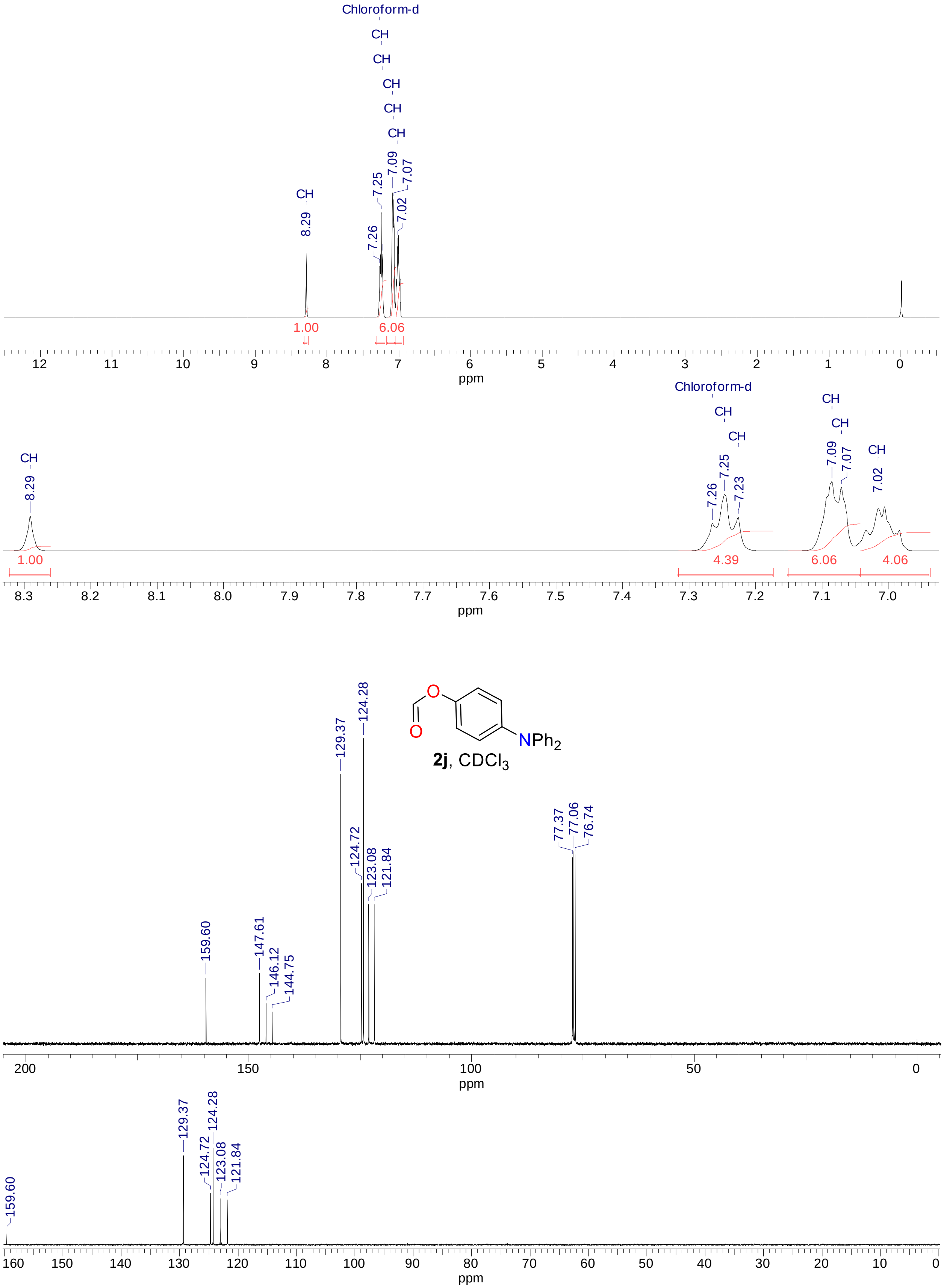

Fig. S48 ^{1}H/^{13}C NMR and DEPT-135 spectra of 4-diphenylaminophenyl formate (**2j**).

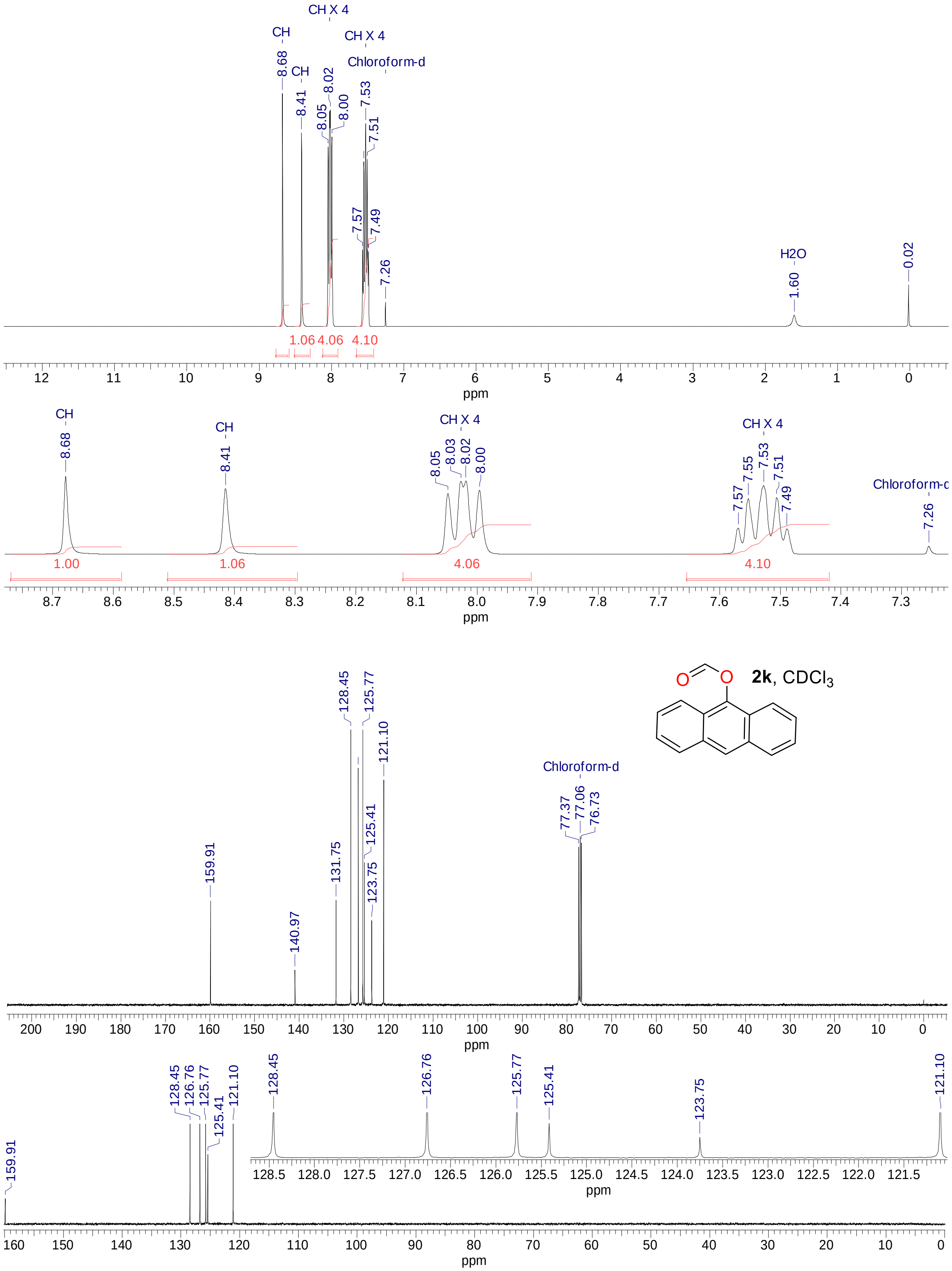

Fig. S49 ^{1}H/^{13}C NMR and DEPT-135 spectra of anthracen-9-yl formate (**2k**).

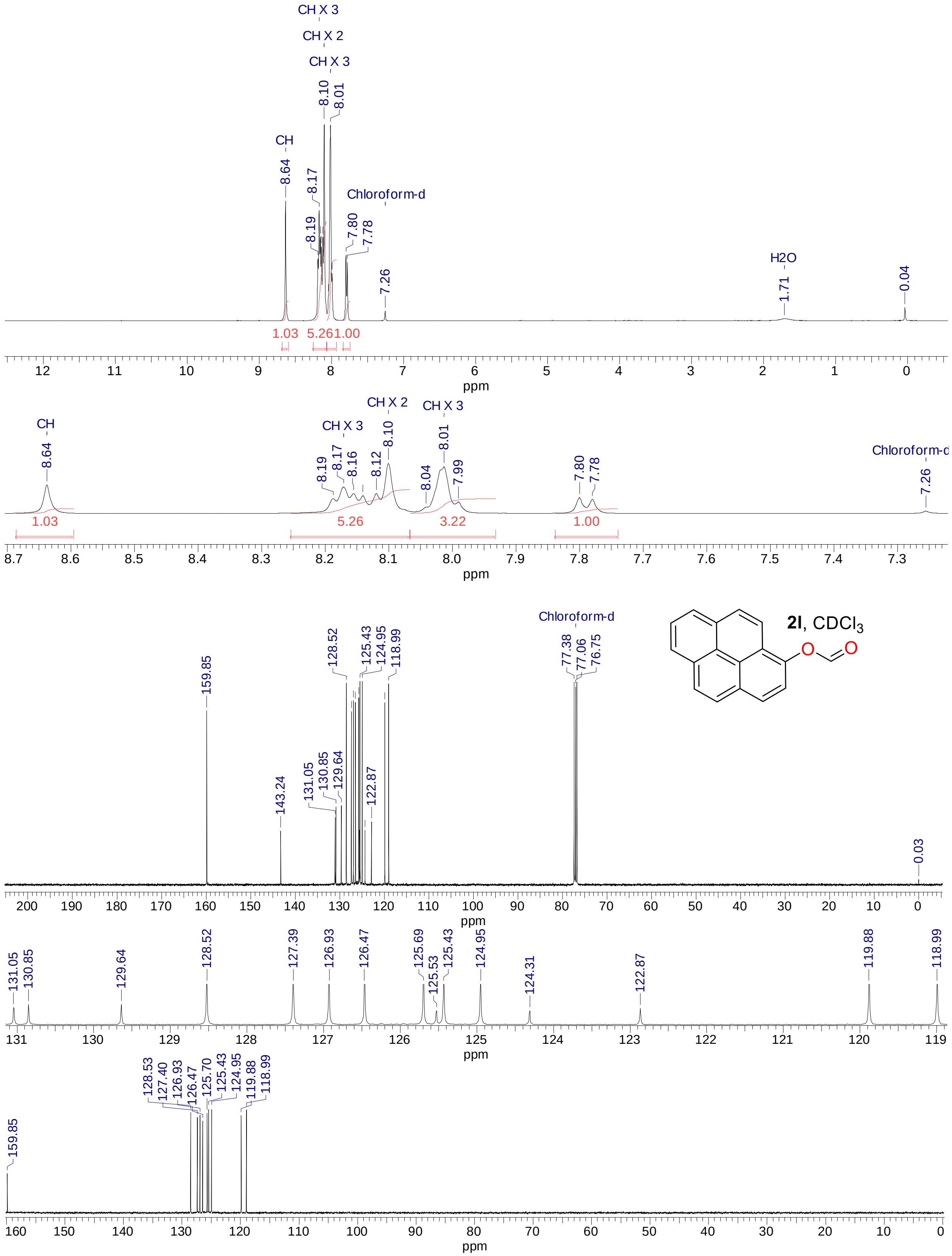

Fig. S50 $^{1}H/^{13}C$ NMR and DEPT-135 spectra of pyren-1-yl formate (**2l**).

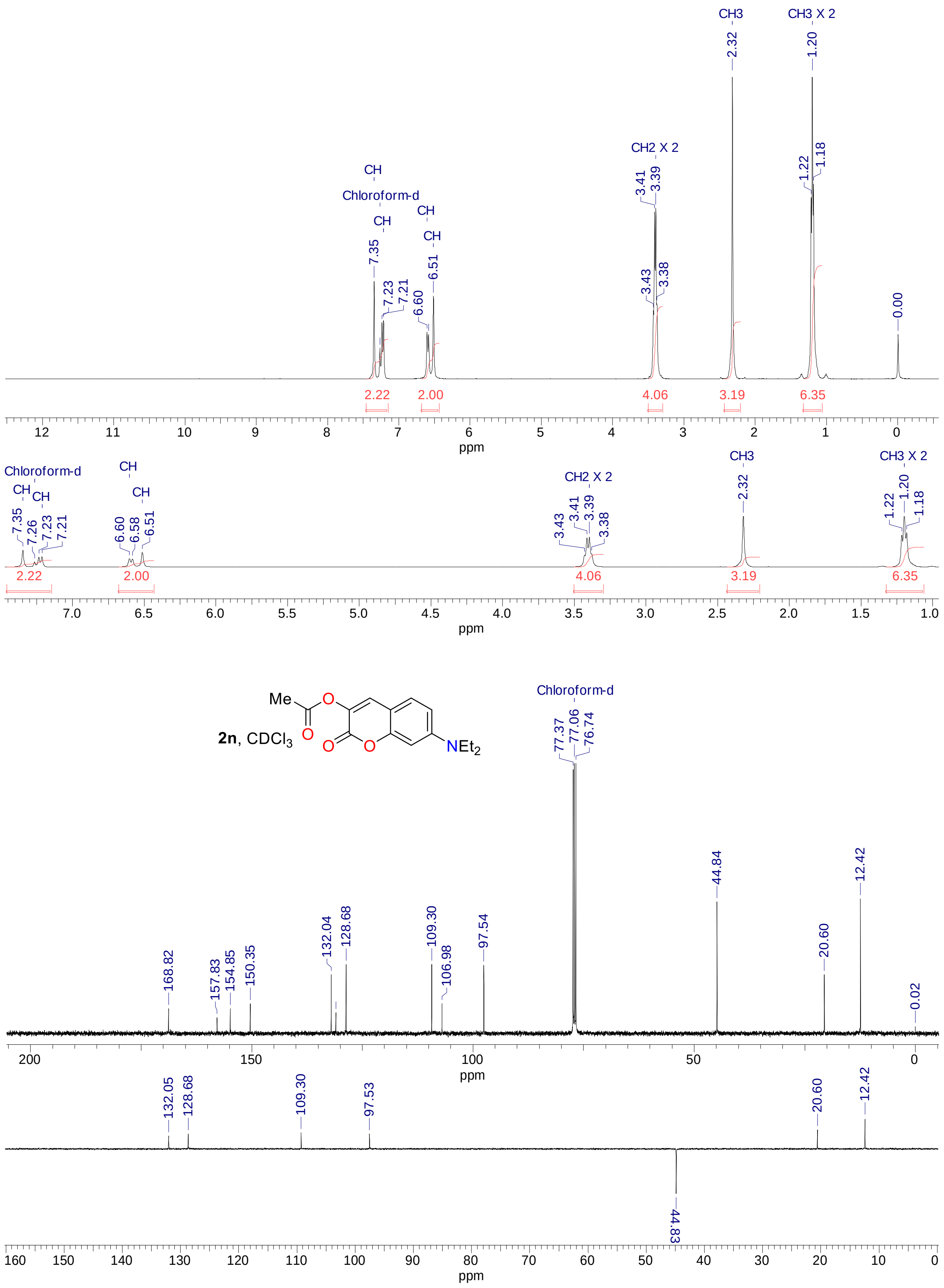

Fig. S51 $^{1}H/^{13}C$ NMR and DEPT-135 spectra of 7-(diethylaminocoumarin-3-yl acetate (**2n**).

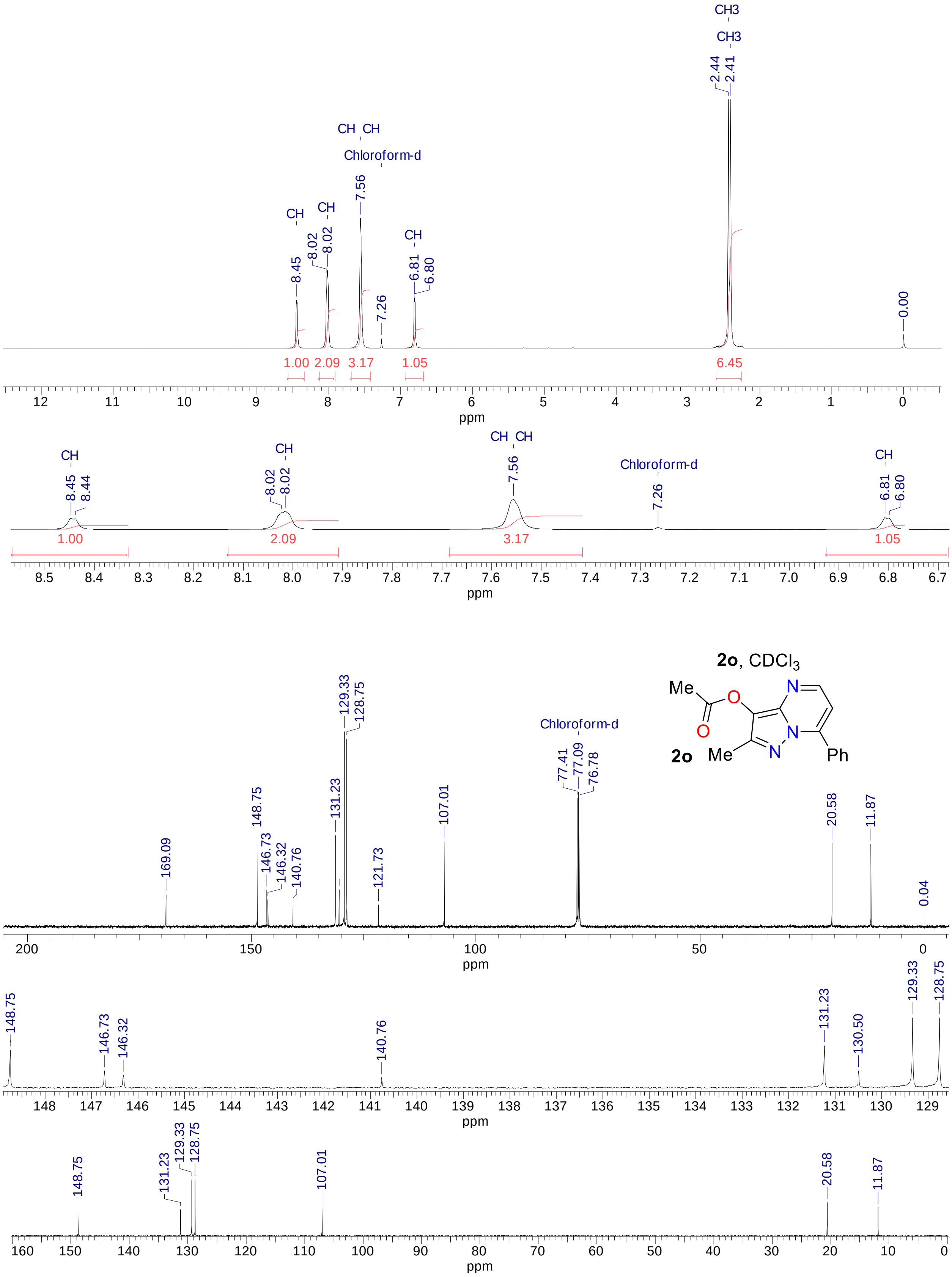

Fig. S52 $^{1}H/^{13}C$ NMR and DEPT-135 spectra of 2-methyl-7-phenyl-Pp-3-yl acetate (**2o**).

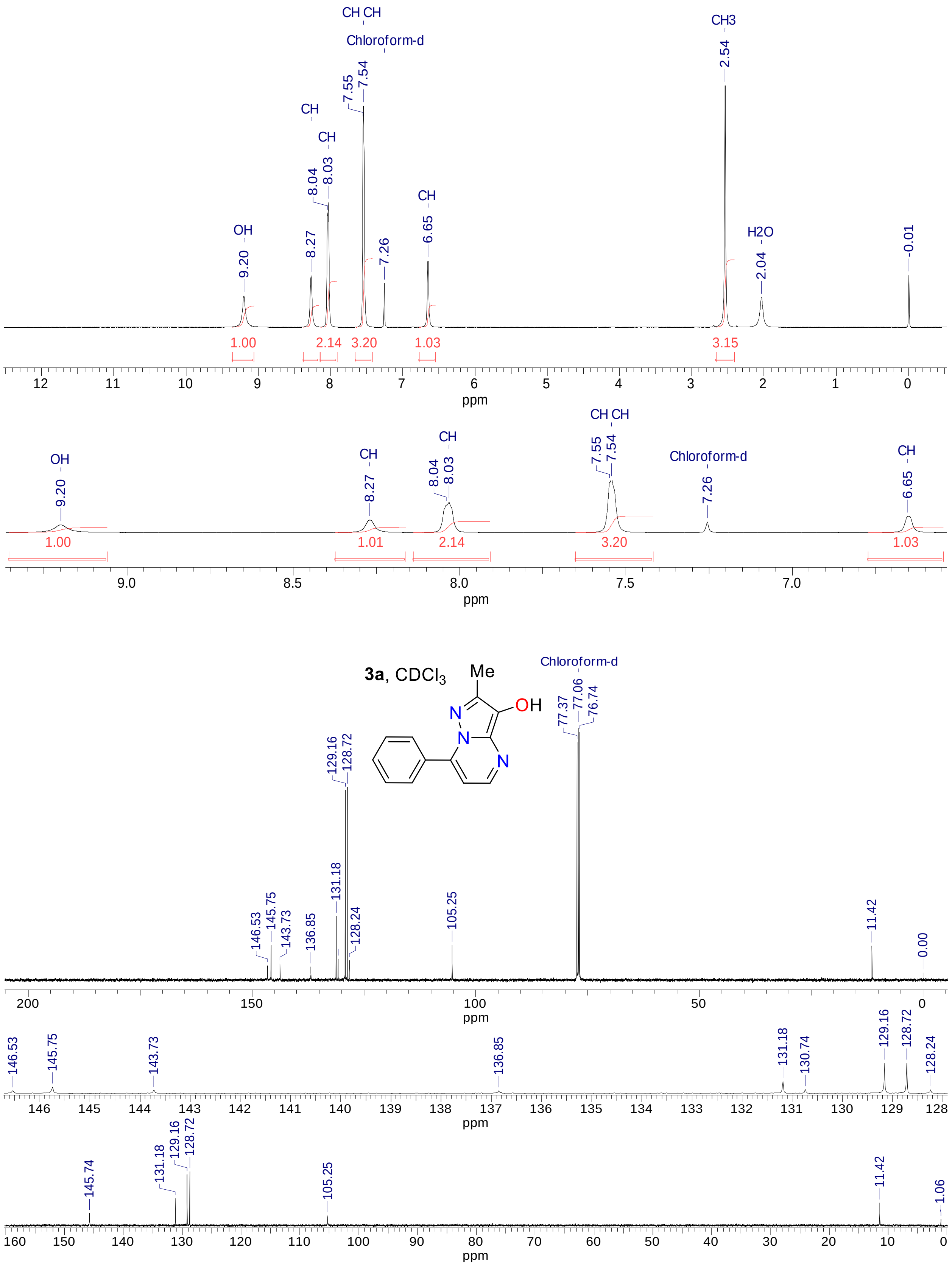

Fig. S53 $^{1}H/^{13}C$ NMR and DEPT-135 spectra of 2-methyl-7-phenylpyrazolo[1,5-*a*]pyrimidin-3-ol (**3a**).

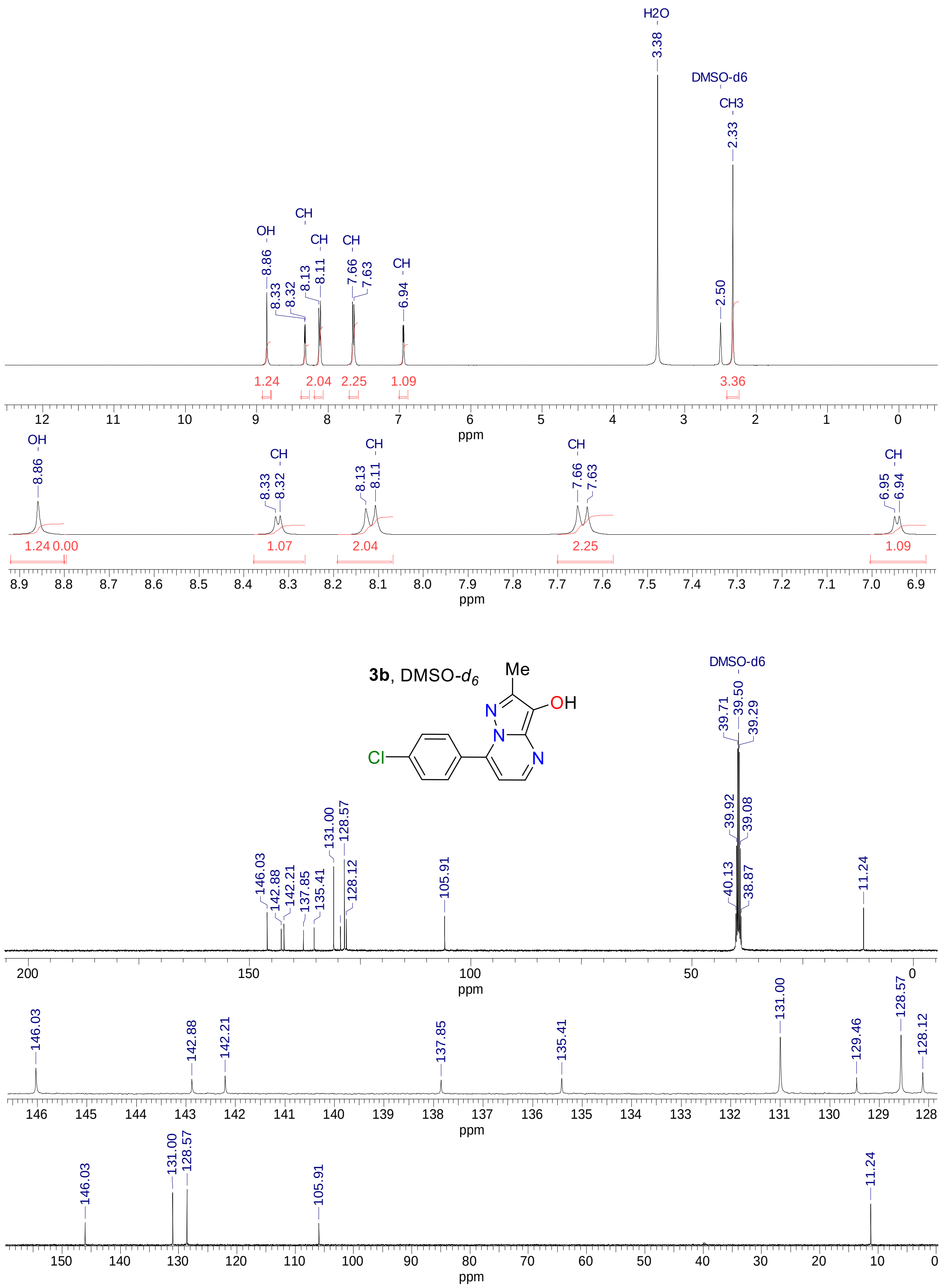

Fig. S54 ^{1}H/^{13}C NMR and DEPT-135 spectra of 7-(4-chlorophenyl)-2-methyl-Pp-3-ol (**3b**).

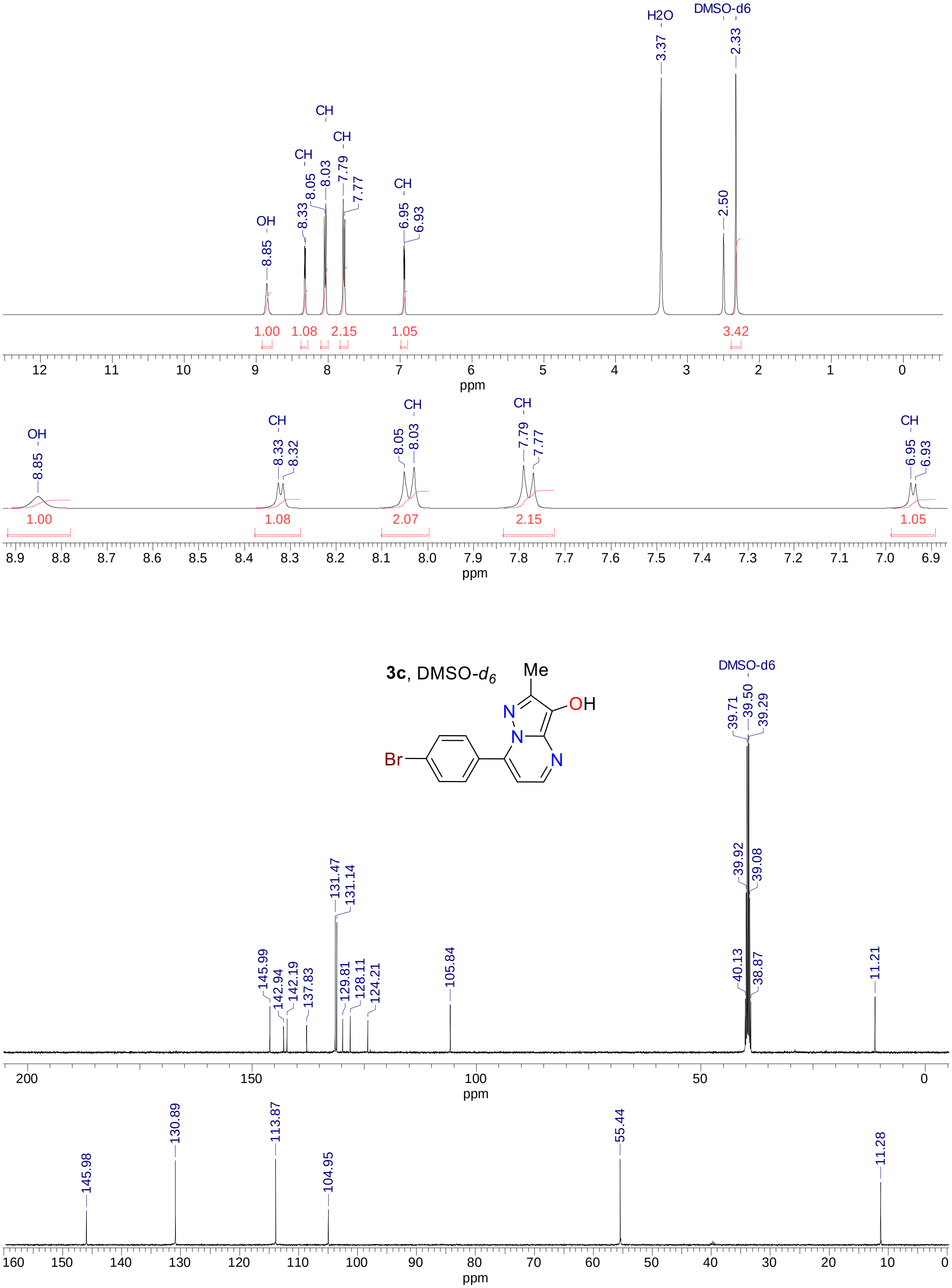

Fig. S55 $^{1}H/^{13}C$ NMR and DEPT-135 spectra of 7-(4-bromophenyl)-2-methyl-Pp-3-ol (**3c**).

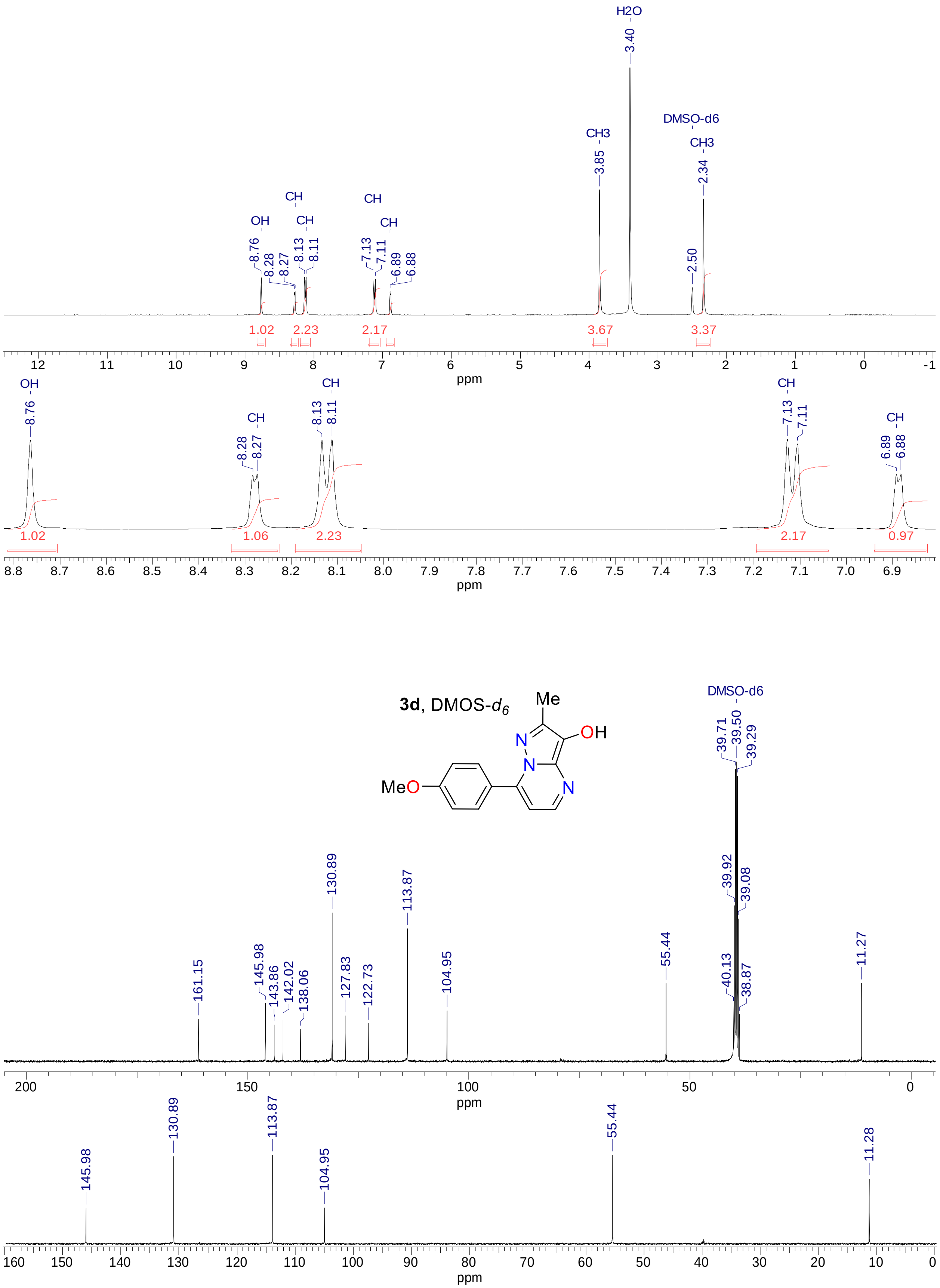

Fig. S56 $^{1}H/^{13}C$ NMR and DEPT-135 spectra of 7-(4-methoxyphenyl)-2-methyl-Pp-3-ol (**3d**).

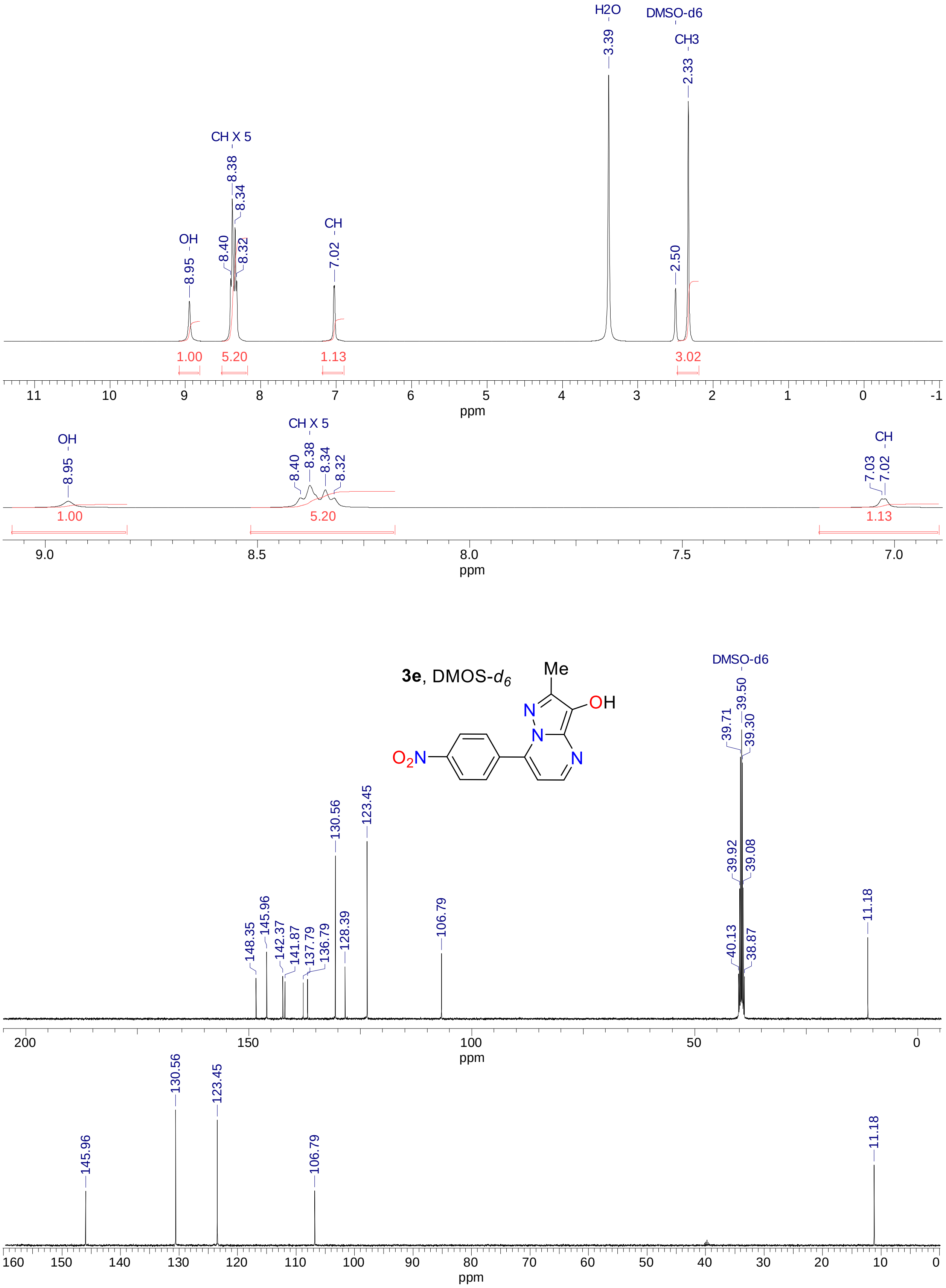

Fig. S57 $^{1}H/^{13}C$ NMR and DEPT-135 spectra of 2-methyl-7-(4-nitrophenyl)-Pp-3ol (**3e**).

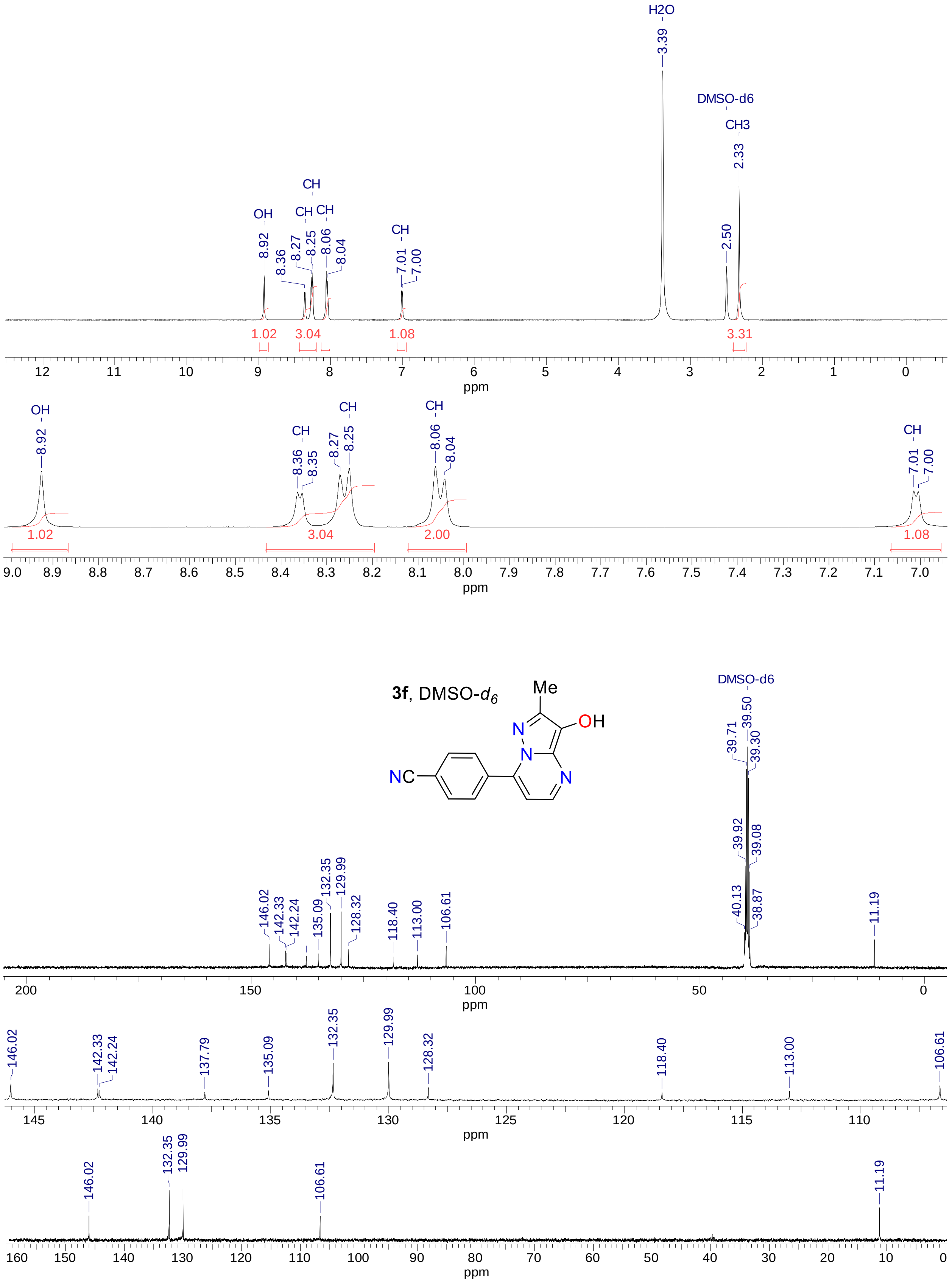

Fig. S58 $^{1}H/^{13}C$ NMR and DEPT-135 spectra of 2-methyl-7-(4-cyanophenyl)-Pp-3ol (**3f**).

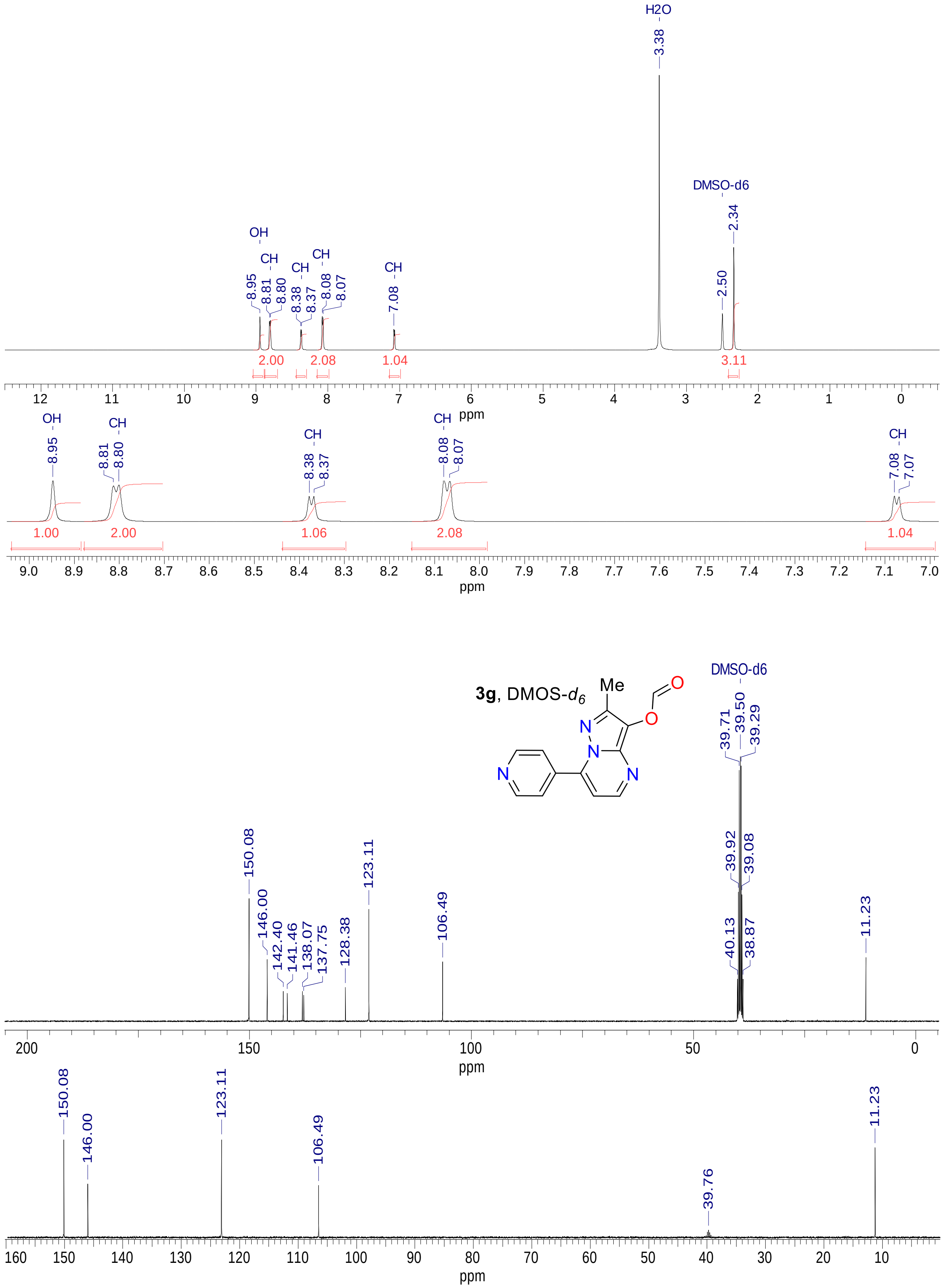

Fig. S59 $^{1}H/^{13}C$ NMR and DEPT-135 spectra of 2-methyl-7-(4-pyridyl)-Pp-3-ol (**3g**).

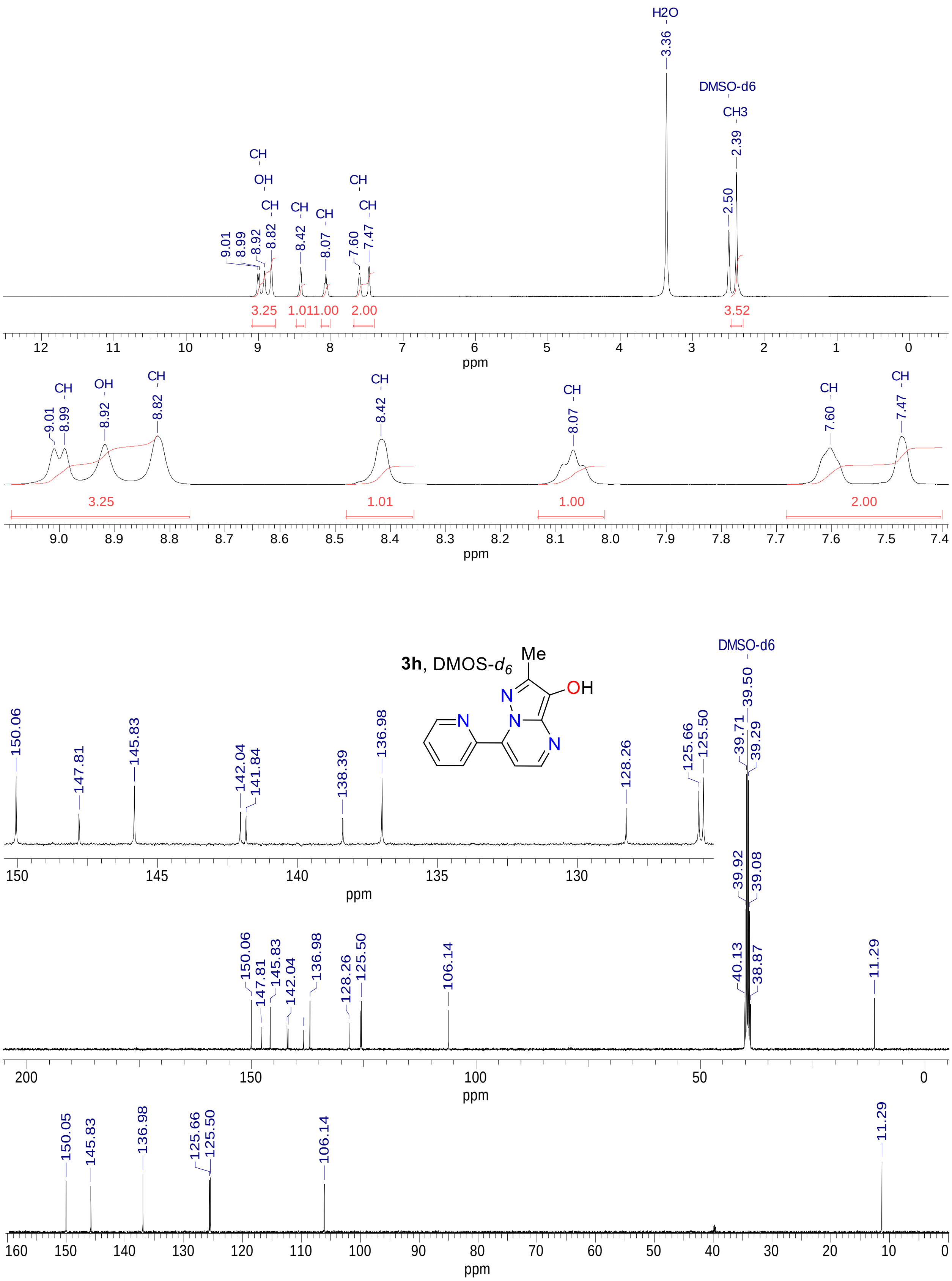

Fig. S60 $^{1}H/^{13}C$ NMR and DEPT-135 spectra of 2-methyl-7-(2-pyridyl)-Pp-3-ol (**3h**).

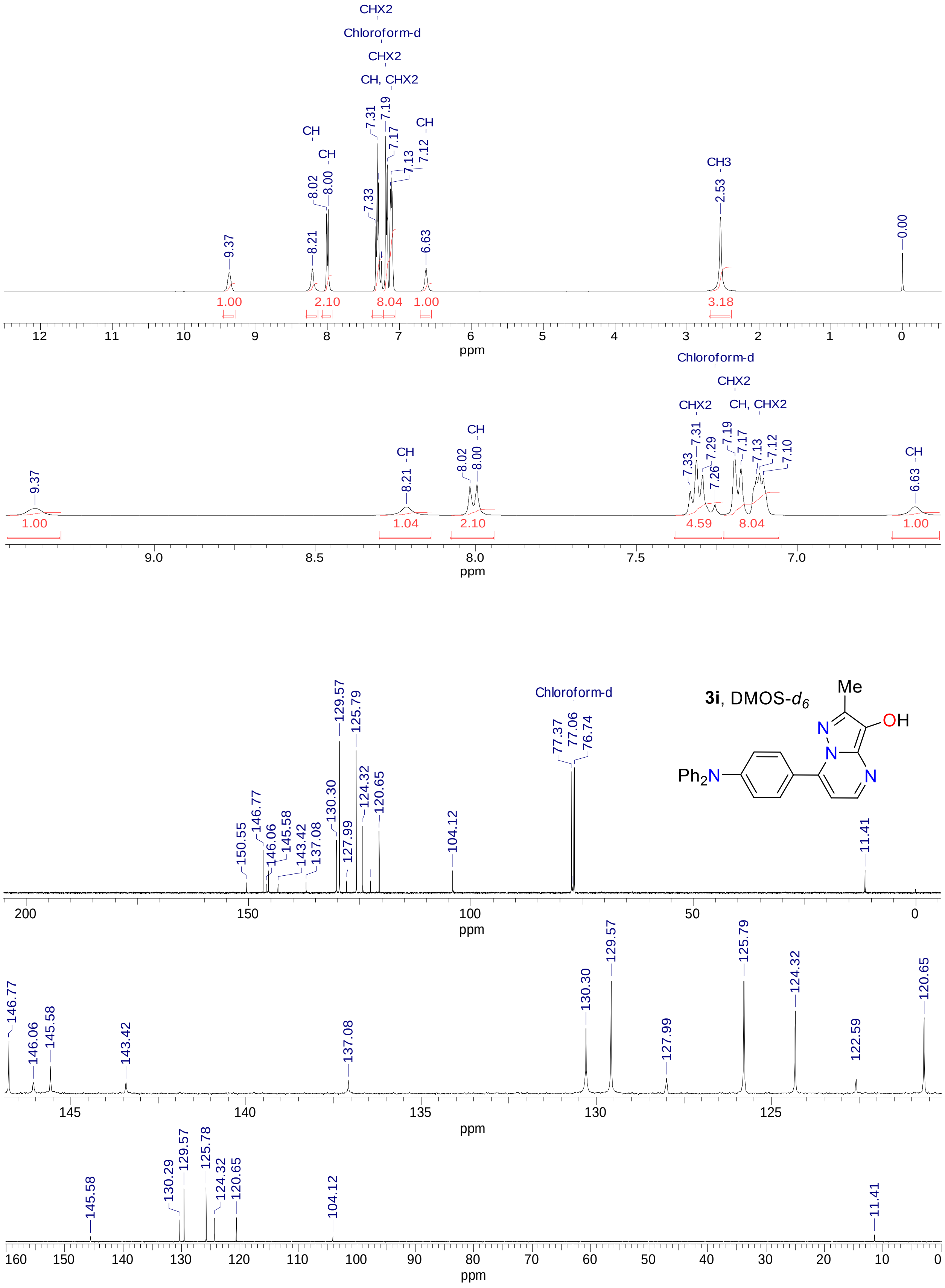

Fig. S61 $^{1}H/^{13}C$ NMR and DEPT-135 spectra of 7-(4-diphenylamino)-2-methyl-Pp-3-ol (**3i**).

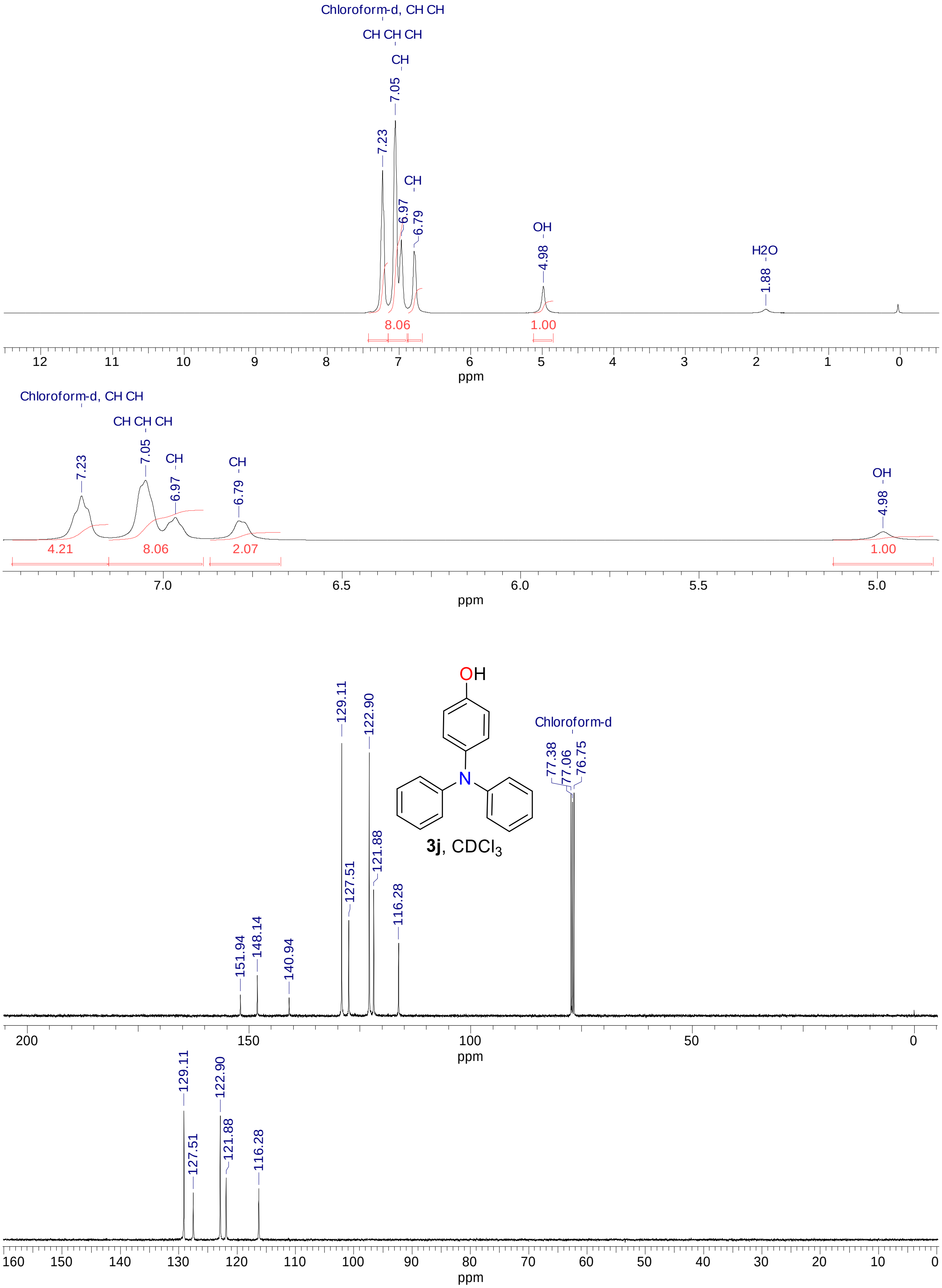

Fig. S62 $^{1}H/^{13}C$ NMR and DEPT-135 spectra of 4-diphenylaminophenol (**3j**).

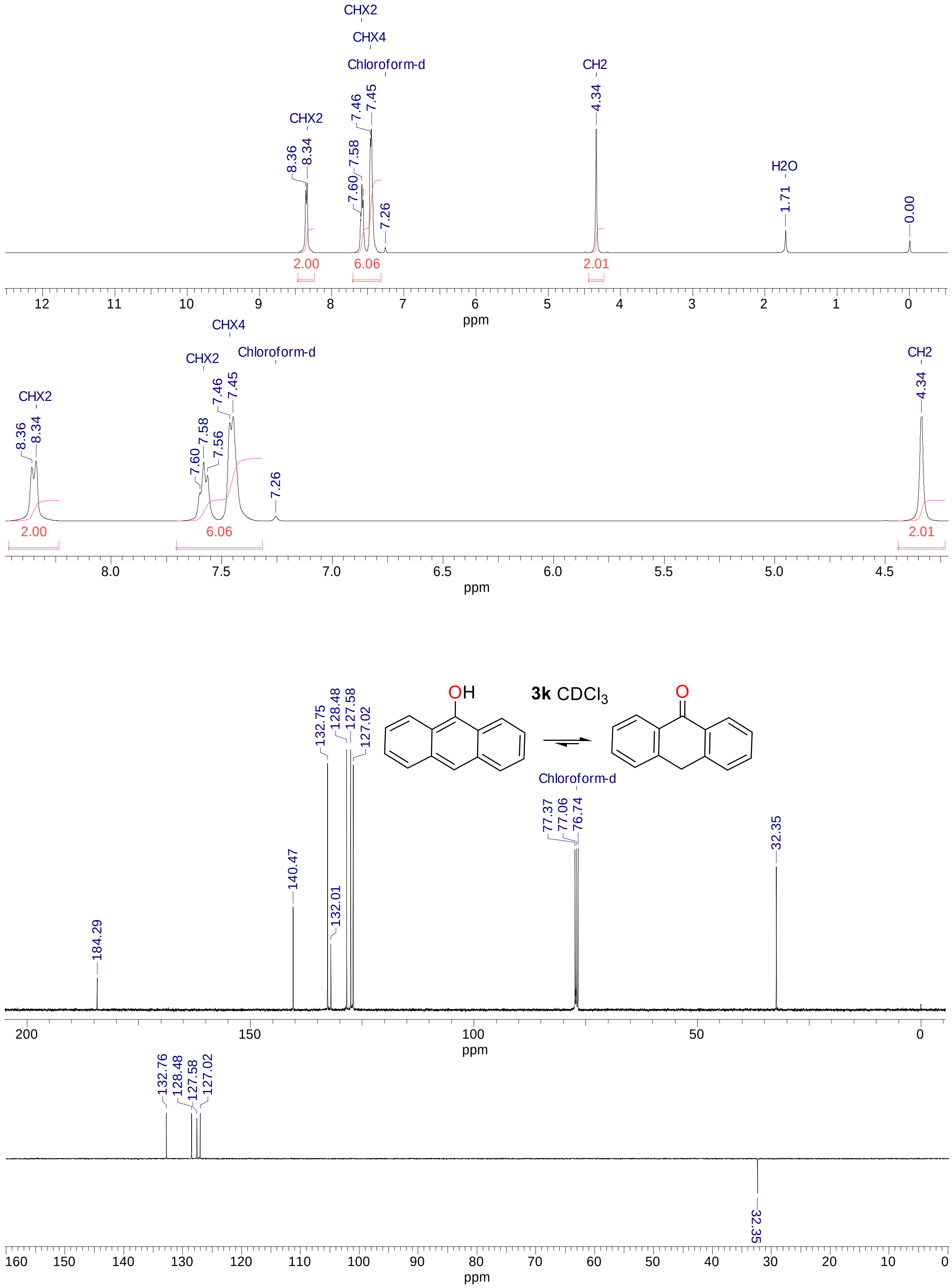

Fig. S63 $^{1}H/^{13}C$ NMR and DEPT-135 spectra of anthracen-9-ol (**3k**).

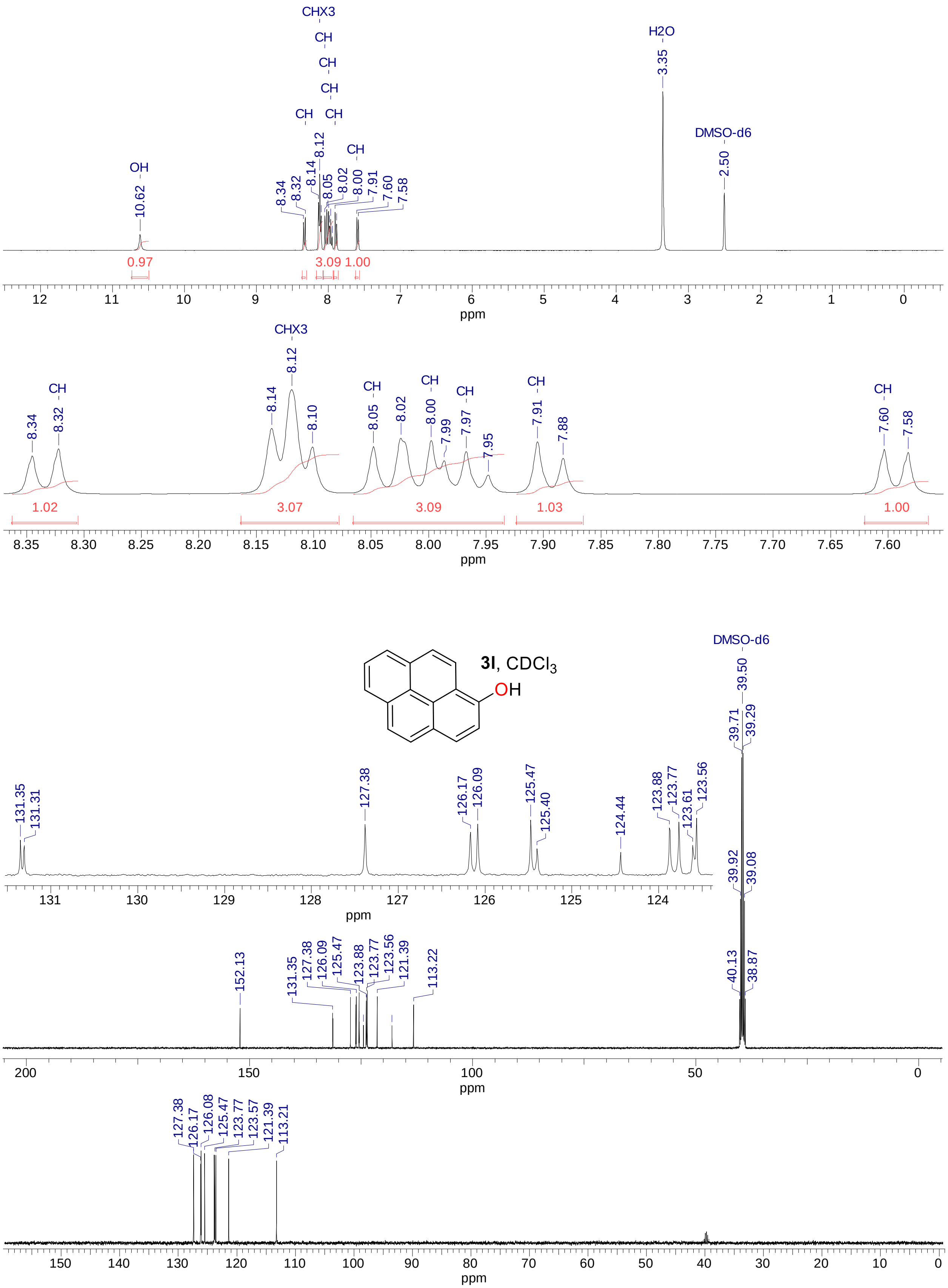

Fig. S64 ^{1}H/^{13}C NMR and DEPT-135 spectra of pyren-1-ol (**3l**).

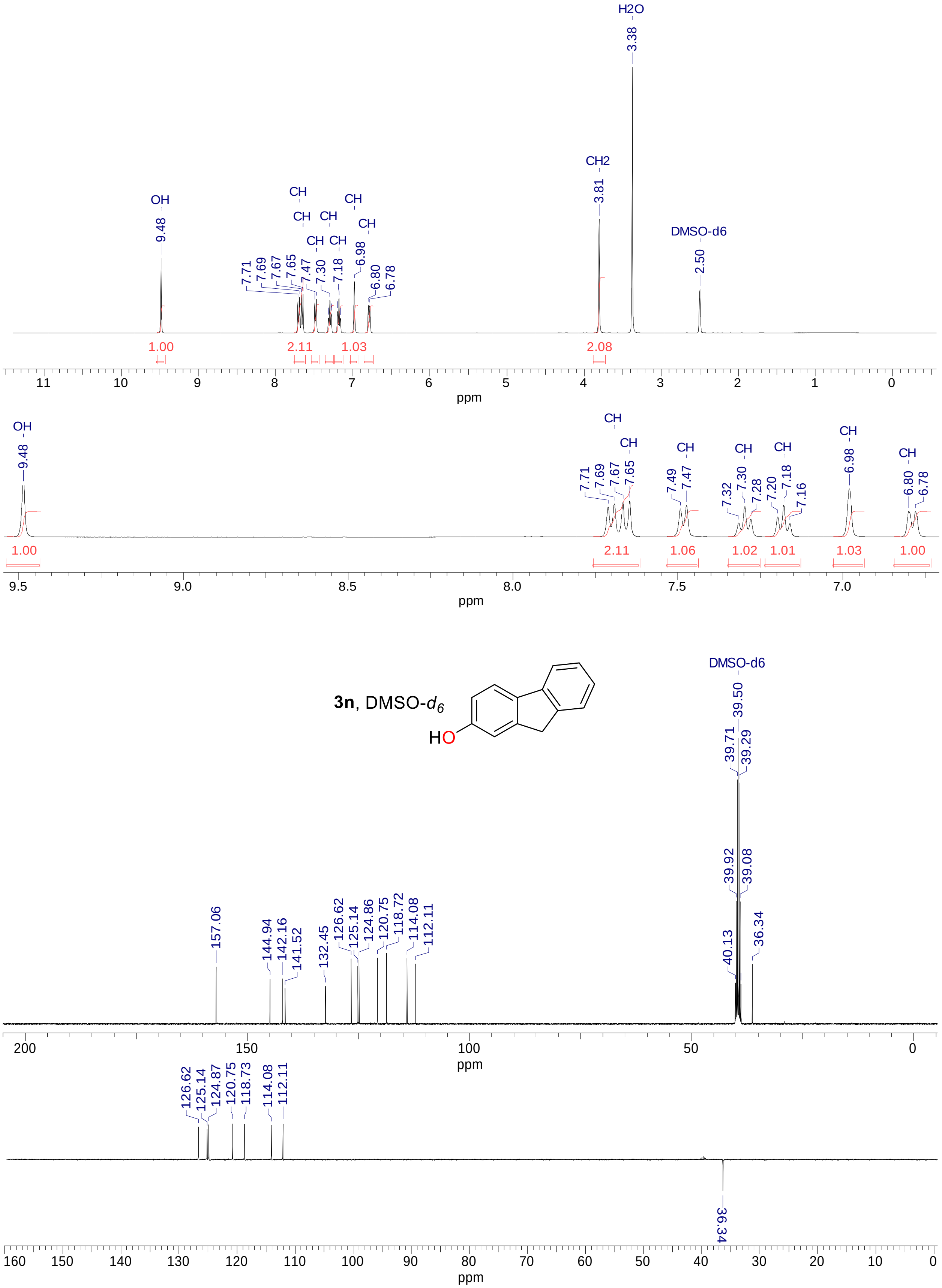

Fig. S65 $^1H/^{13}C$ NMR and DEPT-135 spectra of 9*H*-fluoren-2-ol (**3m**).

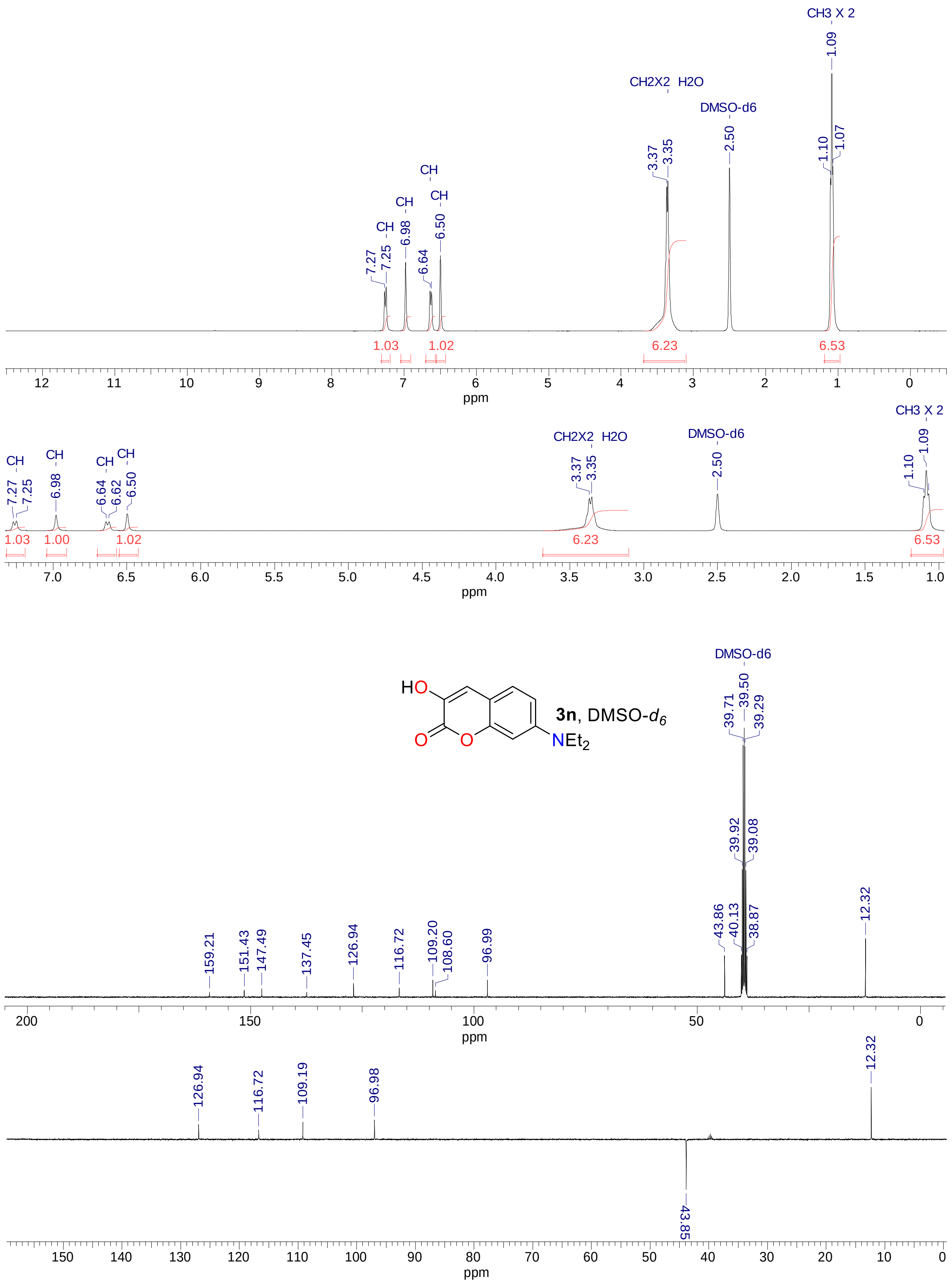

Fig. S66 ^{1}H/^{13}C NMR and DEPT-135 spectra of 7-diethylaminocoumarin-3-ol (**3n**).

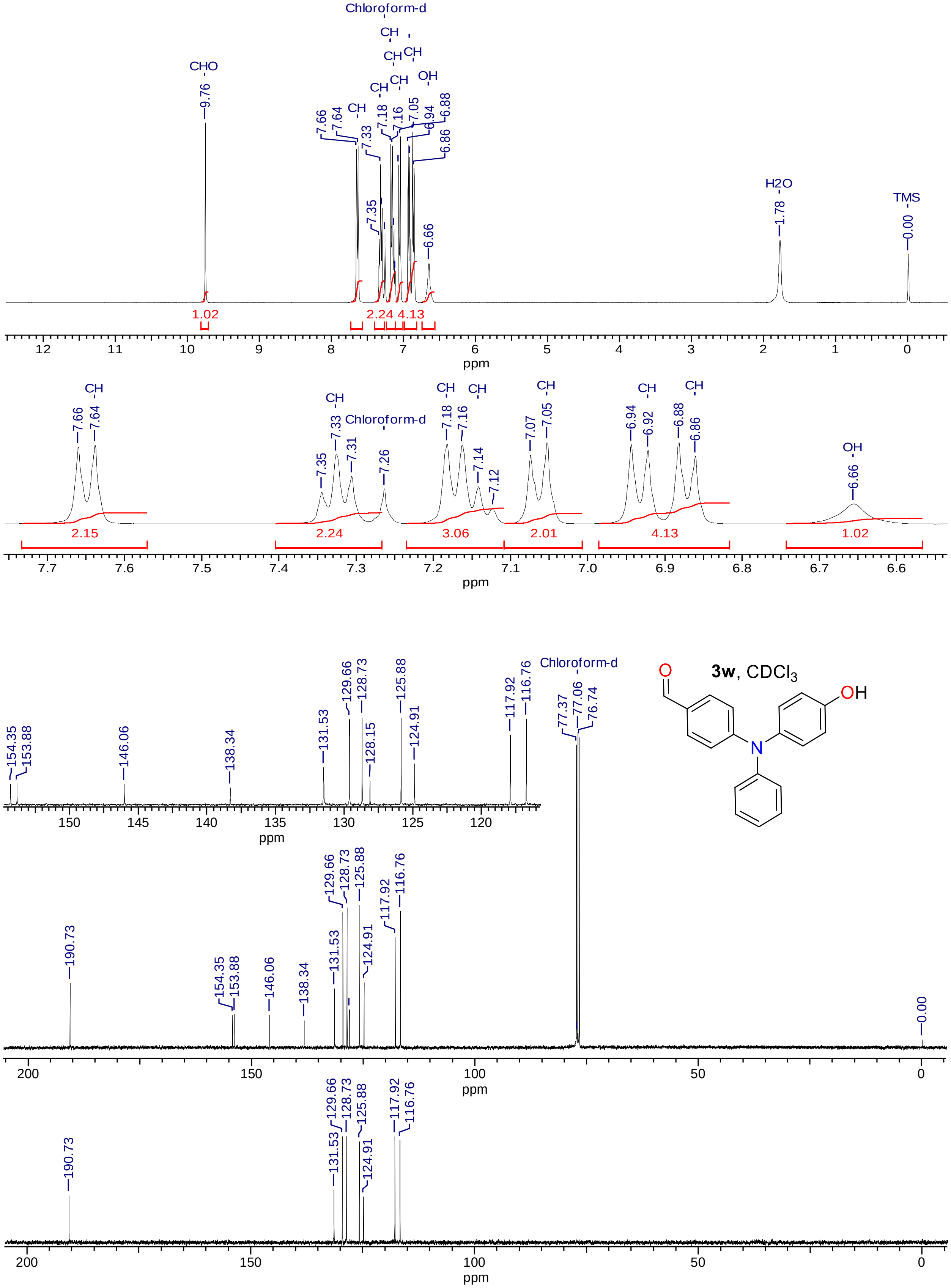

Fig. S67 $^{1}H/^{13}C$ NMR and DEPT-135 spectra of 4-((4-hydroxyphenyl)(phenyl)amino)benzaldehyde (**3w**).

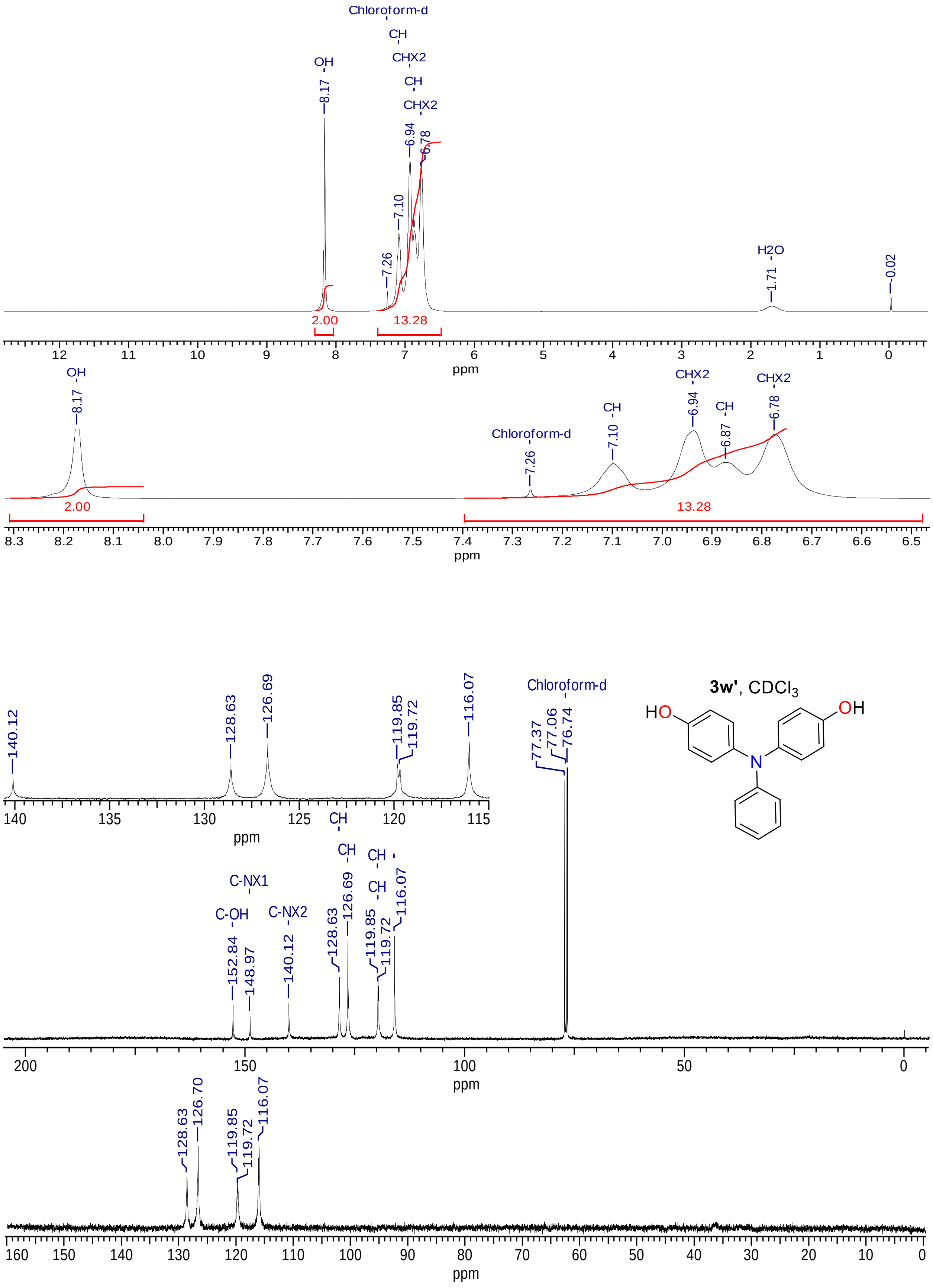

Fig. S68 $^{1}H/^{13}C$ NMR and DEPT-135 spectra of 4,4'-(phenylazanediyl)diphenol (**3w'**).

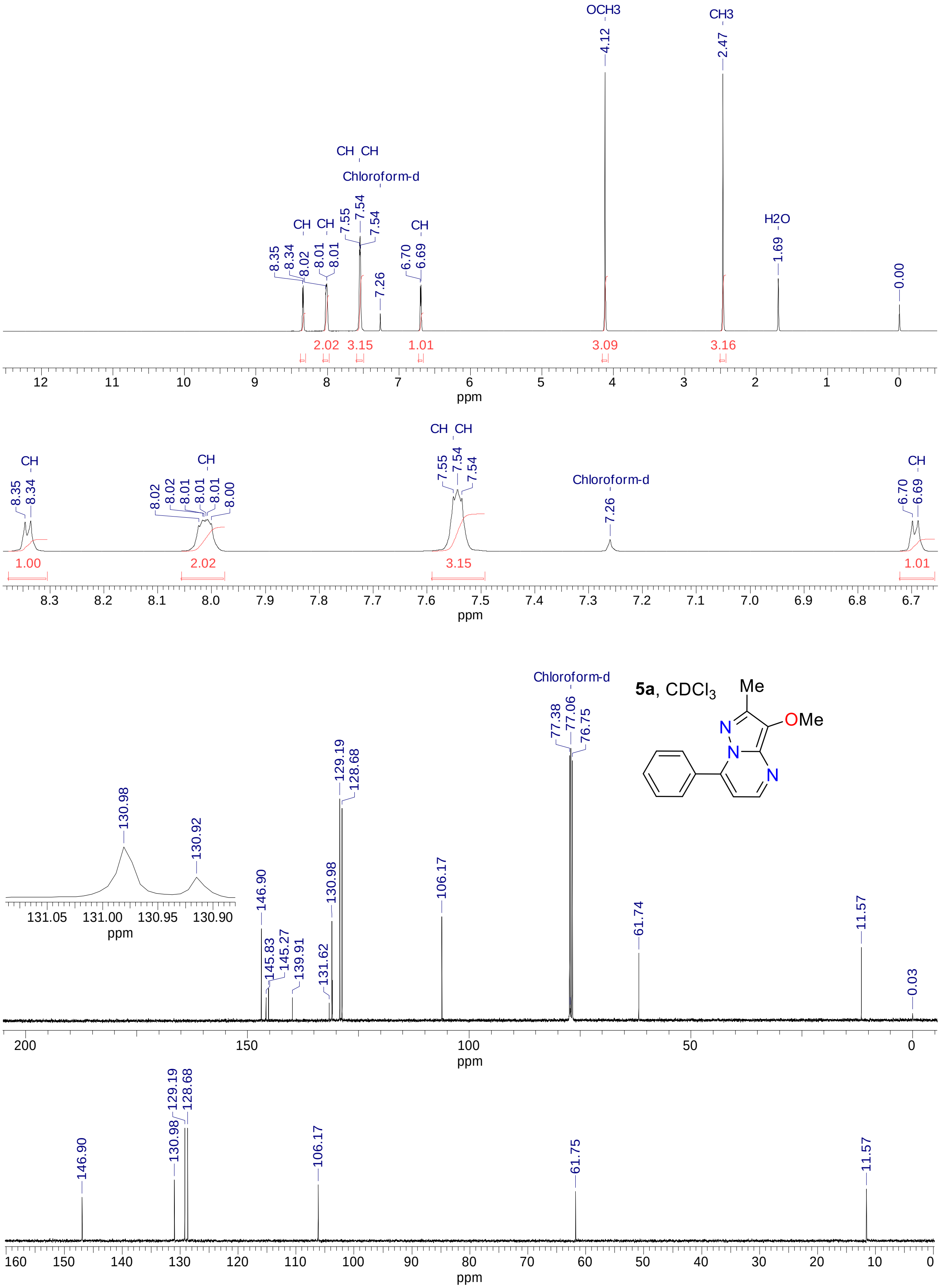

Fig. S69 $^{1}H/^{13}C$ NMR and DEPT-135 spectra of 3-methoxy-2-methyl-7-phenylpyrazolo[1,5-*a*]pyrimidine (**5a**).

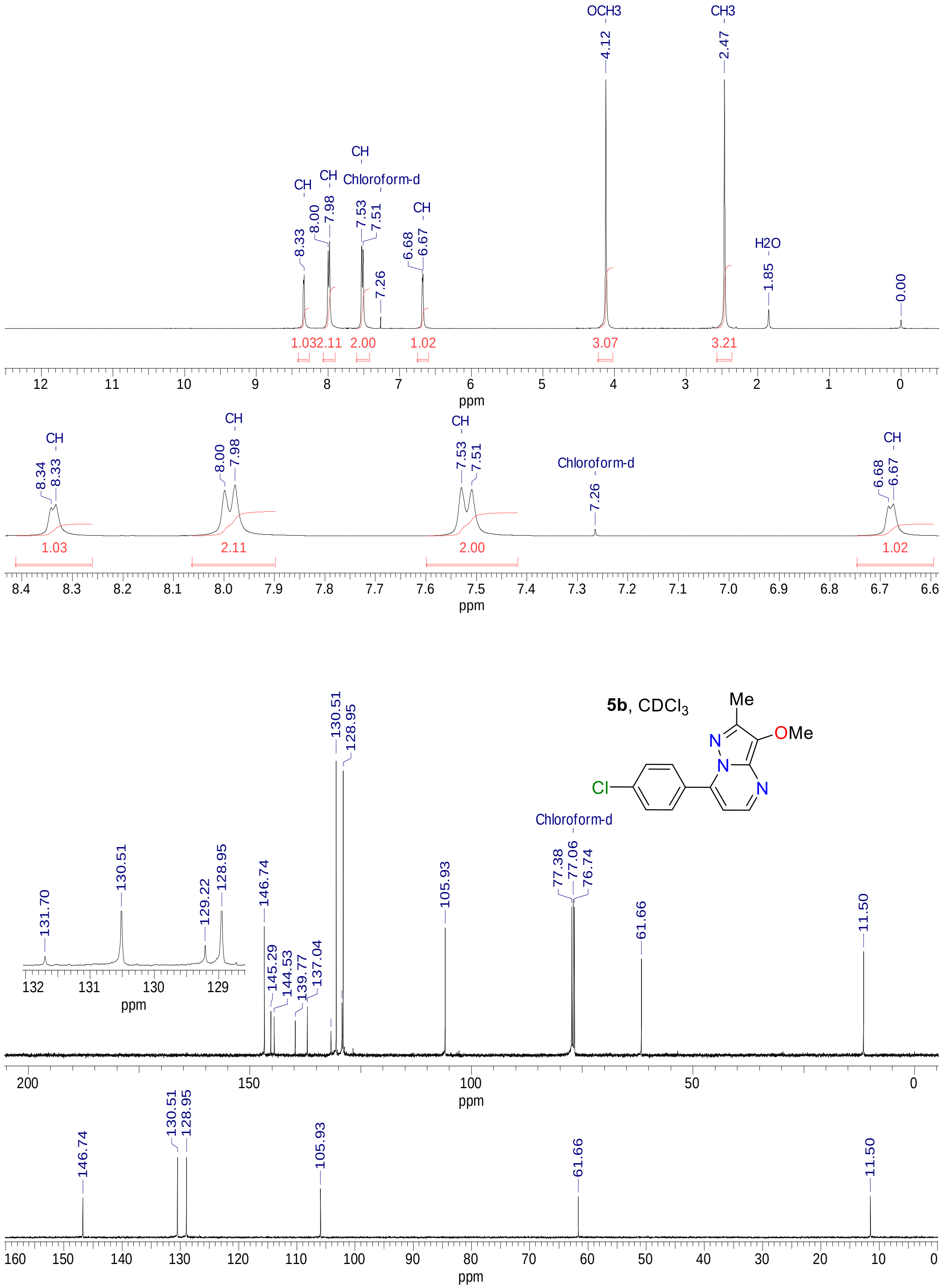

Fig. S70 $^{1}H/^{13}C$ NMR and DEPT-135 spectra of 7-(4-chlorophenyl)-3-methoxy-2-methyl-PP-3-ol (**5b**).

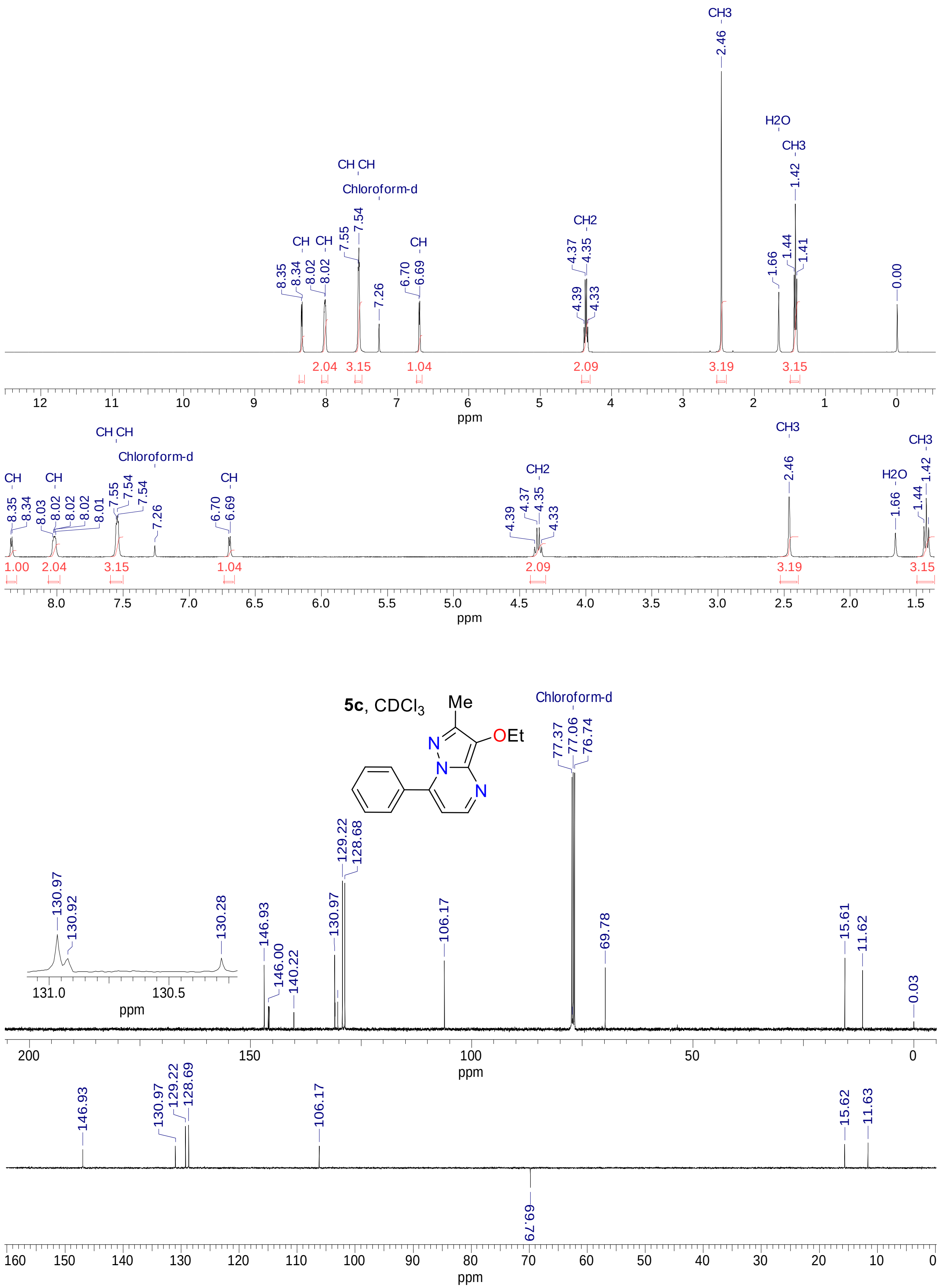

Fig. S71 $^{1}H/^{13}C$ NMR and DEPT-135 spectra of 3-ethoxy-2-methyl-7-phenylpyrazolo[1,5-*a*]pyrimidine (**5c**).

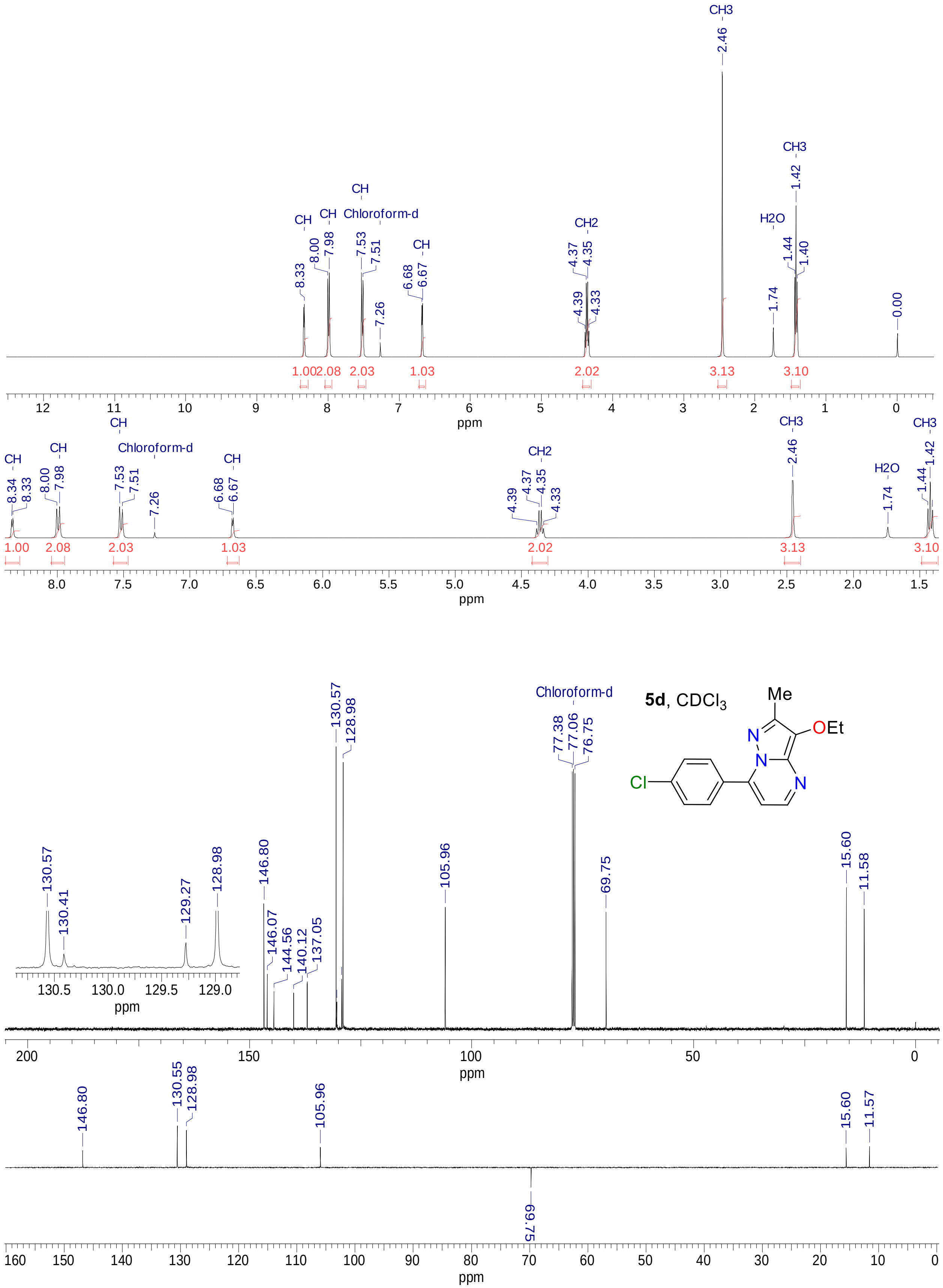

Fig. S72 $^{1}H/^{13}C$ NMR and DEPT-135 spectra of 7-(4-chlorophenyl)-3-ethoxy-2-methyl-Pp-3-ol (**5d**).

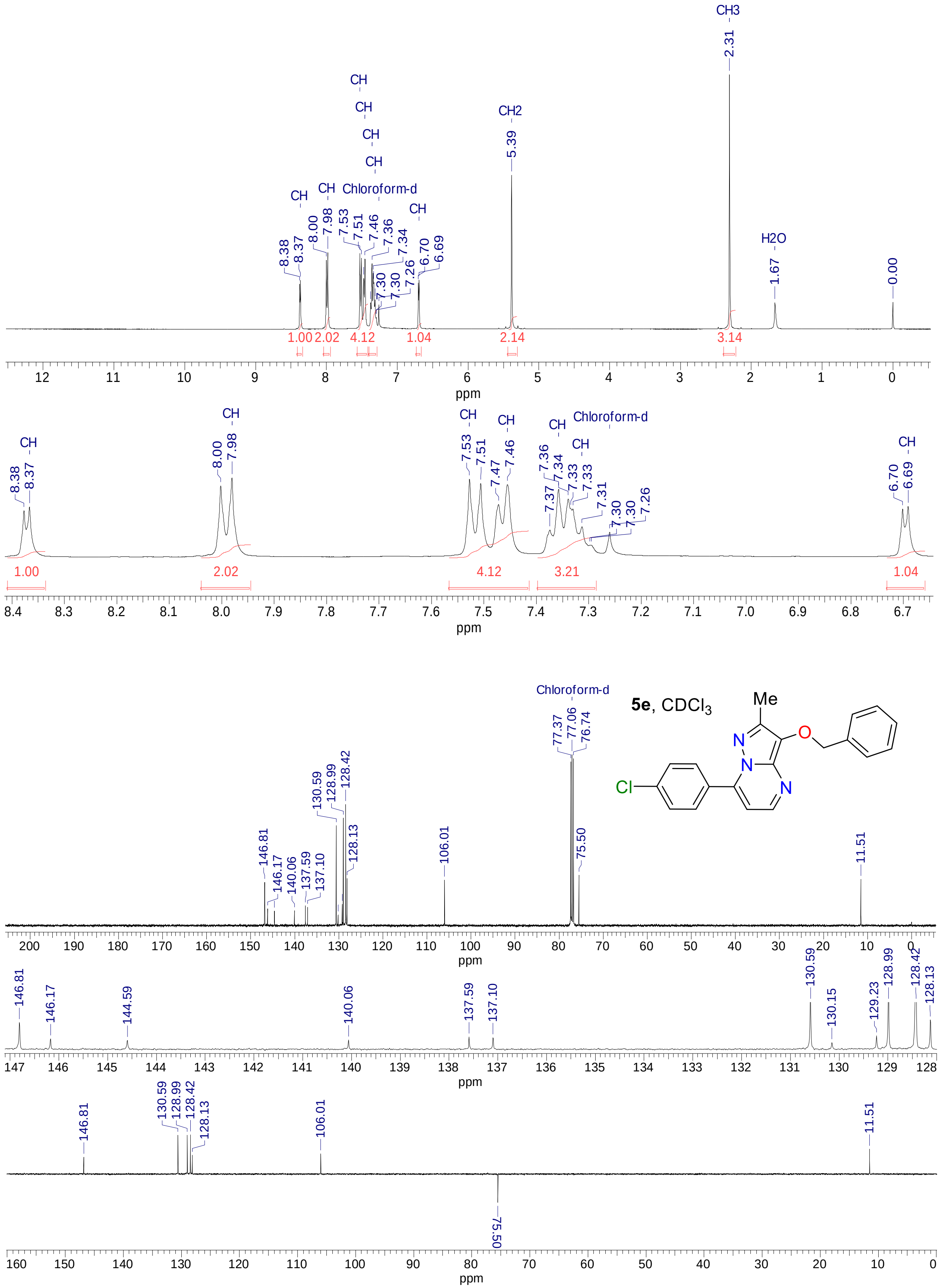

Fig. S73 $^{1}H/^{13}C$ NMR and DEPT-135 spectra of 3-benzyloxy-7-(4-chlorophenyl)-2-methyl-Pp-3-ol (**5e**).

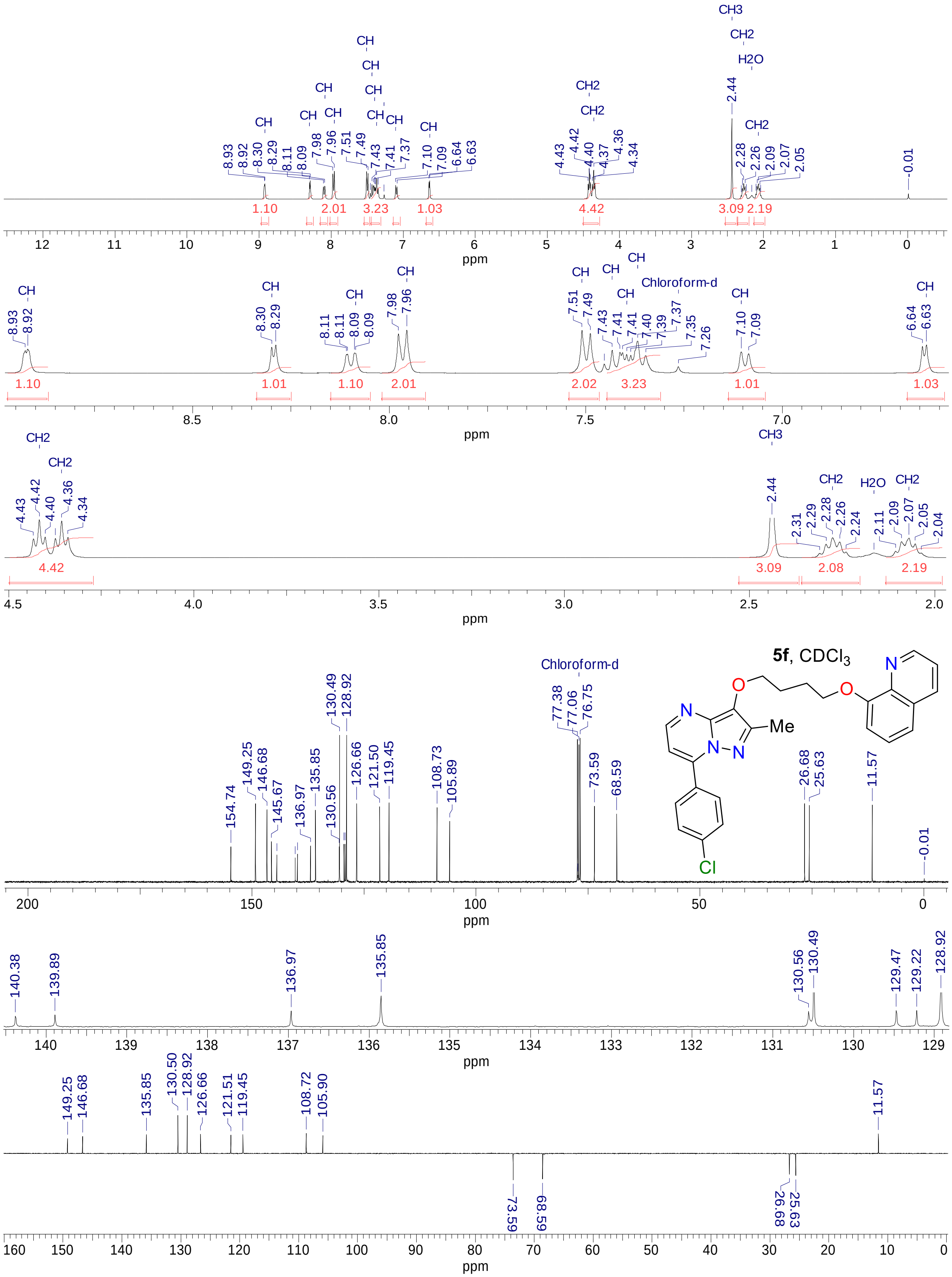

Fig. S74 ^{1}H/^{13}C NMR and DEPT-135 spectra of 7-(4-ClPh)-3-(4-(8-quinolinoxy)butoxy)-2-methyl-Pp (**5f**).

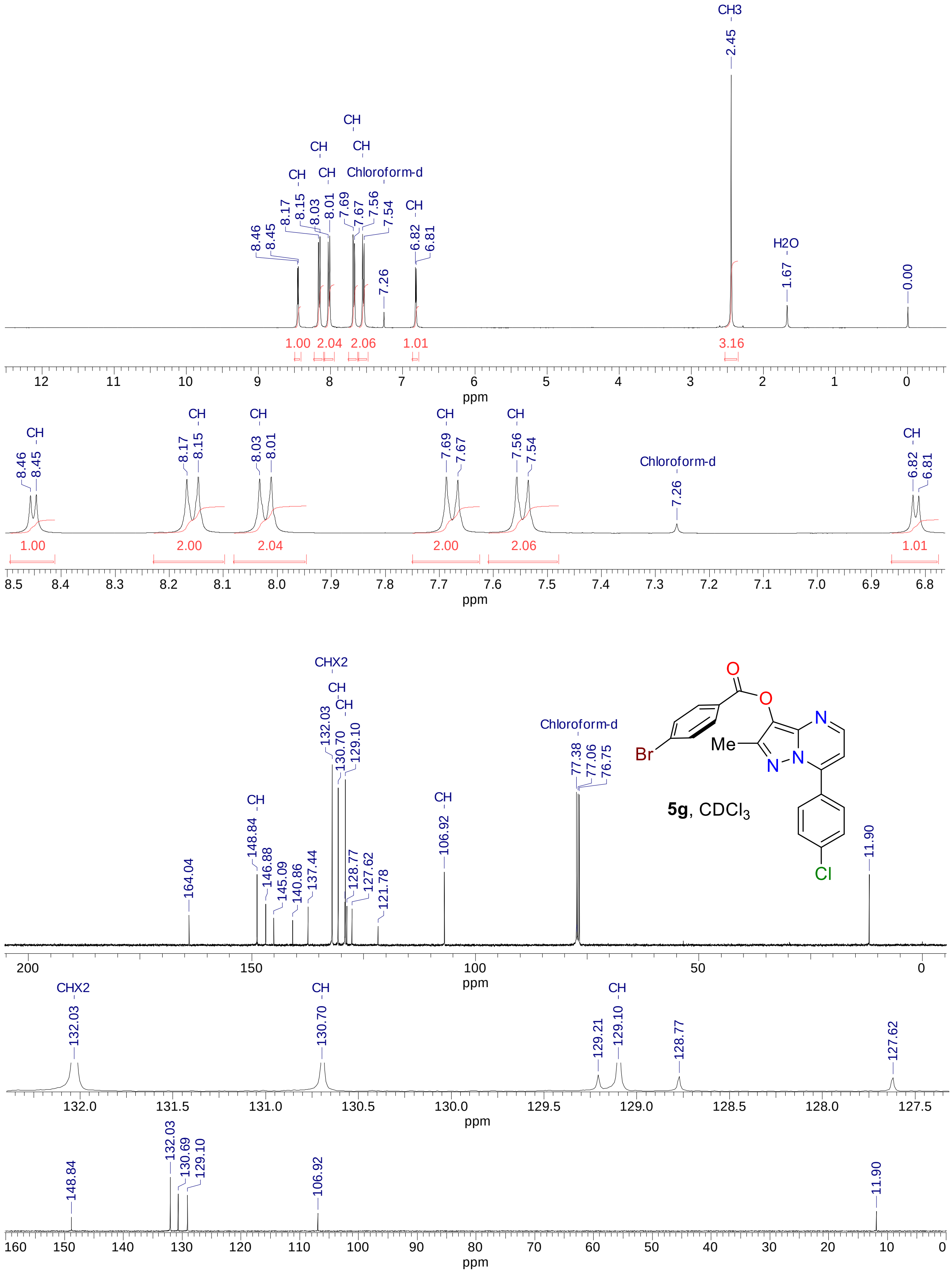

Fig. S75 ^{1}H/^{13}C NMR and DEPT-135 spectra of 3-benzyloxy-7-(4-chlorophenyl)-2-methyl-Pp-3-ol (**5g**).

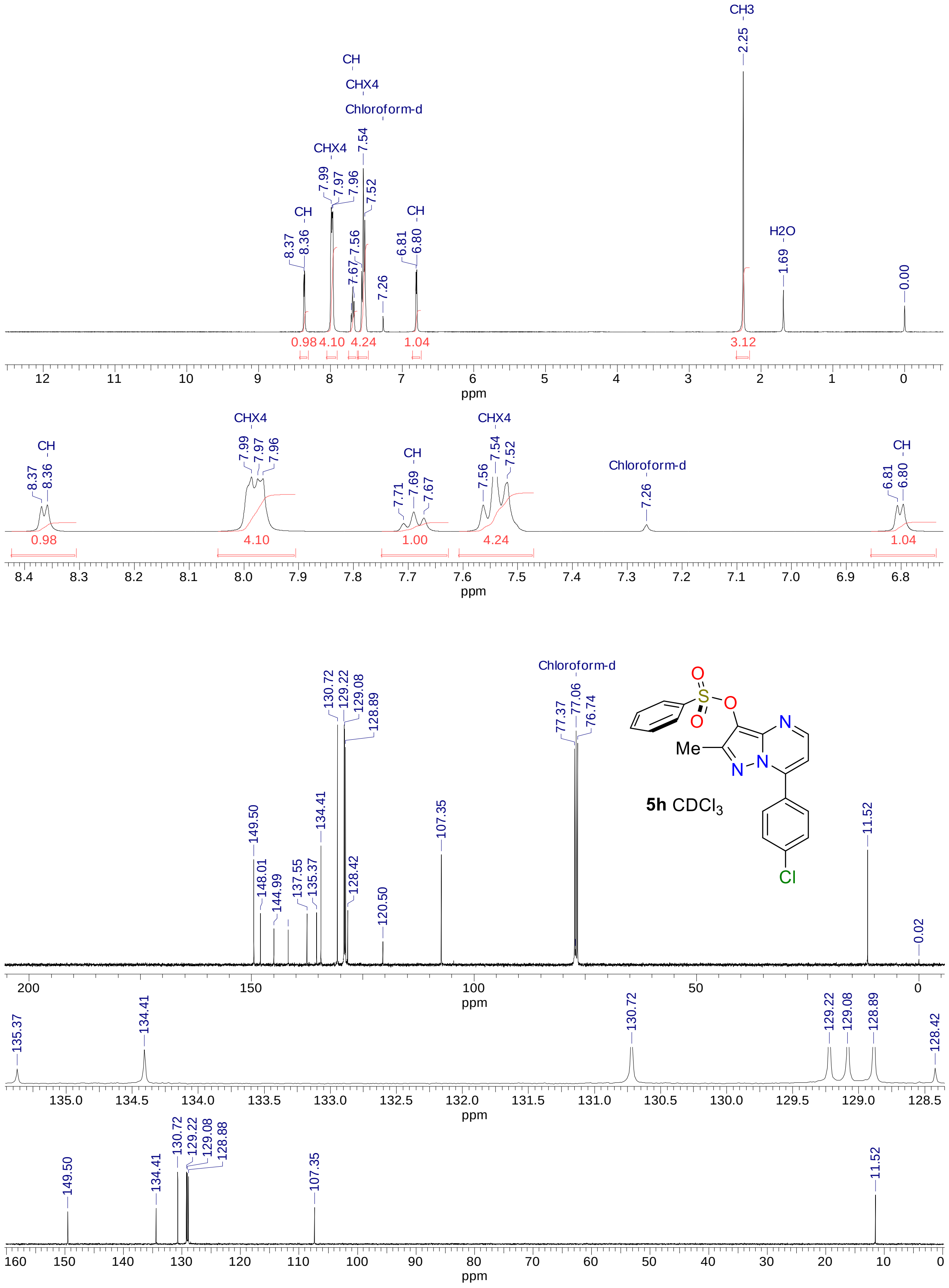

Fig. S76 $^{1}H/^{13}C$ NMR and DEPT-135 spectra of 3-bethoxy-2-methyl-7-phenyl-Pp (**5h**).

7. References

1 G. R. Fulmer, A. J. M. Miller, N. H. Sherden, H. E. Gottlieb, A. Nudelman, B. M. Stoltz, J. E. Bercaw and K. I. Goldberg, *Organometallics*, 2010, **29**, 2176–2179.

2 C. Cifuentes, N. Bravo, D. Restrepo, M. Macías and J. Portilla, *RSC Adv.*, 2025, **15**, 2078–2085.

3 A. Tigreros, S.-L. Aranzazu, N.-F. Bravo, J. Zapata-Rivera and J. Portilla, *RSC Adv.*, 2020, **10**, 39542–39552.

4 S. L. Aranzazu, A. Tigreros, A. Arias-Gómez, J. Zapata-Rivera and J. Portilla, *J. Org. Chem.*, 2022, **87**, 9839–9850.

5 J. C. Castillo, H. A. Rosero and J. Portilla, *RSC Adv.*, 2017, **7**, 28483–28488.

6 J.-C. Castillo, A. Tigreros and J. Portilla, *J. Org. Chem.*, 2018, **83**, 10887–10897.

7 M.-C. Ríos, N.-F. Bravo, M. Macías, B. A. Iglesias and J. Portilla, *ChemPhotoChem*, 2025, **2025**, e202400389.

8 J. C. Coa, E. García, M. Carda, R. Agutí, I. D. Vélez, J. A. Muñoz, L. M. Yepes, S. M. Robledo and W. I. Cardona, *Med. Chem. Res.*, 2017, **26**, 1405–1414.

9 J.-C. Castillo, A. Tigreros, C. Cifuentes and J. Portilla, *J. Org. Chem.*, 2024, **89**, 14606–14607.

10 K. Fujishiro and S. Mitamura, *Bull. Chem. Soc. Jpn.*, 1988, **61**, 4464–4466.

11 C. Quinton, V. Alain-Rizzo, C. Dumas-Verdes, G. Clavier, F. Miomandre and P. Audebert, *Eur. J. Org. Chem.*, 2012, **2012**, 1394–1403.

12 Z. Bazyar and M. Hosseini-Sarvari, *J. Org. Chem.*, 2019, **84**, 13503–13515.

13 P. L. Gkizis, C. T. Constantinou and C. G. Kokotos, *Eur. J. Org. Chem.*, DOI:10.1002/ejoc.202300898.

14 X. Zhang, G. Wu, W. Gao, J. Ding, X. Huang, M. Liu and H. Wu, *Org. Lett.*, 2018, **20**, 708–711.

15 M. E. El-Khouly, S. H. Shim, Y. Araki, O. Ito and K. Y. Kay, *Journal of Physical Chemistry B*, 2008, **112**, 3910–3917.

16 M. Faccini, M. Balakrishnan, M. B. J. Diemeer, Z. P. Hu, K. Clays, I. Asselberghs, A. Leinse, A. Driessen, D. N. Reinhoudt and W. Verboom, *J. Mater. Chem.*, 2008, **18**, 2141–2149.
